# GLUT9b- and ABCG2-mediated collecting duct urate transport uncovers a vasopressin-independent mechanism of renal water reabsorption

**DOI:** 10.1172/JCI197021

**Published:** 2026-06-16

**Authors:** Mohamad Hadla, Jean Marc Mardirossian, Daniel G. Bichet, Abdul Hamid Borghol, Georges Abboud, Ahmad Ghanem, Eduardo Chini, Peter Harris, Vicente E. Torres, Seth L. Alper, Volker Vallon, Fouad T. Chebib

**Affiliations:** 1 Division of Nephrology and Hypertension, Mayo Clinic, Jacksonville, Florida, USA.; 2 Departments of Medicine and of Pharmacology and Physiology, University of Montreal, Montreal, Quebec, Canada.; 3 Nephrology Service, Hôpital du Sacré-Cœur de Montréal, Montreal, Quebec, Canada.; 4 Metabolism and Molecular Nutrition Laboratory, Kogod Center on Aging, Department of Anesthesiology and Perioperative Medicine, Mayo Clinic College of Medicine, Jacksonville, Florida, USA.; 5 Division of Nephrology and Hypertension and; 6 Department of Biochemistry and Molecular Biology, Mayo Clinic, Rochester, Minnesota, USA.; 7 Division of Nephrology and Vascular Biology Research Center, Beth Israel Deaconess Medical Center, Boston, Massachusetts, USA.; 8 Department of Medicine, Harvard Medical School, Boston, Massachusetts, USA.; 9 Broad Institute of MIT and Harvard, Cambridge, Massachusetts, USA.; 10 Division of Nephrology and Hypertension, Department of Medicine, and; 11 Department of Pharmacology, UCSD, La Jolla, California, USA.; 12 VA San Diego Healthcare System, San Diego, California, USA.

**Keywords:** Cell biology, Nephrology, Clinical trials, Epithelial transport of ions and water, Genetic diseases

## Abstract

Renal water reabsorption is classically regulated by vasopressin V2 receptor (V2R) signaling through cyclic AMP and protein kinase A, driving apical accumulation of aquaporin-2 (AQP2). However, collecting duct water handling is also modulated by vasopressin-independent mechanisms. Here, we examined intracellular soluble urate as a vasopressin-independent regulator of AQP2 trafficking. Intracellular urate accumulation in collecting duct cells was mediated by enhanced apical urate uptake via GLUT9b and reduced apical urate efflux through ABCG2, triggering phosphodiesterase-4 activation, reduced cAMP, and downstream AMP-activated protein kinase (AMPK) activation. The resulting AQP2 accumulation at the apical membrane was independent of V2R signaling, required ongoing endocytosis, and was associated with features of postendocytic apical trafficking of internalized AQP2. In vivo ABCG2 inhibition with probenecid increased apical AQP2 abundance and markedly attenuated tolvaptan-induced polyuria in both wild-type and *Pkd1*^RC/RC^ autosomal dominant polycystic kidney disease (ADPKD) mice in a uricase-independent manner while preserving tolvaptan’s ADPKD-modifying efficacy. In a phase II trial with tolvaptan-treated patients with ADPKD, probenecid reduced urine volume and nocturia frequency. Together, these findings support a vasopressin-independent urate/AMPK/AQP2 pathway that regulates renal water handling and, in a preclinical ADPKD model, can uncouple cyst growth attenuation from the dose-limiting aquaretic effects of V2R antagonism.

## Abstract 2

We identify a vasopressin-independent kidney pathway regulating water balance, offering a strategy to reduce tolvaptan-related side effects in polycystic kidney disease.

## Graphical abstract

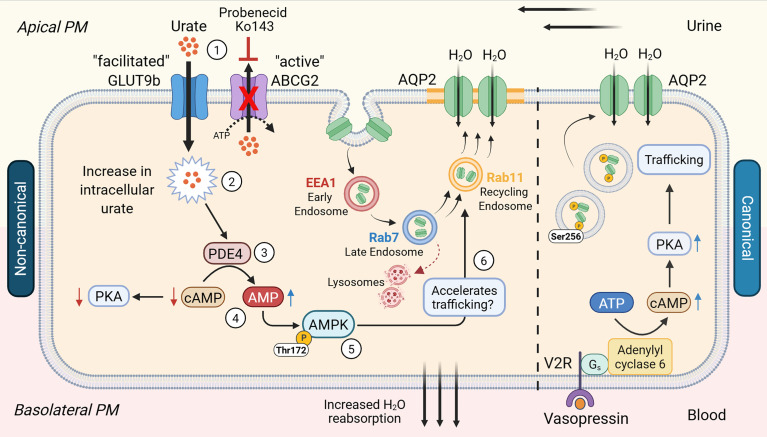

## Introduction

Renal water regulation is fundamental for cellular and systemic homeostasis (1–3) and is executed primarily in the collecting duct, where principal cells dynamically adapt apical membrane water permeability through regulated trafficking of the water channel aquaporin-2 (AQP2). Hypothalamic arginine vasopressin (AVP) activates V2 receptors (V2Rs) on collecting duct principal cells in the canonical signaling pathway to stimulate adenylyl cyclase 6, increase cAMP, and activate PKA, which phosphorylates AQP2 at Ser256, a modification associated with increased apical accumulation and membrane retention of AQP2 (2–7). Additional phosphorylation at Ser269 contributes to AQP2 stabilization at the apical membrane (8–10). AVP signaling also remodels the cortical actin cytoskeleton through RhoA to facilitate vesicle access to the apical membrane, while ubiquitination-regulated sorting pathways direct internalized AQP2 toward recycling or degradation (11, 12). Together, these mechanisms integrate rapid trafficking control with longer term regulation of channel abundance to fine-tune collecting duct water permeability (13).

Alongside this AVP/V2R/cAMP/PKA axis, AQP2 trafficking is regulated by multiple AVP-independent pathways that expand the signaling architecture governing water reabsorption. Inhibition of epidermal growth factor receptor (EGFR) signaling (14), modulation of RhoA activity by statins or fluconazole, and activation of AMP-activated protein kinase (AMPK) by metformin or direct agonists (15, 16) each promotes apical AQP2 accumulation and can improve urinary concentration in vasopressin-resistant states. Additional hormonal inputs, including secretin, glucagon, serotonin, calcitonin, and prostaglandin E_2_, regulate AQP2 through discrete signaling microdomains, some independent of global cAMP elevation (17–22). These observations establish that principal cells integrate diverse hormonal, paracrine, and metabolic cues to regulate the steady-state distribution of AQP2 through constitutive exocytic delivery, endocytic retrieval, and surface stability.

This expanded regulatory landscape raises the possibility that endogenous metabolites may also function as intrinsic modulators of AQP2 trafficking. Urate, the terminal product of purine metabolism, represents a plausible candidate (23–26). Humans uniquely lack hepatic uricase, resulting in higher circulating urate concentrations than in other mammals, an evolutionary feature proposed to support antioxidant capacity and conservation of water and salt (27, 28). Renal urate handling has classically been assigned to the proximal tubule, where URAT1, GLUT9a, ABCG2, and other transporters coordinate reabsorption and secretion (29–31). However, the GLUT9b isoform localizes to the apical membrane of human collecting duct principal cells, where it functions as a high-capacity urate transporter (32). ABCG2 is also expressed in principal cells and mediates ATP-dependent urate efflux (31). Together, these transporters establish opposing influx and efflux pathways that enable bidirectional urate movement across the apical membrane of the nephron segment responsible for regulated water reabsorption.

Genetic and physiological evidence further links urate handling to water balance (24, 33). Loss-of-function mutations in *GLUT9* cause renal hypouricemia with hyperuricosuria and exercise-induced acute kidney injury (24, 34). Global or kidney-specific deletion of *Glut9* in mice results in marked polyuria and impaired urine concentration, despite intact V2R signaling and preserved corticomedullary osmotic gradient (33, 35). Conversely, clinical antidiuretic states such as the syndrome of inappropriate antidiuresis (SIAD) are characterized by hypouricemia and elevated fractional excretion of urate (36). In a rat model of SIAD, renal urate transporter expression is reciprocally regulated, with reduced GLUT9 and increased ABCG2 expression (37). Intracellular urate has been previously shown to inhibit Akt (38) and activate AMPK, a kinase that directly modulates AQP2 trafficking and promotes water reabsorption in AVP-resistant states (39).

The absence of serum uricase in humans, the localization of urate transporters in collecting duct principal cells, the reciprocal alterations in urate handling across polyuric and antidiuretic states, and the capacity of urate to engage AMPK-dependent pathways converge on the hypothesis that soluble urate functions as an endogenous regulator of AQP2 trafficking. In this study, we test this hypothesis by delineating how urate transport, intracellular urate dynamics, and urate-responsive signaling pathways govern AQP2 localization, collecting duct water permeability, and clinically relevant polyuric states.

## Results

### Apical urate increases intracellular urate and induces concentration-dependent apical accumulation of AQP2 independently of V2R signaling.

**Figure 1:**
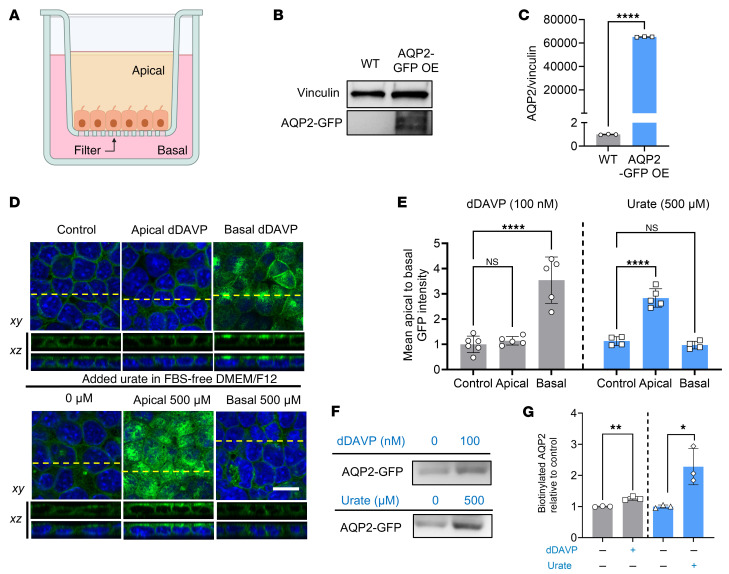
Urate induces apical trafficking of AQP2 in polarized collecting duct cells. (**A**) Schematic of polarized mIMCD3 cells stably expressing AQP2-GFP, grown on Transwell inserts. (**B**) Immunoblot confirming AQP2-GFP overexpression in the stable cell line. (**C**) Densitometric quantitation of AQP2-GFP as in **B**. (**D**) Confocal images of cells treated for 1 hour with apical urate (500 μM) or basolateral dDAVP (100 nM), showing apical AQP2 accumulation; *Z*-scan planes indicated by yellow dashed lines. (**E**) Normalized apical-to-basal AQP2-GFP fluorescence intensity (FI) ratios as in **D**. (**F**) Immunoblot of biotinylated surface AQP2-GFP. (**G**) Densitometric quantitation showing increased plasma membrane AQP2 after 1-hour exposure to dDAVP or apical urate. Data are means ± SD; *n* ≥ 3. Unpaired *t* test (**C** and **G**) and 2-way ANOVA with Dunnett’s correction (**E**). *P < 0.05, **P < 0.01, ****P < 0.0001. Scale bar, 25 μm.

**Figure 2:**
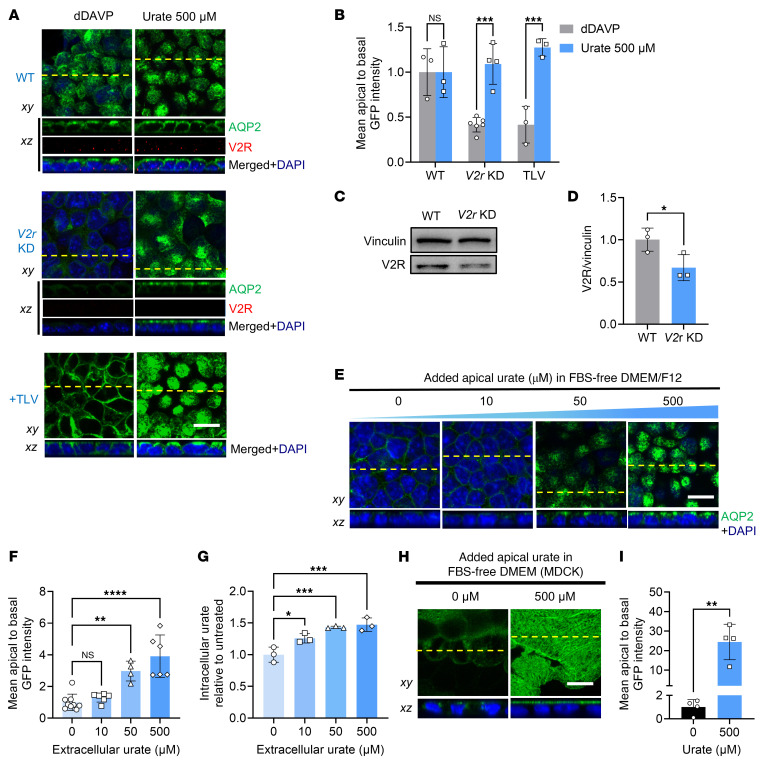
Urate induces concentration-dependent, V2R-independent apical trafficking of AQP2 in polarized collecting duct cells. (**A**) Confocal imaging of AQP2-GFP showing that apical urate (500 μM, 1 hour) induced apical AQP2 accumulation even after *V2r* knockdown (*V2r* KD) or tolvaptan pretreatment, unlike with dDAVP. (**B**) Normalized apical-to-basal AQP2-GFP FI ratios as in **A**. (**C**) Immunoblot validation of *V2r*-KD cells. (**D**) Densitometric quantitation as in **C**. (**E**) Confocal images showing dose-dependent AQP2 apical with increasing urate concentrations (1 hour). (**F**) Normalized apical-to-basal AQP2-GFP FI ratios as in **E**. (**G**) Control-normalized intracellular urate after 1-hour exposure to indicated extracellular urate concentrations. (**H**) Confocal images showing apical urate (500 μM, 1 hour) induced AQP2 apical accumulation in MDCK cells. (**I**) Normalized apical-to-basal AQP2-GFP FI ratios as in **H**. Urate treatments were in FBS-free medium. Data are mean ± SD; *n* ≥ 3. Unpaired *t* test (**D** and **I**); 2-way ANOVA with Bonferroni’s correction (**B**); 1-way ANOVA with Dunnett’s correction (**F** and **G**). *P < 0.05, **P < 0.01, ***P < 0.001, ****P < 0.0001. Scale bar, 25 μm.

To determine whether urate directly regulates AQP2 trafficking, we used polarized murine inner medullary collecting duct (mIMCD3) cells stably expressing green fluorescence protein–tagged (GFP-tagged) AQP2 (Figure 1, A–C). [Deamino-Cys^1^, D-Arg^8^]-Vasopressin (dDAVP) (100 nM) induced robust apical accumulation of AQP2 (Figure 1, D and E). Apical urate (500 μM) produced a similar redistribution of AQP2 to the apical surface, demonstrated by confocal microscopy and surface biotinylation (Figure 1, D–G). To test whether this requires canonical V2R signaling, V2R expression was reduced by siRNA or inhibited with tolvaptan (100 nM). Both interventions blocked dDAVP-induced AQP2 translocation but had no effect on urate-induced AQP2 accumulation (Figure 2, A–D), establishing urate as a V2R-independent stimulus.

We next examined the relationship between intracellular urate and AQP2 trafficking. Intracellular urate increased proportionally with rising extracellular apical urate. Apical AQP2 accumulation followed a concentration-response relationship, with increased AQP2 detectable at 50 μM extracellular apical urate (Figure 2, E–G). Similar urate-induced trafficking of AQP2-GFP was observed in Madin-Darby Canine Kidney (MDCK) cells (Figure 2, H and I). C-terminal GFP tagging of the AQP2 did not alter its trafficking, as dDAVP and urate induced comparable apical AQP2 accumulation in cells expressing untagged AQP2 (Supplemental Figure 1, A and B; supplemental material available online with this article; https://doi.org/10.1172/JCI197021DS1). Under all experimental conditions, urate remained soluble and nonfluorescent (Supplemental Figure 1, C–E). Rasburicase, which enzymatically converts urate to allantoin, eliminated detectable urate from culture media and abolished AQP2 translocation (Supplemental Figure 1, F–H), confirming specificity of both the urate assay and the apical trafficking response for urate itself rather than a contaminant or degradation product. Mouse urinary urate concentrations of 240–480 μM (40, 41) overlapped with concentrations inducing apical AQP2 trafficking. As 500 μM urate reproducibly remained soluble and elicited maximal responses, that concentration was used in subsequent studies.

### Apical GLUT9 and ABCG2 govern intracellular urate levels and thereby control AQP2 trafficking.

**Figure 3:**
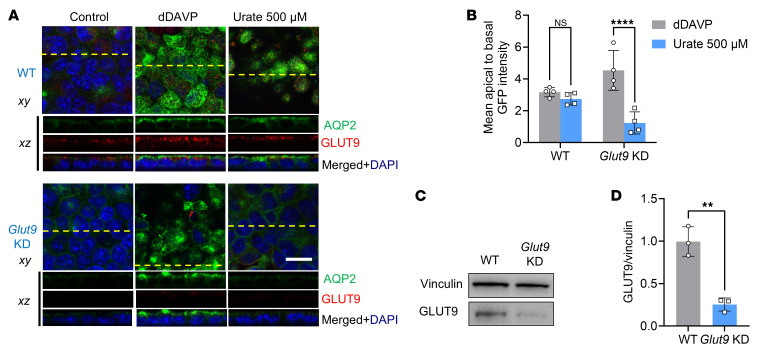
Urate-induced AQP2 trafficking requires GLUT9-mediated influx. (**A**) Confocal images of polarized mIMCD3 AQP2-GFP cells with corresponding XZ FI (yellow dashed lines), showing apical GLUT9 localization. (**B**) Apical-to-basal AQP2-GFP FI ratios in wild-type and *Glut9*-knockdown (KD) cells, showing greater urate-induced reduction of apical AQP2-GFP FI in *Glut9*-KD cells. (**C**) Immunoblot of GLUT9 in wild-type and *Glut9*-KD cells. (**D**) Densitometric quantitation as in **C**. Urate treatments were in FBS-free medium. Data are means ± SD; *n* ≥ 3. Unpaired *t* test (**D**); 2-way ANOVA with Bonferroni’s correction (**B**). **P < 0.01, ****P < 0.0001. Scale bar, 25 μm.

**Figure 4:**
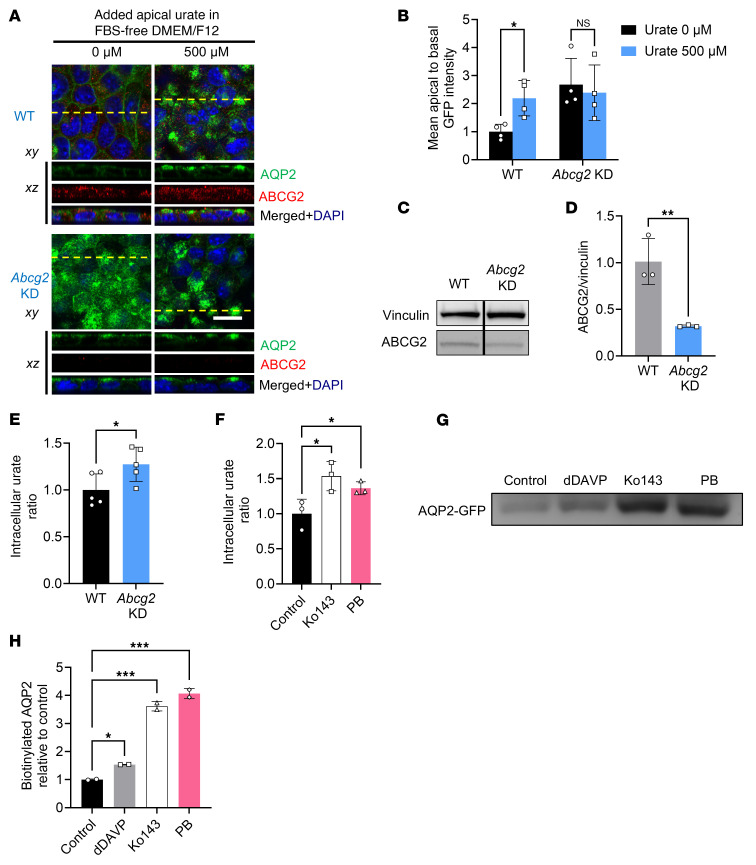
Urate-induced AQP2 trafficking is potentiated by inhibition of ABCG2-mediated efflux. (**A**) Confocal images of AQP2-GFP showing that *Abcg2* knockdown (KD) induced apical AQP2 accumulation. (**B**) Normalized apical-to-basal AQP2-GFP FI ratios. (**C**) Immunoblot of ABCG2 in wild-type and *Abcg2*-KD cells. Noncontiguous lanes from the same gel are shown. (**D**) Densitometric quantitation of *Abcg2* KD as in **C**. (**E** and **F**) Intracellular urate levels in (**E**) *Abcg2* KD vs. wild-type cells and (**F**) wild-type cells treated for 1 hour with Ko143 (1 μM) or probenecid (PB, 100 μM). (**G**) Immunoblot of surface-biotinylated AQP2-GFP following Ko143 or PB treatment. (**H**) Densitometric quantitation showing increased membrane AQP2 after Ko143 or PB. Urate treatments were in FBS-free medium. Data are means ± SD; *n* ≥ 3. Unpaired *t* test (**D** and **E**), 2-way ANOVA with Bonferroni’s correction (**B**), and 1-way ANOVA with Dunnett’s correction (**F** and **H**). *P < 0.05, **P < 0.01, ***P < 0.001. Scale bar, 25 μm.

To define the mechanism by which urate regulates AQP2, we examined the roles of 2 urate transporters localized to the apical membrane of collecting duct principal cells, GLUT9b and ABCG2. Knockdown of *Glut9* did not alter basal or dDAVP-induced AQP2 localization but abolished urate-induced AQP2 apical accumulation (Figure 3, A–D), identifying GLUT9 as a dominant pathway mediating luminal urate entry. In contrast, *Abcg2* knockdown in the absence of added exogenous urate triggered spontaneous AQP2 membrane insertion, accompanied by elevated intracellular urate levels (Figure 4, A–E). These findings support a model in which ABCG2 functions as an apical urate efflux transporter such that loss of ABCG2 reduces urate export, promotes intracellular urate retention, and facilitates AQP2 apical trafficking.

**Figure 5:**
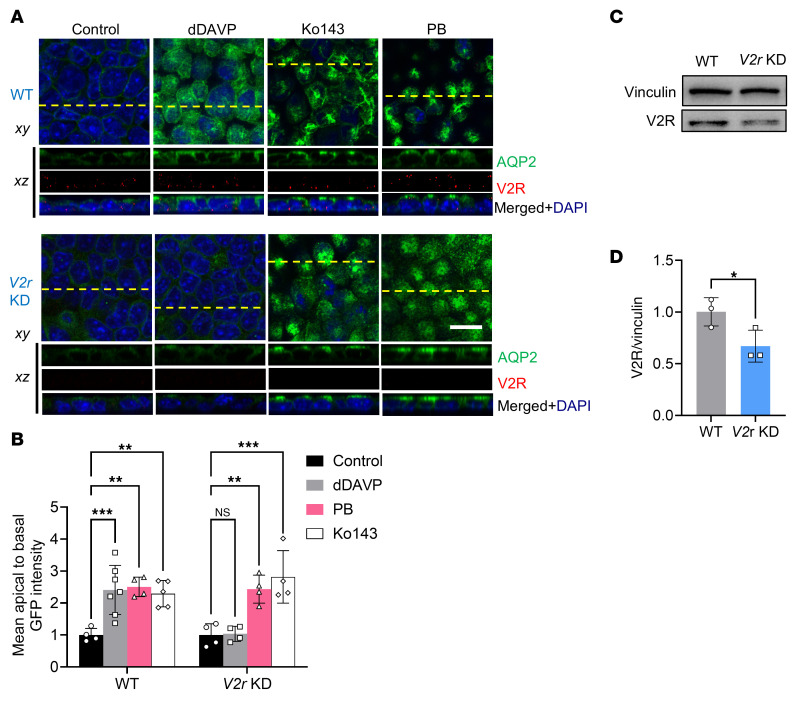
Pharmacological inhibition of ABCG2 induces apical AQP2 accumulation in a V2R-independent manner. (**A**) Confocal images of AQP2-GFP localization in wild-type and *V2r*-knockdown (KD) cells treated for 1 hour with dDAVP (100 nM), Ko143 (1 μM), or probenecid (PB, 100 μM). (**B**) Normalized apical-to-basal AQP2-GFP FI ratios showing that Ko143 and probenecid induced apical AQP2 accumulation in *V2r*-KD cells. (**C**) Immunoblot of V2R expression in wild-type and *V2r*-KD cells. (**D**) Densitometric quantitation as in **C**. Data are mean ±SD; *n* ≥ 3. Unpaired *t* test (**D**); 2-way ANOVA with Dunnett’s correction (**B**). *P < 0.05, **P < 0.01, ***P < 0.001. Scale bar, 25 μm.

**Figure 6:**
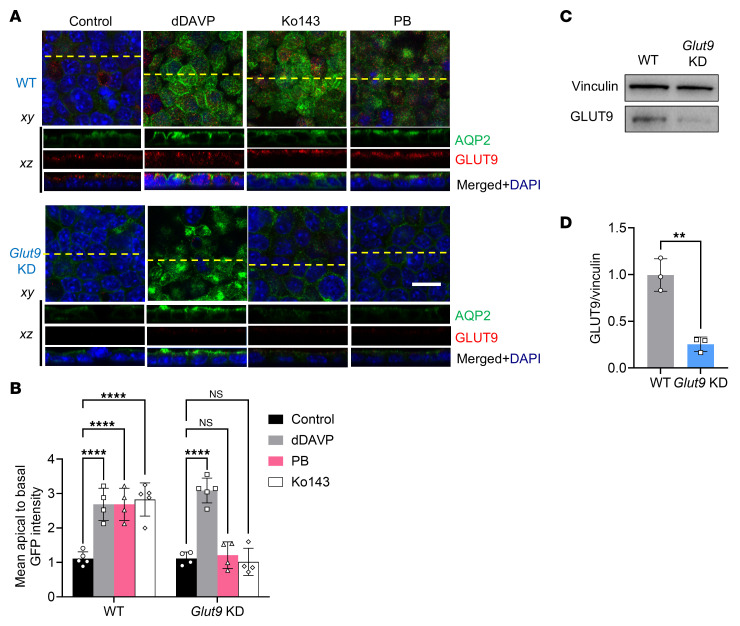
Pharmacological inhibition of ABCG2 induces apical AQP2 accumulation in a GLUT9-dependent manner. (**A**) Confocal images of AQP2-GFP localization. (**B**) Normalized apical-to-basal AQP2-GFP FI ratios showing that Ko143 (1 μM) or probenecid (PB, 100 μM, 1 hour) induces apical AQP2 accumulation in a GLUT9-dependent manner. (**C**) Immunoblot of GLUT9 in wild-type and *Glut9*-knockdown (KD) cells. (**D**) Densitometric quantitation as in **C**. Data are means ± SD; *n* ≥ 3. Unpaired *t* test (**D**); 2-way ANOVA with Dunnett’s correction (**B**). **P < 0.01, ****P < 0.0001. Scale bar, 25 μm.

Short-term (1-hour) pharmacological inhibition of ABCG2 with either Ko143 or probenecid similarly increased intracellular urate levels (Figure 4F) and induced robust AQP2 apical membrane accumulation, confirmed by surface biotinylation (Figure 4, G and H) and confocal imaging (Figure 5, A and B). This response was V2R independent (Figure 5, A–D) but required GLUT9, as concurrent *Glut9* knockdown abolished AQP2 accumulation (Figure 6, A–D).

Loss of ABCG2 function also counteracted effects of V2R antagonism. *Abcg2* knockdown restored AQP2 accumulation in the presence of tolvaptan, and ABCG2 inhibition by probenecid preserved apical AQP2 accumulation even during combined exposure to dDAVP and tolvaptan (Supplemental Figure 2, A and B). Chemically distinct ABCG2 inhibitors including benzbromarone, resveratrol, febuxostat, and novobiocin reproduced AQP2 apical accumulation in a GLUT9-dependent manner (Supplemental Figure 2, C and D).

In GLUT9-deficient cells, acute (1-hour) inhibition of ABCG2 with Ko143 or probenecid did not increase intracellular urate or induce apical AQP2 trafficking; however, prolonged inhibition (12–24 hours) resulted in both effects (Supplemental Figure 3, A–D). Similar findings were observed in MDCK cells (Supplemental Figure 4, A and B). Immunohistochemistry of human kidney sections confirmed luminal ABCG2 and apical GLUT9 localization in collecting ducts (Supplemental Figure 4C).

To test whether increased apical urate influx is sufficient to regulate AQP2 trafficking, we overexpressed GLUT9b in mIMCD3 cells. The impact of overexpressing a facilitative transporter such as GLUT9b is context dependent, reflecting the chemical (and maybe also electrical) gradients determining the balance between inward and outward urate fluxes across the apical membrane (42, 43). Generation of stable GLUT9b*-*overexpressing cells required prolonged culture with sustained exposure to the low micromolar urate in FBS (~15 μM urate in 10% FBS, corresponding to ~7–8 μM in 5% FBS) (Supplemental Figure 4D and Methods). During this period, apical ABCG2–mediated outward pumping of reabsorbed and endogenously generated urate remains intact, maintaining low steady-state intracellular urate levels. Under these conditions, an inward-directed urate concentration gradient may favor increased uptake with GLUT9 overexpression, fostering intracellular urate accumulation. Indeed, previous studies of GLUT9b overexpression in kidney-derived cells showed at least 2-fold increase in cellular urate uptake, an effect diminished by GLUT9 knockdown (30). Accordingly, GLUT9b overexpression increased intracellular urate levels under standard culture conditions and induced robust apical accumulation of AQP2 (Supplemental Figure 4, E–G). AQP2 remained apically localized despite concurrent exposure to tolvaptan and dDAVP, indicating that elevated intracellular urate can maintain AQP2 localization even when V2R signaling is inhibited (Supplemental Figure 4, E–G). Although serum was acutely removed before trafficking assays, the brief (1-hour) deprivation period appeared insufficient to reverse intracellular urate accumulation established during prolonged culture. Incremental exogenous urate did not further increase apical AQP2 accumulation under GLUT9b-overexpressing conditions (Supplemental Figure 4, E–G). GLUT9b overexpression produced a 2-fold increase in GLUT9 expression with a modest 1.2-fold increase in ABCG2 (Supplemental Figure 4, H and I), suggesting partial compensation. However, this increase in urate efflux capacity appeared insufficient to offset enhanced urate influx, reflecting the persistently elevated intracellular urate levels in GLUT9b-overexpressing cells.

To assess whether probenecid acts through targets beyond ABCG2, we conducted siRNA-mediated knockdown of the broad-spectrum solute channel PANNEXIN-1, implicated in purinergic signaling, and the probenecid-sensitive inflammasome component NLRP3. Neither knockdown attenuated probenecid-induced apical AQP2 accumulation (Supplemental Figure 5, A–F), arguing against contributions from these pathways. We next asked whether urate-dependent AQP2 trafficking requires function of urea transporter UT-A, a contributor to establishment and maintenance of the corticomedullary osmotic gradient. Apical urate induced robust AQP2 redistribution in UT-A–deficient mIMCD3 cells, with kinetics and magnitude comparable to wild-type cells, in both the absence and presence of exogenous urea (800 mM). Acute urea exposure alone increased neither apical AQP2 abundance nor its stimulation by urate (Supplemental Figure 6, A–D). These findings suggest that urate-dependent AQP2 trafficking proceeds independently of UT-A–mediated urea transport.

Intracellular urate levels are, thus, determined by coordinated GLUT9-mediated influx and ABCG2-mediated efflux, defining a transport-coupled, vasopressin-independent regulatory axis controlling AQP2 trafficking.

### Urate induces AQP2 trafficking independently of Ser256 phosphorylation while suppressing PKA activity and intracellular cAMP levels.

**Figure 7:**
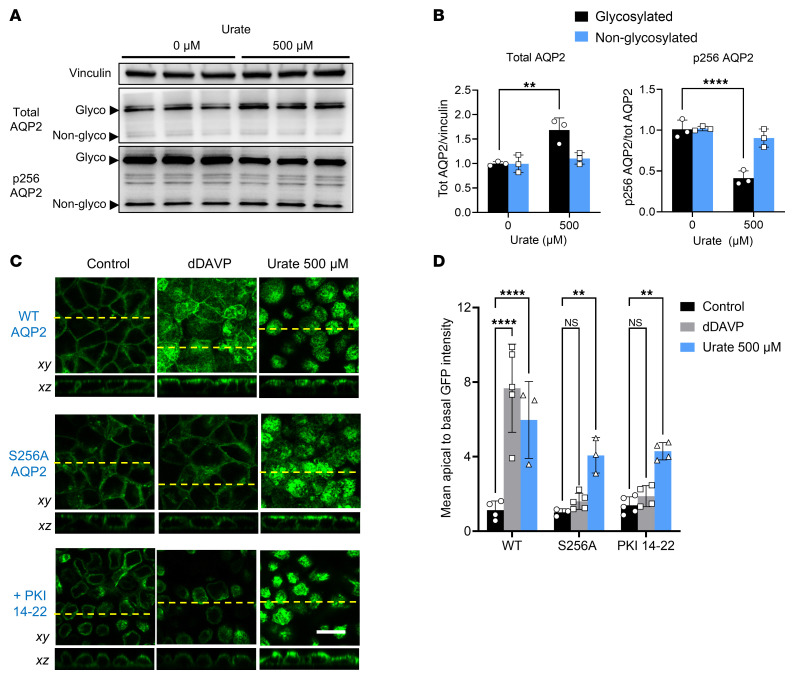
Urate-induced AQP2 translocation is independent of Ser256 phosphorylation. (**A**) Immunoblot of total and pSer256 AQP2 in mIMCD3 cells expressing AQP2-GFP after 1-hour treatment ± urate (500 μM). (**B**) Densitometric quantitation showing increased glycosylated AQP2 (61–71 kDa) and reduced pSer256 AQP2, with unchanged nonglycosylated AQP2. (**C**) Confocal images of AQP2-GFP cells expressing wild-type or S256A mutant AQP2, ± dDAVP (100 nM) or apical urate (500 μM, 1 hour). Pretreatment with PKA inhibitor PKI 14-22 (10 μM, 30 minutes) blocked dDAVP- but not urate-induced AQP2 accumulation. (**D**) Normalized apical-to-basal AQP2-GFP FI ratios as in **C**. Urate treatments were in FBS-free DMEM/F12. Data are means ± SD; *n* ≥ 3. Two-way ANOVA with Bonferroni’s correction (**B**) and Dunnett’s correction (**D**). **P < 0.01, ****P < 0.0001. Scale bar, 25 μm.

To check if urate-induced AQP2 trafficking engages the cAMP/PKA pathway, we assessed in polarized, AQP2-GFP–expressing mIMCD3 cells AQP2 phosphorylation at its principal PKA target site, Ser256, together with global PKA activity and intracellular cAMP levels. Apical urate exposure (500 μM, 1 hour) reduced Ser256 phosphorylation and increased the glycosylated, membrane-associated AQP2 pool, without affecting nonglycosylated AQP2 (Figure 7, A and B).

**Figure 8:**
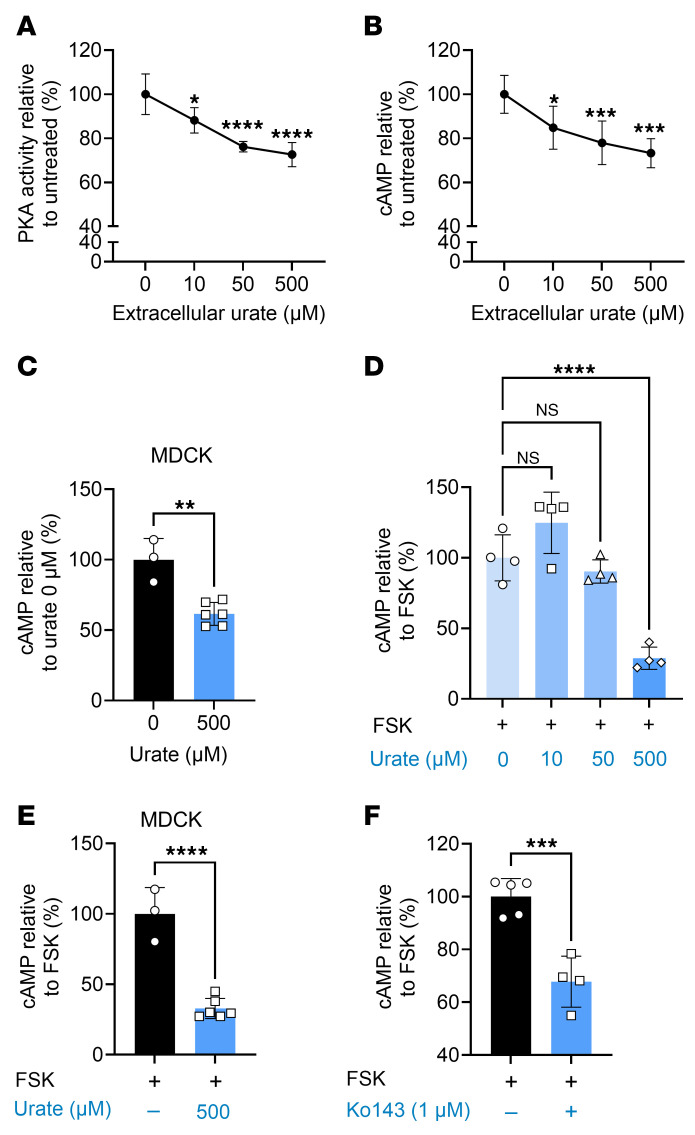
Urate-induced AQP2 translocation is independent of PKA activity and intracellular cAMP. (**A** and **B**) Normalized PKA activity (**A**) and cAMP levels (**B**) in mIMCD3 cells after 1-hour urate exposure, showing concentration-dependent decreases. (**C**) Normalized intracellular cAMP levels in MDCK cells after 1-hour urate exposure. (**D**) Normalized intracellular cAMP levels in mIMCD3 cells pretreated with forskolin (FSK, 10 μM, 30 minutes) followed by 1-hour urate exposure. (**E**) Normalized intracellular cAMP levels in MDCK cells pretreated with FSK (10 μM, 30 minutes) followed by 1-hour exposure to urate (500 μM). (**F**) Normalized intracellular cAMP levels in mIMCD3 cells pretreated with FSK (10 μM, 30 minutes) followed by 1-hour Ko143 treatment. All urate treatments were in FBS-free medium. Data are means ± SD; *n* ≥ 3. Unpaired *t* test (**C**, **E**, and **F**); 1-way ANOVA with Dunnett’s correction (**A**, **B**, and **D**). *P < 0.05, **P < 0.01, ***P < 0.001, ****P < 0.0001.

To further interrogate the role for Ser256 phosphorylation in urate-induced AQP2 trafficking, we examined the nonphosphorylatable AQP2 mutant, Ser256Ala. Despite failure to respond to dDAVP, AQP2 Ser256Ala showed robust urate-induced apical accumulation comparable to wild-type AQP2 (Figure 7, C and D). PKA inhibition with PKI 14-22 blocked dDAVP-induced AQP2 trafficking but not the urate response (Figure 7, C and D), confirming PKA independence. Indeed, urate reduced intracellular PKA activity (~30% suppression at 500 μM; Figure 8A) and decreased cAMP in a concentration-dependent manner in both mIMCD3 and MDCK cells (Figure 8, B and C), in the absence or presence of forskolin or ABCG2 inhibition (Figure 8, D–F).

These findings establish that urate-induced AQP2 apical accumulation occurs independently of Ser256 phosphorylation and canonical cAMP/PKA signaling and is associated with suppression of intracellular cAMP levels and PKA activity.

### PDE4 mediates urate-induced AQP2 trafficking through coordinated cAMP degradation, AMP accumulation, and AMPK activation.

**Figure 9:**
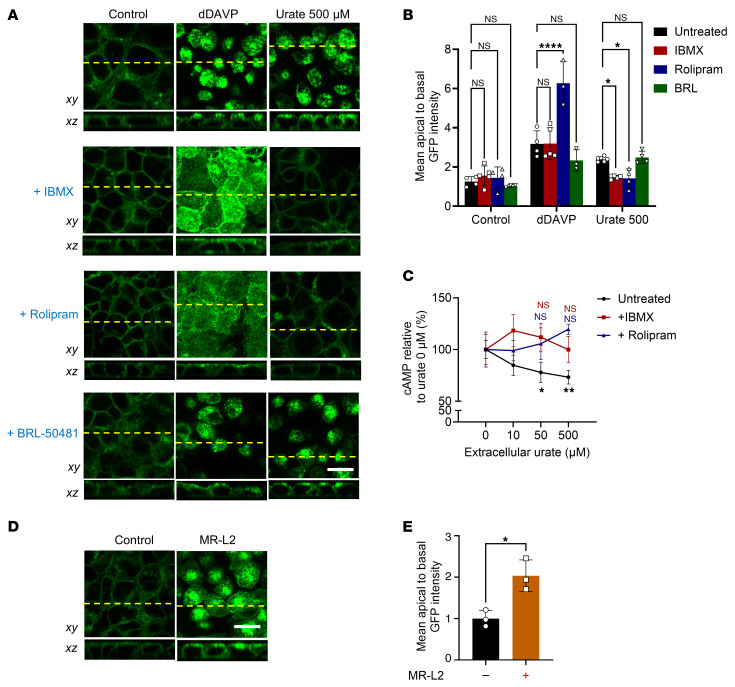
Urate-induced AQP2 trafficking is mediated by PDE4 activation. (**A**) Confocal images of AQP2-GFP localization in mIMCD3 cells. (**B**) Normalized apical-to-basal AQP2-GFP FI ratios showing that urate-induced (500 μM, 1 hour) AQP2 accumulation was blocked by pretreatment with IBMX (50 μM, 30 minutes) or rolipram (1 μM) but not by BRL-50481 (1 μM). (**C**) Normalized intracellular cAMP levels versus urate concentration ± IBMX or rolipram. (**D**) Confocal images of AQP2-GFP localization following PDE4 activation. (**E**) Normalized apical-to-basal AQP2-GFP FI ratios showing that MR-L2 (50 μM, 1 hour) induced apical AQP2 accumulation. Data are mean ± SD; *n* ≥ 3. Unpaired *t* test (**E**); 2-way ANOVA with Dunnett’s correction (**B** and **C**). *P < 0.05, **P < 0.01, ****P < 0.0001. Scale bar, 25 μm.

As urate suppresses intracellular cAMP, we next examined whether this effect is mediated by selective engagement of phosphodiesterase (PDE) isoforms that regulate cyclic nucleotide turnover. Urate-induced AQP2 accumulation was eliminated by broad-spectrum PDE inhibitor IBMX (50 μM) and by PDE4 inhibitor rolipram (1 μM) but not by PDE7 inhibitor BRL-50481 (1 μM) (Figure 9, A and B). PDE4 inhibition restored cAMP levels during urate exposure (Figure 9C), indicating accelerated cAMP hydrolysis. The PDE4 activator MR-L2 (50 μM) recapitulated urate’s effect on apical AQP2 accumulation (Figure 9, D and E).

**Figure 10:**
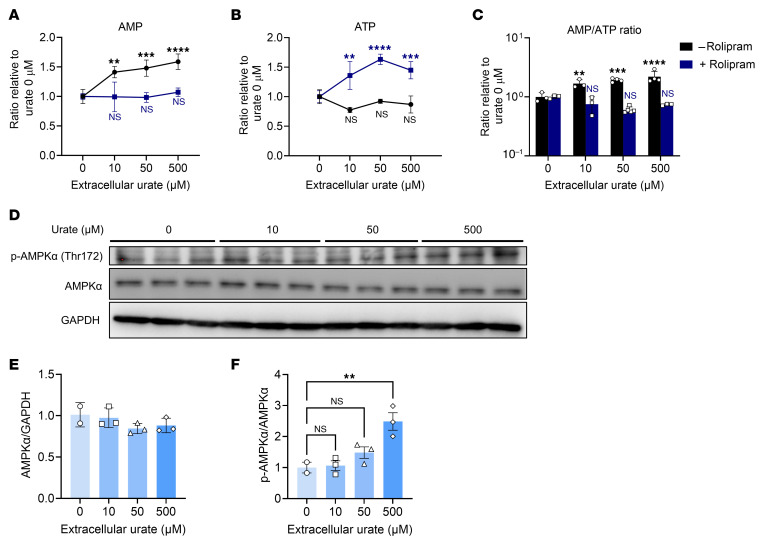
Urate-induced AQP2 trafficking is mediated by cAMP degradation, AMP accumulation, and AMPK phosphorylation. (**A** and **B**) Normalized AMP (**A**) and ATP (**B**) levels showing that rolipram pretreatment (30 minutes) blocked urate-induced AMP accumulation without affecting ATP. (**C**) AMP/ATP ratios. (**D**) Immunoblot of total AMPKα and phospho-AMPKα (Thr172) after 1-hour urate exposure. (**E** and **F**) Densitometric quantitation revealing urate concentration–dependent increase in phospho- but not total AMPKα. Urate treatments were in FBS-free medium. Data are means ± SD; *n* ≥ 3. Two-way ANOVA with Dunnett’s correction (**A**–**C**); 1-way ANOVA with Dunnett’s correction (**E** and **F**). **P < 0.01, ***P < 0.001, ****P < 0.0001.

As PDE4-mediated hydrolysis of cAMP generates AMP, we next assessed intracellular AMP levels. Urate increased intracellular AMP levels in a concentration-dependent manner, with a 40% rise detectable at 50 μM urate, accompanied by an elevated AMP-to-ATP ratio, effects prevented by PDE4 inhibition (Figure 10, A–C). We next evaluated downstream activation of energy-sensing pathways. Urate robustly increased phosphorylation of AMPK at Thr172, doubling the p-AMPK/AMPK ratio within 1 hour (Figure 10, D–F). A similar pattern of AMPK activation followed prolonged ABCG2 inhibition in GLUT9-deficient cells for 12–24 hours, a condition that gradually elevated endogenous intracellular urate, associated with delayed apical accumulation of AQP2 (Supplemental Figure 7, A and B). These data define a cascade in which intracellular urate engages PDE4 to promote cAMP degradation, AMP accumulation, and AMPK activation, culminating in apical AQP2 accumulation.

### Urate activates AMPK to regulate postendocytic AQP2 trafficking.

**Figure 11:**
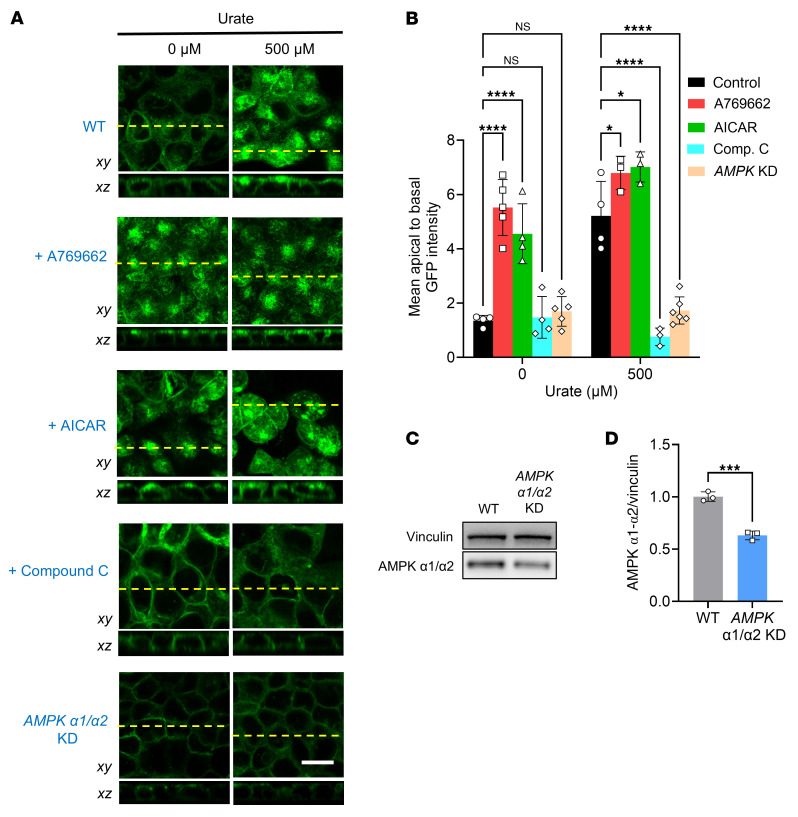
Urate-induced apical accumulation of AQP2 requires AMPK activation. (**A**) Confocal images of mIMCD3 AQP2-GFP cells. (**B**) Normalized apical-to-basal AQP2-GFP FI ratios showing that AMPK activators A769662 (5 μM) and AICAR (1 mM) induce apical AQP2 accumulation (1 hour), while urate-induced trafficking is abolished by AMPK inhibition (compound C, 10 μM) or *AMPKα1/α2* double knockdown (KD). (**C**) Immunoblot of AMPK α1/α2 in wild-type and *AMPK α1/α2*-KD cells. (**D**) Densitometric quantitation as in **C**. Urate treatments were in FBS-free medium. Data are means ± SD; *n* ≥ 3. Unpaired *t* test (**D**); 2-way ANOVA with Dunnett’s correction (**B**). *P < 0.05, ***P < 0.001, ****P < 0.0001. Scale bar, 25 μm.

To determine whether AMPK is required for urate-driven AQP2 trafficking to the apical membrane, we inhibited AMPK pharmacologically. AMPK inhibition with compound C (10 μM) abolished urate-induced AQP2 accumulation, whereas AMPK activation with either A769662 (5 μM) or AICAR (1 mM) was sufficient to induce it (Figure 11, A and B). Dual knockdown of *AMPK* catalytic subunits α1 and α2 similarly eliminated the urate response (Figure 11, A–D), confirming necessity and sufficiency of AMPK activity for urate-mediated AQP2 trafficking.

**Figure 12:**
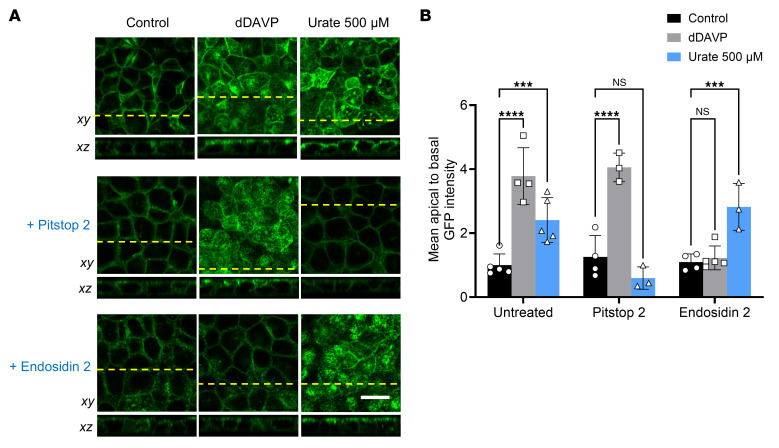
Urate-induced apical accumulation of AQP2 requires endosomal trafficking. (**A**) Confocal images of AQP2-GFP. (**B**) Normalized apical-to-basal AQP2-GFP FI ratios showing that endocytosis inhibitor pitstop2 (5 μM) blocks urate-induced AQP2 trafficking, whereas exocytosis inhibitor endosidin2 (50 μM) has no effect. Cells were pretreated 30 minutes with inhibitors before 1-hour stimulation with dDAVP (100 nM) or urate (500 μM). Urate treatments were in FBS-free medium. Data are means ± SD; *n* ≥ 3. Two-way ANOVA with Dunnett’s correction (**B**). ***P < 0.001, ****P < 0.0001. Scale bar, 25 μm.

We next investigated the membrane-trafficking components through which urate acts. Endocytosis inhibitor pitstop2 blocked urate-induced AQP2 accumulation, whereas exocytosis inhibitor endosidin2 had no effect. In contrast, only endosidin2, but not pitstop2, abolished dDAVP-induced AQP2 trafficking (Figure 12, A and B). This reciprocal pharmacological sensitivity suggests that urate-induced AQP2 apical accumulation is mechanistically distinct from the vasopressin-induced trafficking pathway(s), instead requiring ongoing endocytosis to mobilize intracellular AQP2 pools.

**Figure 13:**
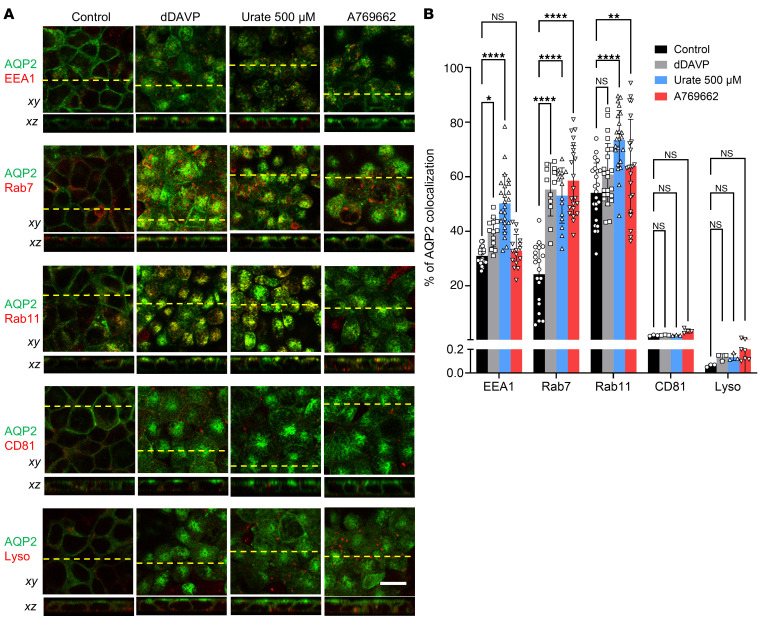
Urate-induced AQP2 trafficking requires AMPK activation and endosomal trafficking. (**A**) Confocal images of AQP2-GFP after 1-hour treatment with dDAVP (100 nM), apical urate (500 μM), or A769662 (5 μM), costained with intracellular trafficking markers EEA1 (early endosomes), Rab7 (late endosomes), Rab11 (recycling endosomes), CD81 (exosomes), and LysoTracker (lysosomes), showing AQP2-GFP enrichment in Rab7 and Rab11 compartments. (**B**) AQP2 colocalization (%) with indicated markers ± dDAVP, urate, or A769662. Urate treatments were in FBS-free medium. Data are means ± SD; *n* ≥ 3. Two-way ANOVA with Dunnett’s correction (**B**). *P < 0.05, **P < 0.01, ****P < 0.0001. Scale bar, 25 μm.

**Figure 14:**
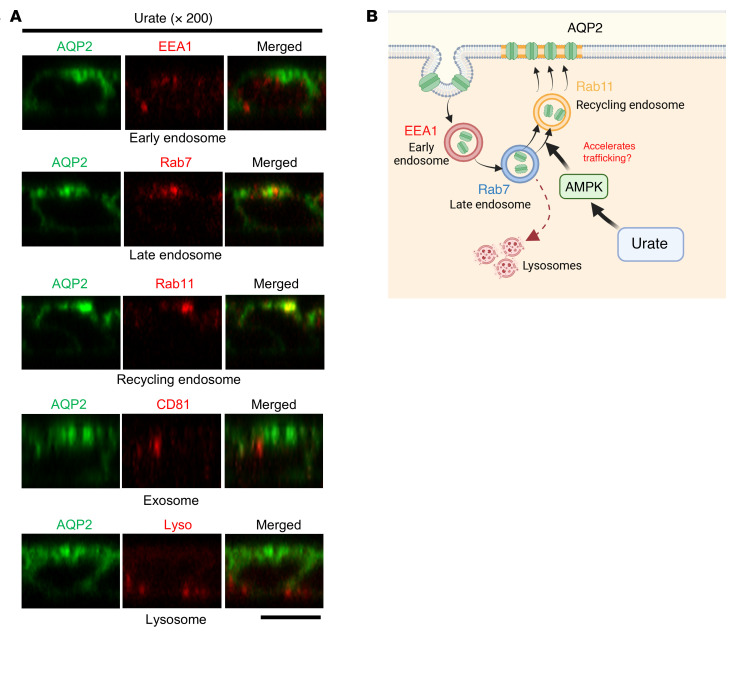
Proposed mechanism of urate-induced AQP2 trafficking. (**A**) Original magnification, ×200, of XZ sections corresponding to yellow dashed lines in Figure 13A. (**B**) Schematic of proposed mechanism of urate- and AMPK-dependent endosomal trafficking of AQP2. All urate treatments were performed in FBS-free medium. Scale bar, 15 μm.

To define postendocytic trafficking routes engaged by urate, we quantified AQP2-GFP colocalization with markers of early endosomes (EEA1), late endosomes (Rab7), recycling endosomes (Rab11), lysosomes (LysoTracker), and exosomes (CD81). Extracellular urate increased AQP2-GFP colocalization with EEA1, Rab7, and most prominently, Rab11, consistent with enhanced trafficking through late and recycling endosomal compartments. In contrast, colocalization with lysosomal or CD81-positive exosomal compartments was minimal (Figure 13, A and B, and Figure 14A). AMPK activation (A769662) recapitulated the urate-induced increase in AQP2-GFP colocalization with Rab7 and Rab11, without affecting EEA1, CD81, or LysoTracker (Figure 13, A and B, and Figure 14A), linking AMPK to selective routing of AQP2 through late and recycling pathways. To define trafficking requirements underlying urate-induced AQP2 redistribution, we disrupted vesicular transport using complementary approaches. Pretreatment with colchicine (10 μM), which depolymerizes microtubules and impairs long-range vesicular transport between endosomal compartments and the apical membrane (44, 45), completely abolished apical AQP2 accumulation induced by urate or dDAVP (Supplemental Figure 7, C and D). Similarly, acute cold exposure (15 minutes at 4**°**C), which suppresses membrane trafficking and endosomal recycling, eliminated urate- and dDAVP-induced AQP2 translocation (Supplemental Figure 7, C and D). Together, these interventions indicate that urate, like vasopressin, relies on microtubule-dependent postendocytic trafficking to achieve apical AQP2 accumulation.

These findings support a model in which intracellular urate, acting through AMPK, directs AQP2 toward Rab7-positive late endosomes and Rab11-positive recycling compartments, potentially accelerating trafficking of internalized AQP2 to the apical membrane (Figure 14B). This process depends on intact endocytosis and is not inhibited by disruption of exocyst-dependent vesicle insertion under the conditions tested, as nominal disruption of endocytosis, but not of exocytosis, abolished the urate response. Thus, urate appears not to enhance regulated exocyst-dependent exocytic delivery but rather regulates postendocytic trafficking of preexisting AQP2 pools, by apparent acceleration of their exit from intracellular endosomal compartments to the apical plasma membrane.

### Probenecid reduces tolvaptan-induced polyuria while preserving therapeutic efficacy in ADPKD mice.

**Figure 15:**
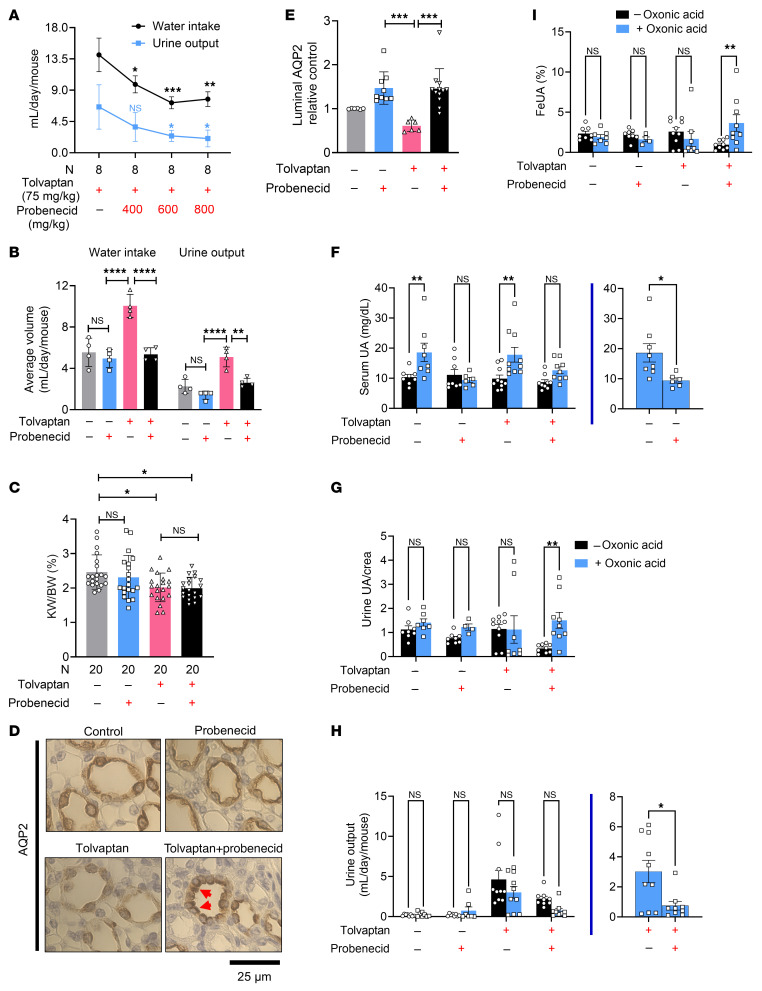
Probenecid attenuates tolvaptan-induced polyuria in mice. (**A**) The 24-hour water intake and urine output of wild-type mice gavaged with tolvaptan (75 mg/kg) ± probenecid (400, 600, and 800 mg/kg). (**B**) *Pkd1^RC/RC^* mice treated with tolvaptan (0.3%) and probenecid (0.4%) in chow from 4–16 weeks exhibited reduced 24-hour water intake and urine output (5 mice/cage analyzed as single value). (**C**) Kidney weight-to-body weight ratio (KW/BW, %) in *Pkd1^RC/RC^* mice as in **B**, showing that probenecid did not impair tolvaptan’s anticyst effect. (**D** and **E**) Quantitative immunohistochemistry showing increased apical AQP2 localization (red arrowheads) in inner medullary collecting ducts of probenecid-treated *Pkd1^RC/RC^* mice. (**F**) Wild-type mice treated for 3 days with tolvaptan (75 mg/kg), probenecid (600 mg/kg), or both, ± oxonic acid (300 mg/kg). All treatment (left) and oxonic acid-treated mice treated ± probenecid (right). Oxonic acid–increased serum uric acid (UA) was prevented by probenecid. (**G** and **I**) In tolvaptan-treated mice, probenecid unmasked oxonic acid–induced increases in urinary urate-to-creatinine ratio (UA/Crea) and fractional excretion of uric acid (FeUA). (**H**) Marked tolvaptan-induced polyuria and polydipsia (not shown) were attenuated by probenecid ± oxonic acid across all treatment (left) and oxonic acid–treated mice treated with tolvaptan ± probenecid (right). Data are means ± SD (**B**–**E**) or SEM (**F**–**I**); *n* = 8 mice/group (**F**–**I**) or *n* = 10/sex/group (**B**–**E**). In panels **F**–**I**, black bars indicate no oxonic acid, and blue bars indicate oxonic acid treatment. Two-way ANOVA with Bonferroni’s correction (**B**–**I**) or Dunnett’s correction (**A**). *P < 0.05, **P < 0.01, ***P < 0.001, ****P < 0.0001.

To determine whether probenecid attenuates the aquaretic effects of tolvaptan, we first quantified water intake and urine output in tolvaptan-gavaged wild-type mice. Tolvaptan alone induced marked polydipsia and polyuria, with mean water intake of 14 mL/d and urine output of 6.6 mL/d (Figure 15A). Probenecid coadministration produced dose-dependent reductions in both parameters. At 0.2% probenecid, water intake per mouse decreased 30% to 9.8 mL/d, and urine output decreased 43% to 3.7 mL/d versus tolvaptan alone. At 0.3% probenecid water intake decreased 49% to 7.1 mL/d, and urine output decreased 63% to 2.4 mL/d. Similar effects were observed with 0.4% probenecid, with ~7.7 mL/d water intake and urine output reduced 68% to 2 mL/d (Figure 15A).

We next examined whether probenecid mitigates chronic tolvaptan-induced polyuria in the orthologous *Pkd1*^RC/RC^ mouse model of ADPKD. Mice were treated from 4 to 16 weeks of age with tolvaptan (0.3%), probenecid (0.4%), or the combination. Relative to tolvaptan alone, combination therapy lowered daily water intake from 10.1 ± 1.1 to 5.4 ± 0.6 mL/d (*P* < 0.01) and reduced urine output from 5.1 ± 1.0 to 2.6 ± 0.4 mL/d (*P* < 0.01; Figure 15B).

To test whether probenecid interferes with tolvaptan’s disease-modifying effect, we measured kidney weight-to-body weight ratio (KW/BW) in *Pkd1*^RC/RC^ mice. Tolvaptan reduced KW/BW relative to untreated controls (*P* < 0.01), an effect preserved with probenecid cotreatment (Figure 15C). Furthermore, probenecid improved total kidney volume progression and reduced plasma creatinine when combined with tolvaptan (Supplemental Figure 8, A–C). Consistent with enhanced collecting duct water reabsorption, probenecid also restored the apical AQP2 expression suppressed by tolvaptan (Figure 15, D and E).

Thus, probenecid markedly attenuates tolvaptan-induced polyuria while preserving tolvaptan’s efficacy in limiting cyst growth, supporting a potential strategy to improve tolerability of chronic vasopressin antagonism in ADPKD.

### Probenecid retains its antiaquaretic effect in a murine model that mimics human urate metabolism.

To determine whether probenecid’s antiaquaretic effect persists under conditions approximating human urate physiology, we inhibited endogenous murine uricase with oxonic acid to “humanize” systemic and nephron luminal urate levels. Wild-type mice were treated for 3 days with oxonic acid (300 mg/kg), tolvaptan (75 mg/kg), and probenecid (600 mg/kg), alone or in combination (*n* = 8 per group).

Oxonic acid increased serum urate concentrations, confirming effective uricase inhibition (Figure 15F). Probenecid prevented the uricase-induced rise in serum urate (Figure 15F). In tolvaptan-treated mice, probenecid unmasked a uricosuric response under uricase inhibition, reflected by increased urine urate-to-creatinine ratios and higher fractional excretion of uric acid (FeUA) (Figure 15, G and I). The aquaretic response to tolvaptan was independent of uricase inhibition (Figure 15H). Importantly, probenecid continued to suppress tolvaptan-induced polyuria in oxonic acid–treated mice (Figure 15H). Collectively, these findings demonstrate retention of probenecid’s antiaquaretic effect in a uricase-deficient state approximating human urate biology.

### Probenecid reduces tolvaptan-induced polyuria and improves aquaresis-related symptoms in patients with ADPKD.

**Figure 16:**
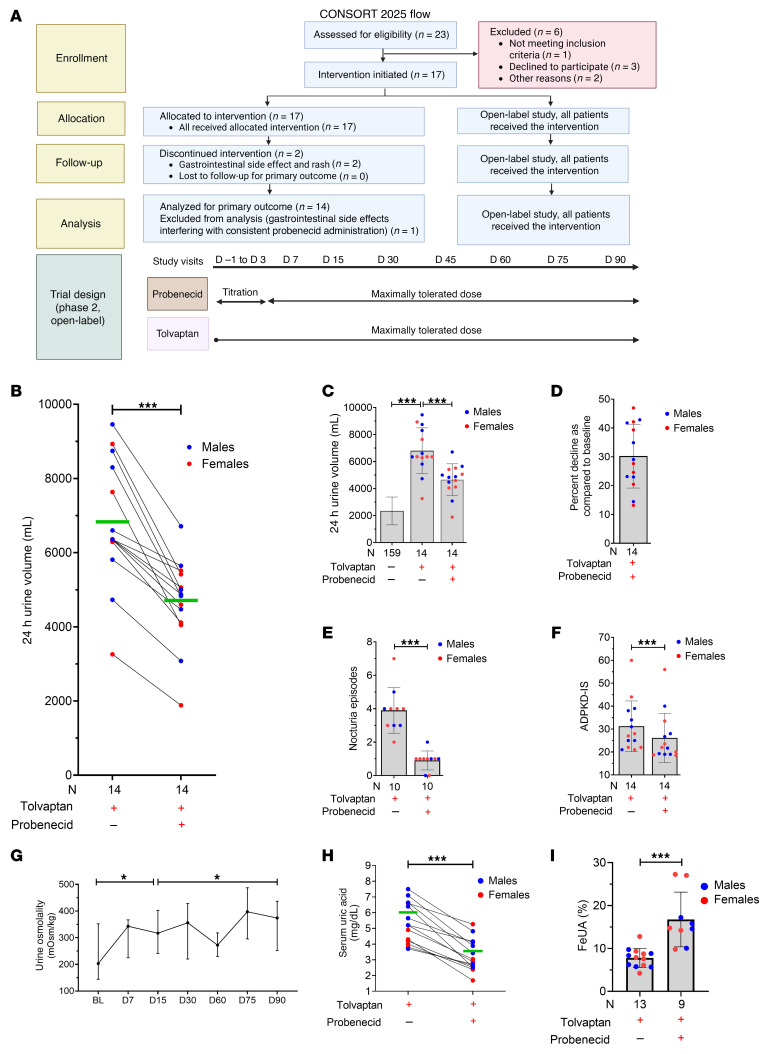
Probenecid attenuates tolvaptan-induced polyuria and improves aquaresis symptoms in individuals with ADPKD. (**A**) Schematic of the phase II, open-label SereNDIpity-pb1 trial. Seventeen individuals with ADPKD on stable tolvaptan were enrolled at Mayo Clinic Florida and Hôpital du Sacré-Cœur and underwent probenecid dose escalation to maximally tolerated doses, with assessment at baseline (BL) and days 7, 15, 30, 45, 60, 75, and 90. (**B**) Dot plot of 24-hour urine volume (*n* = 17), comparing baseline (tolvaptan alone) and ≥7 days of probenecid; lines connect paired samples (blue, males; red, females). (**C**) Mean 24-hour urine volume (± SD) stratified by sex and compared with a historical ADPKD cohort without tolvaptan or probenecid (*n* = 159). (**D**) Probenecid-induced reduction in 24-hour urine volume (%) relative to baseline. (**E**) Nocturia episodes before and after probenecid, stratified by sex. (**F**) ADPKD Impact Scale (ADPKD-IS) scores before and after treatment. (**G**) Mean 24-hour urine osmolality over 90 days’ treatment. (**H**) Paired serum UA concentrations before and after ≥7 days of probenecid. (**I**) FeUA (%) stratified by sex. Statistical analyses: paired *t* test (**E**, **F**, and **I**), 1-way ANOVA with Bonferroni’s correction (**C**), and independent 2-sample *t* tests for continuous variables and χ^2^ or Fisher’s tests for categorical variables. *P < 0.05, ***P < 0.001.

The phase II, open-label SereNDIpity trial enrolled 17 participants with ADPKD receiving stable tolvaptan therapy. Probenecid was initiated using a dose-escalation protocol starting at 500 mg twice daily and increasing to 1,000 mg twice daily, followed by treatment at the maximally tolerated dose for up to 90 days (Figure 16A). Participants’ mean age was 46.6 ± 10.5 years, 52.9% were female, and mean estimated glomerular filtration rate (eGFR) was 66.6 ± 22.5 mL/min/1.73 m^2^. 88.2% were hypertensive, and 47.1% had chronic kidney disease stage 3 (Supplemental Table 1). A historical comparator group of 159 individuals with ADPKD not receiving tolvaptan or probenecid and matched for age, sex, eGFR, and Mayo imaging classification subclasses 1C, 1D, or 1E served as a reference population (Supplemental Table 1).

Baseline 24-hour urine volumes were markedly elevated in the tolvaptan-treated cohort (6,798 ± 1,693 mL/d), consistent with chronic aquaresis and substantially exceeding volumes observed in historical controls (2,343 ± 1,031 mL/d; Figure 16C). After at least 7 days’ probenecid therapy, mean urine output decreased by 30.2% ± 11.1% to 4,662 ± 1,168 mL/d (*P* < 0.0001; Figure 16, B–D). Nighttime voiding frequency decreased from 3.9 ± 1.4 to 0.9 ± 0.6 episodes/night (*P* = 0.0004; Figure 16E). Symptomatic improvements were reflected by ADPKD-Impact Scale scores being reduced from 32.4 ± 11.6 to 27.4 ± 11.4 (*P* < 0.0001; Figure 16F). Morning urine osmolality increased from 248 ± 126 to 324 ± 85 mOsm/kg (*P* = 0.017; Figure 16G).

As expected for a uricosuric agent, serum uric acid decreased from 5.4 ± 1.3 to 3.2 ± 1.1 mg/dL (*P* < 0.0001; Figure 16H). Serum uric acid fell significantly by day 7 and remained suppressed through day 90 (*P* < 0.001; Supplemental Figure 9C), accompanied by increased FeUA from 7.7% during tolvaptan therapy alone to 16.7% after added probenecid (Figure 16I). This uricosuric response parallels probenecid effects observed in tolvaptan-treated mice with uricase inhibition (Figure 15H). Mean eGFR declined from a baseline of 71.9 ± 21.1 to 63.9 ± 19.8 mL/min/1.73 m^2^ during probenecid treatment (*P* = 0.003) and returned to baseline after probenecid discontinuation (70.4 ± 17.7 mL/min/1.73 m^2^, *P* = 0.038; Supplemental Table 1 and Supplemental Figure 9A). This reversible eGFR decrease suggests probenecid-mediated inhibition of tubular creatinine secretion through organic anion transporters rather than glomerular impairment (46). Serum sodium remained stable throughout (Supplemental Table 1), and liver enzymes remained within normal range (*P* > 0.29) (Supplemental Figure 9B). The most common adverse effects were gastrointestinal symptoms (35.2%), rash (23.5%), and headache (17.6%) (Supplemental Table 1), and no serious adverse events occurred. Circulating levels of the stable vasopressin surrogate, copeptin, were indistinguishable in the tolvaptan-alone and combination therapy groups (Supplemental Figure 9D).

## Discussion

Our study identifies intracellular urate as an endogenous regulator of collecting duct water transport and delineates a transport-coupled signaling axis in which the balance between apical urate influx through GLUT9b and efflux through ABCG2 determines steady-state intracellular urate levels. Intracellular urate engages a PDE4-dependent cascade that lowers cAMP, increases AMP, activates AMPK, and increases apical AQP2 localization. Pharmacological inhibition of ABCG2 with probenecid attenuates tolvaptan-induced aquaresis in wild-type mice, in orthologous *Pkd1*^RC/RC^ ADPKD mice, and in individuals with ADPKD receiving tolvaptan. These findings position intracellular urate as a metabolic signal that modulates AQP2-dependent water reabsorption through a vasopressin-independent pathway and suggest a strategy to mitigate aquaretic effects of V2R antagonism in ADPKD.

These observations extend current models of AQP2 regulation in collecting duct principal cells. Canonical water reabsorption is governed by vasopressin-dependent activation of V2 receptors, cAMP generation, and PKA-mediated phosphorylation of AQP2, integrated with endocytosis, recycling, and exocytosis to determine apical AQP2 abundance (8). AQP2 surface levels are now understood to reflect a dynamic equilibrium among insertion, retrieval, recycling, and degradation within spatially restricted signaling microdomains (9, 47–50). Within this framework, urate appears to function as a noncanonical, vasopressin-independent signal that influences postendocytic trafficking processes in principal cells.

Urate promotes AQP2 accumulation despite suppression of cAMP/PKA signaling. Urate reduced intracellular cAMP even in the presence of forskolin, consistent with enhanced cAMP hydrolysis. PDE4 emerged as a key mediator, as its inhibition prevented urate-induced AQP2 accumulation and restored cAMP, whereas its activation recapitulated the urate response. PDE4-mediated cAMP hydrolysis was associated with increased AMP generation, an elevated AMP-to-ATP ratio, and AMPK activation, indicating coupling between cyclic nucleotide turnover and cellular energy sensing. These findings support a model in which urate shifts signaling from a PKA-dominant to an AMPK activation–associated state, with downstream consequences for AQP2 distribution (15, 16).

The trafficking features of this pathway differ from canonical vasopressin signaling but remain incompletely defined. Urate-induced apical AQP2 accumulation likely depends on ongoing endocytosis, as inhibition with pitstop2 abolished the response. In contrast, putative inhibition of exocyst-dependent exocytic vesicle insertion by endosidin2 suppressed dDAVP-induced trafficking but did not measurably affect the urate response under the tested conditions. These observations support the proposal that urate accelerates mobilization of internalized AQP2 pools to the apical membrane and are consistent with our observation of urate-increased AQP2 colocalization with Rab7-positive late endosomes and Rab11-positive recycling compartments, without evidence of increased lysosomal or exosomal targeting. These findings do not exclude continued constitutive exocytic delivery and endocytic retrieval. The relative contributions of endocytosis, recycling, and exocytosis to the net increase in apical AQP2 remain to be resolved. Important considerations in interpreting these findings are the magnitude and kinetics of AQP2 accumulation observed over the experimental time frame. Most internalized AQP2 is thought to reenter recycling pathways, with only a minor fraction targeted for degradation. Moreover, AQP2 has a relatively long half-life in principal cells (6–14 hours) (51, 52), exceeding the rapid urate response (≤1 hour). Accordingly, diversion from lysosomal pathways alone is unlikely to fully explain the observed increase in apical abundance. A more plausible interpretation is that urate enhances trafficking efficiency within existing recycling pathways, potentially by modulating recycling kinetics, vesicle delivery, and secondarily, AQP2 cell membrane residence time and activity. Defining the specific trafficking step(s) affected by urate will require quantitative, time-resolved approaches. Live-cell imaging, pulse-chase analysis, and complementary approaches such as fluorescence recovery after photobleaching (FRAP) or single-particle tracking could distinguish effects on recycling flux, vesicle fusion probabilities, and stability of membrane residency. In parallel, genetic perturbation of defined trafficking machinery will be necessary to establish causality and move beyond association toward a mechanistic understanding of urate-dependent AQP2 regulation.

Within this signaling framework, PDE4 functions as a nodal regulator linking intracellular urate to AQP2 trafficking. PDE4 is highly expressed in collecting duct principal cells and shapes compartmentalized cAMP microdomains. Whereas prior therapeutic efforts in nephrogenic diabetes insipidus have focused on increasing cAMP through PDE inhibition (53–55), the present findings support a distinct paradigm in which PDE4 activation lowers cAMP while generating AMP and engaging AMPK-dependent signaling. The mechanism by which urate engages PDE4 remains undefined and may involve direct effects on enzyme activity or indirect modulation of localized signaling complexes. Resolving these possibilities will require integration of structural modeling, biochemical validation, and compartment-specific measurements of cAMP dynamics. Although the present work focuses on PDE4 based on its localized expression, functional relevance, and tractability, other collecting duct–expressed PDEs may similarly shape cAMP microdomains and warrant investigation of possible contributions toward the AMP elevation that engages AMPK-dependent, PKA-independent vesicular trafficking and apical accumulation of AQP2 (56–58).

AMPK emerges as a key downstream effector of urate. Pharmacologic AMPK activation has been shown to increase apical AQP2 abundance and improve water reabsorption in vasopressin-resistant states (21, 22). In this context, the urate/PDE4 axis provides a mechanism to generate AMPK-activating signals from cyclic nucleotide turnover, linking metabolic state to water transport independently of vasopressin signaling.

Another conceptual advance is the implication of GLUT9b and ABCG2 as collecting duct regulators of intracellular urate signaling. Although renal urate handling has classically been attributed to the proximal tubule (29–31, 59), the apical localization of these transporters in principal cells supports a role for distal nephron urate handling. Our data indicate that intracellular urate levels reflect the balance between GLUT9b-mediated influx and ABCG2-mediated efflux and that perturbation of either is sufficient to alter AQP2 localization. This framework provides a mechanistic context for prior observations linking urate handling to disorders of water balance. Loss-of-function human mutations in *GLUT9* cause hypouricemia with hyperuricosuria and are classically associated with exercise-induced acute kidney injury (34, 45). Although this syndrome is likely multifactorial, our findings raise the possibility that impaired urate entry into collecting duct principal cells limits engagement of urate-responsive antidiuretic signaling, increasing susceptibility to kidney injury during physiological stress. Global or kidney-specific *Glut9* deletion in mice produces marked polyuria and impaired urinary concentrating ability, despite intact vasopressin signaling and corticomedullary gradient formation, a phenotype unexplained by canonical V2R pathways (33–35). These observations support a role for distal urate handling in urinary concentration.

Our findings generate testable hypotheses for disorders of water balance. SIAD is accompanied by hypouricemia and increased FeUA (36, 37). Enhanced distal urate delivery could elevate intracellular urate concentration in collecting duct principal cells to promote vasopressin-independent AQP2 accumulation, possibly supplementing AVP-induced water retention. Reported decreases in GLUT9b and reciprocal increases in ABCG2 in experimental SIAD (37) may represent compensatory responses to restrain intracellular urate signaling. Whether this response in SIAD serves to limit the antidiuresis warrants further investigation. Moreover, chronic hyperuricemia reduces expression of AQP2, AQP3, and AQP4 through NF-κB–dependent transcriptional mechanisms (60). In contrast, our findings define acute, cell-intrinsic effects of intracellular urate on AQP2 trafficking without apparent change in total protein abundance. These observations suggest urate regulation of collecting duct water handling at multiple levels: hyperuricemia may reduce aquaporin expression, whereas the extent of collecting duct urate uptake and intracellular concentrations modulate the localization and availability of existing channels.

Effective urate concentrations and cross-species considerations support physiological relevance of our findings. Urate concentrations effective in vitro overlap with those observed in vivo (40, 41). In addition, probenecid retained efficacy under uricase inhibition, approximating human physiology with low millomolar urinary urate concentrations (61, 62). Rigorous validation confirmed specificity of intracellular urate measurements. Urate-dependent AQP2 regulation was independent of UT-A–mediated urea transport, indicating that this pathway operates in parallel with classical concentrating mechanisms.

From a translational perspective, ABCG2-mediated urate efflux represents a modifiable checkpoint in the regulation of water balance. Probenecid attenuated tolvaptan-induced aquaresis while preserving its effect on cyst growth, indicating that aquaresis can be modulated independently of tolvaptan’s disease-modifying efficacy. This uncoupling of water handling from cystogenic signaling is consistent with compartmentalization of cAMP-dependent pathways and supports feasibility of targeting urate’s downstream modulators of fluid transport without compromising probenecid’s therapeutic benefit. These findings provide a rationale for developing more selective urate transport modulators to reduce polyuria in congenital nephrogenic diabetes insipidus and to improve tolerability and adherence to vasopressin antagonist therapy in ADPKD, potentially as part of combination strategies targeting complementary pathways. Aquaretic symptoms are major drivers of tolvaptan dose reduction and treatment discontinuation (63–66). In this context, our clinical trial data show modulation of urate transport reduced urine volume and nocturia, improved patient-reported symptom burden, and increased morning urine osmolality. However, increased uricosuria raises safety considerations, including lithogenic risk. This underscores the need for longer term studies using more selective urate transport modulators.

Against this backdrop, we tested whether pharmacologic inhibition of ABCG2 might slow cyst progression. Probenecid monotherapy did not reduce KW/BW in the *Pkd1*^RC/RC^ model. Several non–mutually exclusive explanations may account for this. First, probenecid is a low-affinity, nonselective ABCG2 inhibitor, and the increased intracellular urate concentration achieved in vivo may be insufficient to suppress cystogenic cAMP signaling, suggesting that more potent or selective ABCG2 inhibitors, or direct activation of GLUT9b to enhance urate influx, may be required for disease modification. Second, urate-dependent cAMP suppression may preferentially operate within microdomains regulating AQP2 trafficking rather than epithelial proliferation. Third, probenecid exerts broad systemic effects through inhibition of multiple organic anion transporters, which may partially offset anticyst benefit while preserving antiaquaretic efficacy. However, the lack of cyst burden reduction by probenecid monotherapy does not diminish the biological or therapeutic relevance of the urate/ABCG2/PDE4 axis. Rather, it emphasizes that modulation of vasopressin-independent cAMP signaling can improve water handling without adversely affecting disease control. In this context, restoration of apical AQP2 trafficking in the *Pkd1*^RC/RC^ model without detectable alteration of renal cystic index indicates that enhancing collecting duct water reabsorption can be achieved without accelerating cyst growth over the treatment interval examined.

Several limitations of our study warrant consideration. Mechanistic studies were performed in cultured cells, and the molecular interface linking urate to PDE4 activation remains undefined. Compartment-specific cyclic nucleotide signaling dynamics were inferred rather than directly measured. Our open-label clinical study of limited size should be considered hypothesis generating for larger trials with longer treatment duration.

Future studies should define the urate-responsive interface linking intracellular urate to PDE4 activation and delineate compartment-specific, AMPK-dependent signaling microdomains governing AQP2 trafficking. Approaches enabling segment-specific and temporally controlled genetic or pharmacologic manipulation of urate transport and signaling will be particularly informative. In parallel, interrogation of water balance phenotypes in individuals with GLUT9 loss-of-function variants will be essential to establish the contribution of this pathway to human disorders of water homeostasis and to directly assess its impact on cystogenesis.

In summary, our work identifies intracellular urate as a physiologic regulator of collecting duct water handling. We define a vasopressin-independent, PDE4/AMPK–dependent signaling mechanism through which urate transport and metabolism intersect with endocytic trafficking pathways to control apical AQP2 abundance in collecting duct principal cells. This transport-coupled urate signaling axis preserves water reabsorption without compromising tolvaptan’s suppression of cystogenic signaling, expanding current models of collecting duct regulation, offering a strategy to mitigate the aquaretic burden of V2 receptor antagonism in ADPKD, and positioning urate-sensitive signaling as a therapeutic target across the broader spectrum of disorders of urinary concentration.

## Methods

### Sex as a biological variable

Both male and female mice were included in in vivo studies. As experiments were not powered to detect sex-specific differences, analyses were performed with sexes combined. No consistent qualitative differences were observed. The human study also included both sexes. Findings are expected to be broadly applicable, though possible sex-specific effects require further study.

### Chemical compounds

All reagents were obtained from commercial suppliers: dDAVP (Merck V1005), tolvaptan (Merck T7455), forskolin (Merck F3917), uric acid (Sigma-Aldrich U2625), probenecid (Thermo Fisher Scientific P36400), rasburicase (MedChemExpress HY-108844), colchicine (Sigma-Aldrich C9754), urea (Sigma-Aldrich U5378), oxonic acid potassium salt (Selleckchem 2207-75-2), benzbromarone (Merck B5774), resveratrol (Merck R5010), febuxostat (Sigma-Aldrich SML1285), novobiocin (Selleckchem S2492), Ko143 (Selleckchem S7043), PKI 14-22 (Tocris 2546), dorsomorphin (compound C; Selleckchem S7840), A-769662 (Selleckchem S2697), AICAR (Selleckchem S1802), endosidin2 (MedChemExpress HY-120821), pitstop2 (Selleckchem S9670), IBMX (Selleckchem S5836), rolipram (Selleckchem S1430), BRL-50481 (Selleckchem S1430), and MR-L2 (MedChemExpress HY-128358).

### Cell lines, transfection, and siRNA knockdown

mIMCD3 and MDCK cells (ATCC) were cultured in DMEM/F12 or DMEM, respectively, supplemented with 5%–10% FBS and penicillin-streptomycin at 37°C in 5% CO_2_. Serum-free media were used for experiments requiring defined urate concentrations. Cells were seeded on 0.4 μm Transwell filters (Corning, 3460) or 6-well plates.

Stable AQP2-GFP or GLUT9b-overexpressing cell lines were generated by Lipofectamine 2000–mediated transfection (Invitrogen 11668019) using C-terminally GFP-tagged mouse *Aqp2* plasmid (OriGene MG224809), mouse *Aqp2* plasmid (OriGene MR224809), or mouse *Slc2a9b* plasmid (OriGene MR222806) followed by FACS selection and validation by Western blot.

siRNA-mediated knockdown (Santa Cruz Biotechnology) targeting mouse *V2r* (sc-40276), *Slc2a9/Glut9* (sc-145451), *Abcg2* (sc-37054), *Panx1* (sc-61288), *Nlrp3* (sc-45470), *Slc14a2/UT-A* (sc-45313), *Prkaa1/Prkaa2* (sc-154952), or scrambled control (sc-37007) was performed in AQP2-GFP mIMCD3 cells and analyzed 48 hours posttransfection.

### Cell viability and IC50 determination

Cell viability was assessed by CellTiter-Glo (Promega G7571). Cells plated in 96-well format were treated 1 hour in serum-free media or as indicated. Luminescence was measured by plate reader (CLARIOstar Plus, BMG Labtech). IC_50_ values were calculated using GraphPad Prism.

### RNA isolation and real-time qPCR

Total RNA was isolated by RNeasy Plus Mini Kit (QIAGEN 74104), reverse-transcribed (High-Capacity cDNA RT Kit; Invitrogen 4368814), and analyzed by SYBR Green qPCR (Applied Biosystems 4309155) on QuantStudio 3 (Thermo Fisher Scientific). Primers are listed in Supplemental Table 3A. Knockdown and overexpression efficiency were validated by qPCR, normalized to *Gapdh* (Supplemental Figure 7, E–N).

### Cell surface biotinylation

Cell surface proteins were labeled using Pierce Cell Surface Biotinylation Kit (Thermo Fisher Scientific 89881), precipitated with NeutrAvidin, and analyzed by Western blot.

### Immunofluorescence, confocal microscopy, and bright-field and polarized imaging

Cells cultured on Transwell membranes were treated as indicated for each experiment, washed 3 times with PBS 1×, fixed in paraformaldehyde (4% in PBS 1×) 10 minutes at room temperature (RT), permeabilized 5 minutes with Triton X-100 0.1% in PBS, blocked 1 hour with 5% BSA in PBS with 0.1% Tween, and immunostained using antibodies listed in Supplemental Table 4. Lysosomes were labeled with LysoTracker Deep Red (Thermo Fisher Scientific L12492). Nuclei were stained 10 minutes at RT with DAPI (1 μg/mL). Cells were mounted using VECTASHIELD (Vector Laboratories, H-1400-10). Images were acquired by ZEISS LSM700 confocal microscope. Apical AQP2 enrichment was quantified from XZ optical sections as the ratio of apical-to-basal AQP2-GFP FI within the same cell, normalized to control conditions. Uric acid crystallization was assessed using bright-field (Thermo Fisher Scientific EVOS M5000) and polarized light microscopy (AmeScope PZ200TA) following exposure to urate solutions (0, 500, or >5,000 μM) prepared in milliQ water (MilliporeSigma) (pH 7) or serum-free DMEM/F12 (pH 7.5), then incubated at 37°C with continuous agitation 1 hour prior to imaging. Solutions were applied on cells and imaged under crossed polarized light and by bright-field microscopy. Crystal birefringence was quantified using ImageJ (NIH).

### Immunoblots

Cells were lysed in RIPA buffer containing protease and phosphatase inhibitors (Thermo Fisher Scientific 78444). Proteins were quantified by Pierce BCA assay (Thermo Fisher Scientific 23225), fractionated and resolved by SDS-PAGE, blocked with skim milk or BSA (0.5% in TBS-Tween 0.1%), probed with antibodies (Supplemental Table 4), and developed using Pierce ECL Western Blotting Substrate (Thermo Fisher Scientific 32209) or SuperSignal West Dura substrate (Thermo Fisher Scientific 34076). Protein bands were imaged by Bio-Rad Chemidoc MP and quantified using ImageJ.

### Site-directed mutagenesis

The AQP2 S256A mutant was generated by PCR mutagenesis of mouse GFP-tagged mouse *Aqp2* plasmid (OriGene MG224809) using Platinum SuperFi II polymerase (Thermo Fisher Scientific 12368010) and primers in Supplemental Table 3B, cloned in *E*. *coli* DH5α (Zymo T3007), and verified by DNA sequencing.

### PKA and cAMP assays

Cells were cultured in 6-well plates to 90%–95% confluence and treated with urate as indicated in serum-free media. PKA activity was measured by ELISA (Thermo Fisher Scientific EIAPKA) following cell lysis under kinase-preserving conditions. Intracellular cAMP levels were quantified after acid extraction by ELISA (Enzo ADI-900-066A) and normalized to total protein (quantified by BCA assay) using CLARIOstar Plus.

### Metabolite quantitation

Intracellular urate was quantified fluorometrically using Uric Acid Assay Kit (Abcam ab65344) following cell lysis and clarification. AMP and ATP were measured using colorimetric (Abcam ab273275) and luminescence assays (Promega CellTiter-Glo). Metabolite levels were calculated from standard curves and normalized to total protein. The lower limit of detection for the urate assay was approximately 2 μM (0.2 nmol/well) using fluorometric detection, enabling reliable detection of changes above basal concentrations present in serum-supplemented conditions.

### Animal studies

All animal studies were approved by the Mayo Clinic Institutional Animal Care and Use Committee. Wild-type (129s1) mice obtained from The Jackson Laboratory were treated by gavage with tolvaptan 75 mg/kg with or without probenecid. *Pkd1*^RC/RC^ mice (C57BL/6J) (Mayo Clinic) were treated with chow containing tolvaptan and/or probenecid from 4 to 16 weeks of age. Acute studies on wild-type mice used short-term gavage. Urine volume and water intake were recorded over 24 hours before sacrifice.

### Immunohistochemistry

Mouse and human kidney tissues (Mayo Clinic Biobank) were paraffin-embedded and stained with H&E or immunoperoxidase using antibodies against AQP2, ABCG2, and GLUT9 (Supplemental Table 4).

### Clinical trial study design

SereNDIpity-pb1 was a phase II, open-label trial evaluating probenecid for tolvaptan-induced polyuria in ADPKD (NCT05190744), conducted at Mayo Clinic (Jacksonville, Florida, USA) and Hôpital du Sacré-Cœur (Montreal, Quebec, Canada).

Adults with ADPKD on stable tolvaptan therapy, urine osmolality < 300 mOsm/kg, and eGFR ≥ 25 mL/min/1.73 m^2^ were eligible. Coprimary endpoints were changes in urine osmolality and 24-hour urine volume. Secondary endpoints included biochemical markers and patient-reported outcomes (67). Probenecid was initiated at 500 mg twice daily and increased to 1,000 mg twice daily as tolerated for up to 90 days, with continued tolvaptan therapy.

#### Study procedures.

Participants were enrolled between January and December 2023, with follow-up through April 2024. Participants were admitted to a Clinical Research Unit (day –1 to day 3). Baseline assessments and dose titration were performed, followed by home administration using THESS device (France). Timed urine collections (every 2 hours from 0800–1400 hours) were obtained on days –1, 1, and 2. Twenty-four–hour urine samples were collected daily from day –1 to day 3, with supersaturation analysis at baseline. Blood and spot urine samples were collected daily.

Follow-up visits on days 7, 15, 30, 45, 60, 75, and 90 included clinical evaluation, laboratory testing, and 24-hour urine collection. Supersaturation studies were repeated on days 15, 45, and 75. At each visit, samples were collected before the morning probenecid dose, with an additional urine sample collected 2 hours postdose. Supersaturation studies were performed using the Mayo Clinic Supersaturation Profile (Test ID: SUPST). The 24-hour urine measurements included volume, pH, calcium, oxalate, phosphate, citrate, magnesium, uric acid, sodium, potassium, chloride, sulfate, ammonium, creatinine, and urea. Supersaturation for calcium oxalate, brushite, hydroxyapatite, uric acid, and sodium urate was calculated using the validated EQUIL2 algorithm (68). Daily symptom diaries recorded thirst, fluid intake, nocturia, and well-being. Safety was monitored by an independent board; probenecid was discontinued at investigator discretion for adverse events.

#### Sample analysis and safety monitoring.

Blood samples were analyzed for serum chemistries including AST, ALT, bilirubin, osmolality, uric acid, magnesium, phosphorus, creatine phosphokinase, and copeptin. Spot urine analyses included osmolality, sodium, potassium, chloride, urea, creatinine, albumin, protein, and microscopy. Baseline urine output was defined as the higher of two 24-hour collection volumes (day –1 or 1). All laboratory testing followed standard clinical protocols. Plasma samples were stored for future analysis.

### Statistics

Experimental data are presented as means ± SD with at least 3 biological replicates. Comparisons used 2-tailed unpaired *t* test or 2-way ANOVA with Dunnett’s or Bonferroni’s correction, as appropriate (*P < 0.05, **P < 0.01, ***P < 0.001, ****P < 0.0001). Clinical endpoints were analyzed using paired 2-tailed *t* tests comparing tolvaptan plus probenecid versus tolvaptan alone. Descriptive statistics summarized baseline characteristics (Supplemental Tables 1 and 2). Analyses were performed in R (v4.2.2), with significance set at *P* < 0.05.

Secondary endpoints and longitudinal trends were analyzed using 2-tailed pairwise *t* tests, comparing each follow-up time point (days 7–90) with baseline, with Bonferroni’s correction for multiple comparisons. Day 90 values were additionally compared with day 15 to assess durability. For contextual comparison, trial participants were compared with a matched historical cohort (*n* = 159) with ADPKD (Mayo Imaging Class 1C–1E) not receiving tolvaptan or probenecid. Groups were matched for age, sex, and eGFR. Continuous variables were compared using independent 2-tailed *t* tests and categorical variables using χ^2^ or Fisher’s exact tests. Missing data were minimal and not imputed, except for posttrial eGFR (*n* = 2), where mean imputation enabled paired analysis.

### Study approval

All animal experiments were approved by the Mayo Clinic IACUC. The clinical study was approved by the IRBs at Mayo Clinic and Hôpital du Sacré-Cœur de Montréal. All participants provided written informed prior consent. The trial was registered January 13, 2022, at ClinicalTrials.gov (NCT05190744).

### Data availability

All data are included in the manuscript and supplemental materials. The Supporting Data Values file provides numerical data underlying figures. Reagents are available upon reasonable request.

## Author contributions

MH, JMM, and GA designed and performed in vitro and in vivo experiments, acquired and analyzed data, prepared figures and tables, and drafted the manuscript. DGB contributed to the design, local conduct, and critical review for the SereNDIpity-pb1 trial and to interpretation of clinical data and manuscript revision. AHB and AG contributed to trial implementation and analysis. EC, PH, and VET provided experimental guidance, contributed to data interpretation, and critically revised the manuscript. SLA and VV contributed to the conceptual development, interpretation of signaling studies, and editorial review and revision. FTC conceived and supervised the overall project, secured funding, oversaw all experimental and clinical aspects of the study, and wrote the final manuscript.

## Conflict of interest

FTC reports research support or contracts from Otsuka Pharmaceuticals, Regulus, Vertex, AstraZeneca, Mezzion, AbbVie, and Natera; holds a patent on “Probenecid as a treatment in ADPKD”; and serves on the Board of Directors of the PKD Foundation. VV reports speaking or consulting honoraria from AstraZeneca, Boehringer Ingelheim, Casdin Capital, Checkpointlp, GondolaBridge, Google Venture, and PrimaryInsight and investigator-initiated research support from Boehringer Ingelheim, Gilead, Jnana Therapeutics, Lexicon, Novo Nordisk, and Maze Therapeutics. SLA reports research support from Quest Diagnostics and David E. Shaw and consulting relationships with Prognostx, David E. Shaw Research, Biossil, Decibel, Entrada, the Chinese University of Hong Kong, and the Medical University of Vienna. VET reports research support from Mironid and GlaxoSmithKline. PCH reports grants or contracts from Espervita, Navitor, Acceleron, Jemincare, Regulus, and PYC Therapeutics; royalties or licenses from Bayer, Sanofi, Vertex, Mitobridge, Maze Therapeutics, Calico Life Sciences, and Arnatar Therapeutics; and consulting fees from Vertex, Mitobridge, Regulus, Otsuka, Janssen, Maze Therapeutics, Caraway Therapeutics, Renasant Bio, Sen Therapeutics, PYC Therapeutics, Nextech, and Arnatar Therapeutics. EC serves as head of the external research advisory board for Neolaia Bio and holds a US provisional patent related to PAPPA-blocking antibodies for ADPKD titled “Materials and Methods for Treating Polycystic Kidney Disease.” DGB reports consulting, speaking, and grant support from Amicus, Sanofi Genzyme, and Otsuka and board service for Amicus.

## Funding support

This work is the result of NIH funding, in whole or in part, and is subject to the NIH Public Access Policy. Through acceptance of this federal funding, the NIH has been given a right to make the work publicly available in PubMed Central.

- NIH National Institute of Diabetes and Digestive and Kidney Diseases R01DK142878 and U54DK144863 (to FTC) and U54 DK137307 (to VV).
- Mayo Clinic Florida RACER, CURE2030, CATALYST, and Team Science Awards (to FTC).
- Mayo Clinic Pirnie Polycystic Kidney Disease Center and Zell Family foundation (to FTC).

## Supplementary Material

Supplemental data

Unedited blot and gel images

Supporting data values

## Version 1 06/16/2026

In-Press Preview

## Version 2 07/15/2026

Electronic publication

## Version 3 07/15/2026

Corrected web address in reference 13
