# Supplementary material for: GLUT9b- and ABCG2-mediated collecting duct urate transport uncovers a vasopressin-independent mechanism of renal water reabsorption
Source: J Clin Invest. 2026 Jun 16;136(14):e197021. doi: 10.1172/JCI197021 (PMC13374851; doi:10.1172/JCI197021)
Supplement: Supplemental data [file jci-136-197021-s017.pdf]

**Supplementary materials**

- **Supplementary Figure 1:** Urate solubility and specificity controls for the *in vitro* assays and validation of genetic modifications in mIMCD3 cells.
- **Supplementary Figure 2:** Additional validation of urate-induced AQP2 trafficking.
- **Supplementary Figure 3:** ABCG2 inhibition and probenecid increase intracellular urate and promote AQP2 apical accumulation in a GLUT9-dependent manner.
- **Supplementary Figure 4:** Validation of urate-induced AQP2 trafficking in MDCK cells and characterization of transporter localization in human tissue, overexpression, and off-target controls.
- **Supplementary Figure 5:** Urate-induced AQP2 trafficking is independent of PANX1 and of NLRP3.
- **Supplementary Figure 6:** Urate-induced AQP2 trafficking is independent of UT-A.
- **Supplementary Figure 7:** Intracellular urate accumulation activates AMPK and requires intact vesicle trafficking for AQP2 apical accumulation.
- **Supplementary Figure 8:** Probenecid attenuates tolvaptan-induced aquaresis without altering kidney volume or renal function.
- **Supplementary Figure 9:** Design and biochemical outcomes of the SereNDIpity-pb1 clinical trial evaluating probenecid in ADPKD patients on tolvaptan.
- **Supplementary Table 1:** Clinical characteristics, adverse events, and outcomes from the SereNDIpity-pb1 trial.
- **Supplementary Table 2:** CONSORT 2025 Checklist for SereNDIpity-pb1 clinical trial.
- **Supplementary Table 3:** Primer sequences used in gene expression and mutagenesis studies.
- **Supplementary Table 4:** Commercial antibodies used for immunoblotting experiments.

**Supplementary Figure 1. Urate solubility and specificity controls for the *in vitro* assays and validation of genetic modifications in mIMCD3 cells**. (**A**) Representative confocal images of apical AQP2 and (**B**) corresponding apical-to-basal fluorescence intensity (FI) ratios showing apical AQP2 trafficking in AQP2-OE mIMCD3 cells treated for 1 h with basal dDAVP (100 nM) and apical urate (500 µM). (**C**) Bright-field imaging mIMCD3 cultures also showed absence of urate crystal formation. (**D**) Dissolved urate at 500 µM formed birefringent crystals as observed in polarized light only in water but not in DMEM/F12, whereas supersaturated urate (>5 mM) produced crystals in both conditions. These results confirmed absence of crystal formation under the conditions used for cell experiments. Urate solutions were prepared in milli-Q water (pH 7) or serum-free DMEM/F12 (pH 7.5) at 37 °C with 1 h continuous agitation before imaging (**E**) Confocal imaging demonstrated that non-transfected cells treated for 1 h with 500 µM urate exhibited no detectable fluorescence emission at 488 nm or 594 nm, and that the signal observed in AQP2-GFP cells treated 1 h with 500 µM urate reflects AQP2-GFP redistribution rather than urate autofluorescence. (**F**, **G**) Urate-induced apical AQP2 accumulation over 1 h was prevented by 30 min pretreatment with rasburicase (5 µg/mL), confirming the requirement for intact urate. (**H**) Corresponding apical-to-basal FI ratios for (**G**). All urate treatments were in FBS-free medium. n≥3 biological replicates. Statistical significance was determined by one-way ANOVA with Dunnett correction in (**B**), 2-way ANOVA with Dunnett correction in (**H**). ***P value* < *0.01, ***P value < 0.001, ****P value < 0.0001*. Scale bar: 25 µm.

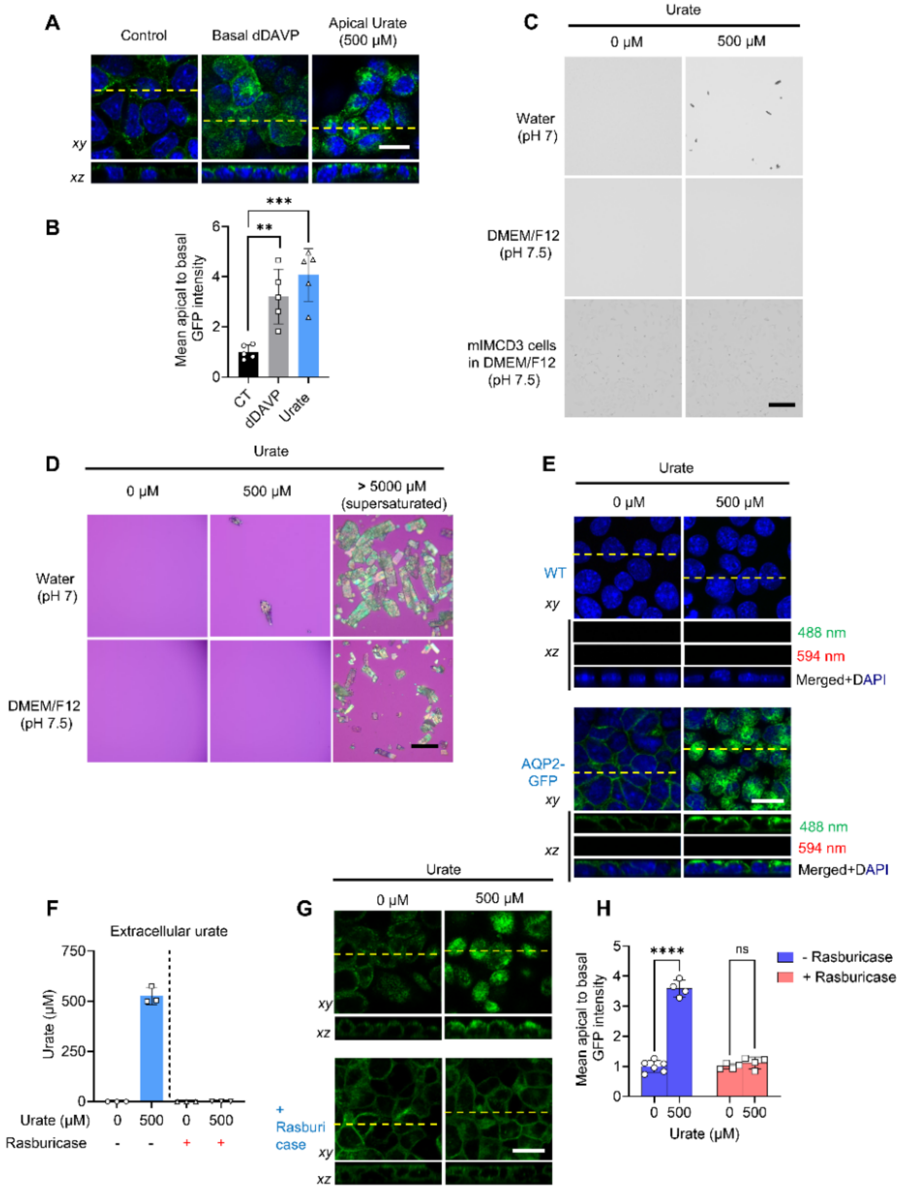

**Supplementary Figure 2. Additional validation of urate-induced AQP2 trafficking.** (**A**) Representative confocal images of AQP2-GFP localization in WT and *Abcg2* KD cells in the absence or presence of dDAVP, tolvaptan (TLV) or probenecid (PB), with (**B**) normalized apical-to-basal FI ratios of AQP2-GFP showing that *ABCG2* KD induces apical accumulation of AQP2. (**C**) Representative confocal images of apical AQP2–GFP localization in wild-type (WT) and *Glut9* knockdown (KD) mIMCD3 cells treated with ABCG2 inhibitors benzbromarone (Benz, 10 µM), resveratrol (Res, 0.5 µM), febuxostat (Febux, 100 µM), or novobiocin (Novo, 100 µM). (**D**) Normalized apical-to-basal FI ratios demonstrating that apical AQP2 trafficking is abolished in GLUT9-deficient cells. Panels (A) and (C) share the same WT control, as experiments were performed in parallel. n≥3 biological replicates. Statistical significance in (**B** and **D**) was determined by 2-way ANOVA with Bonferroni correction. ***P < 0.01, ***P < 0.001, ****P < 0.0001*. Scale bar: 25 µm.

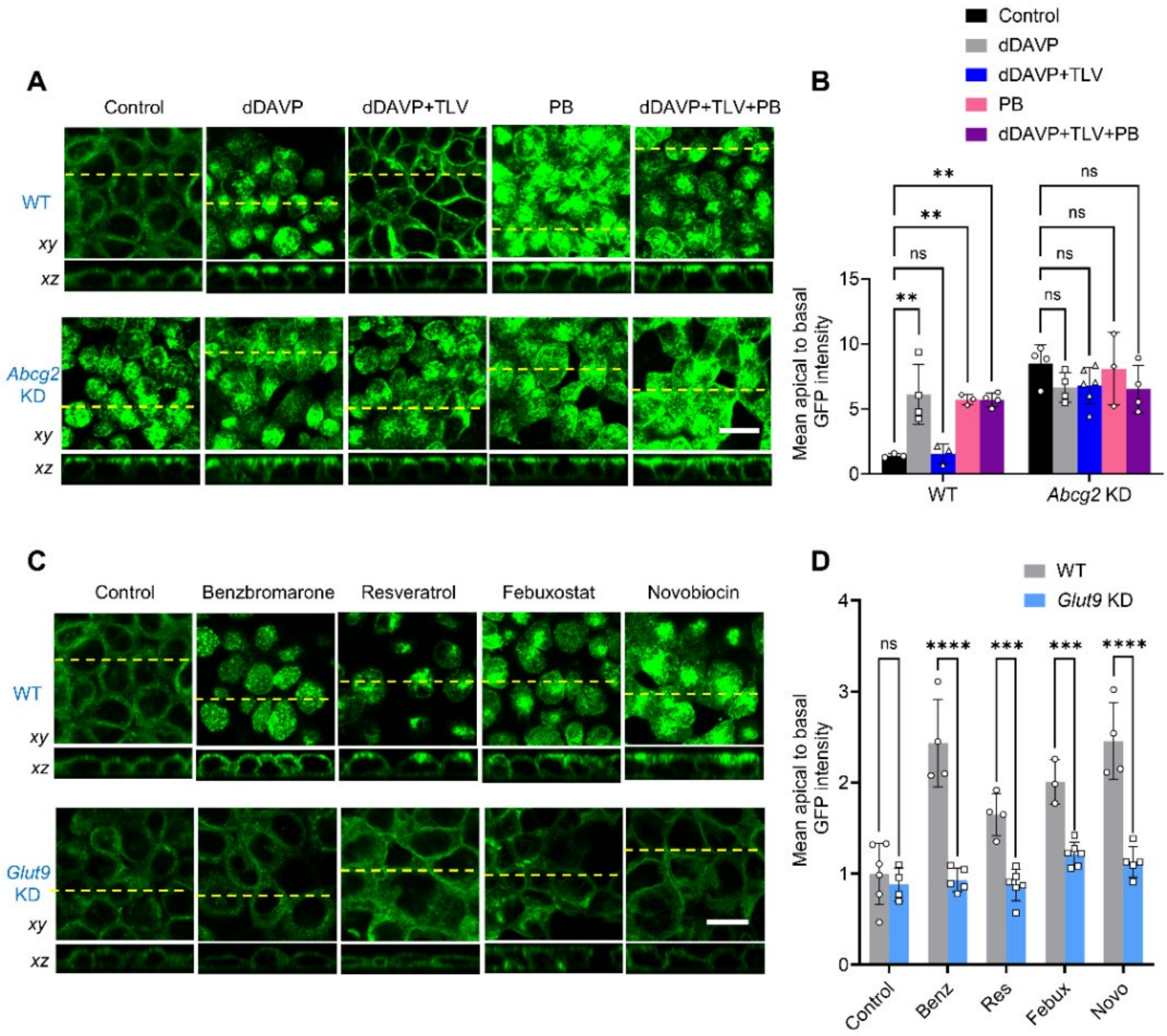

**Supplementary Figure 3. Additional validation of urate-induced AQP2 trafficking.** (**A**) Confocal images of AQP2-GFP localization in representative WT or *Glut9* knock down cells in the absence (control) or presence of Ko143 or probenecid for 1, 12 or 24 h. **B** and **D** show normalized apical-to-basal FI and (**C**) normalized intracellular urate measurements, confirming gradual urate accumulation and corresponding apical AQP2 accumulation over 12–24 hours. n≥3 biological replicates. Statistical significance was determined by 2-way ANOVA with Dunnett correction. ***P < 0.01, ****P < 0.0001*. Scale bar: 25 μm.

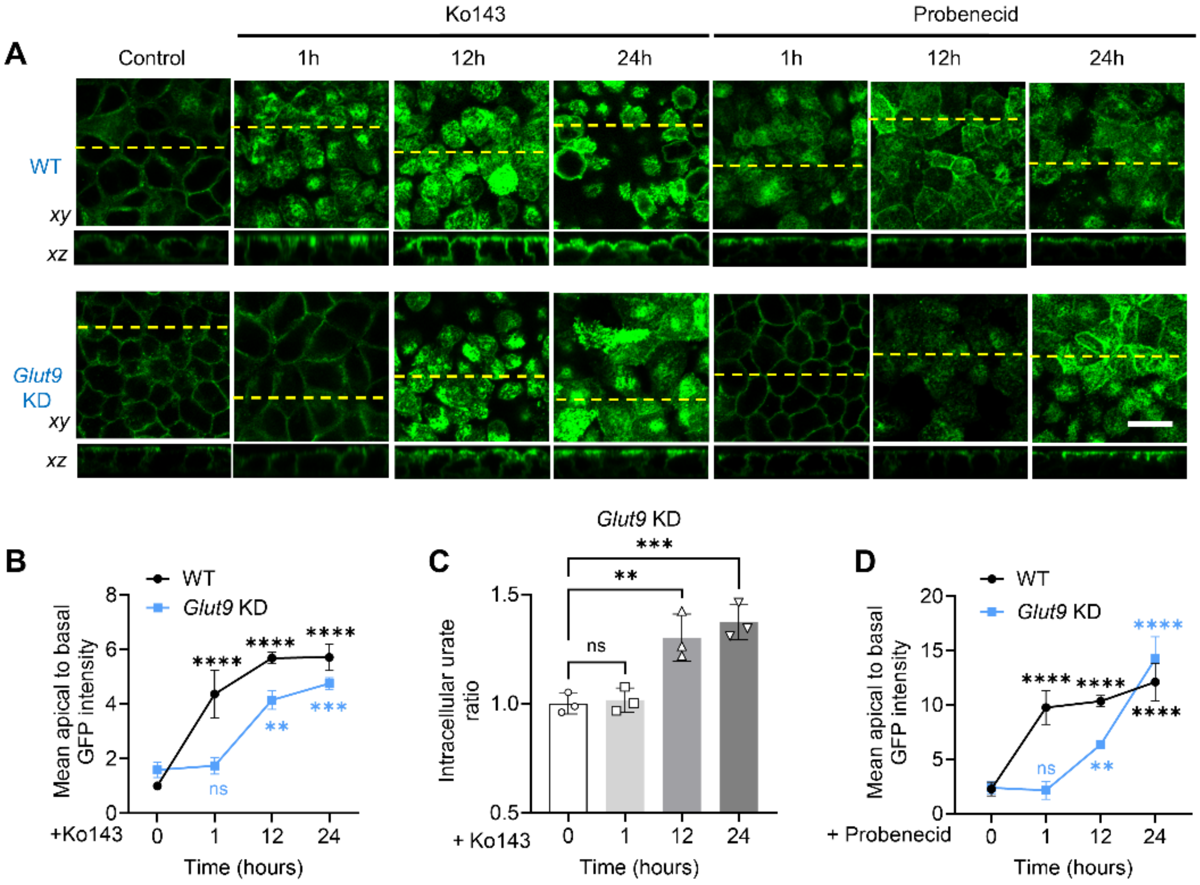

**Supplementary Figure 4. Validation of urate-induced AQP2 trafficking in MDCK cells and characterization of transporter localization in human tissue, overexpression and off-target controls.** (**A**) Confocal images of representative polarized MDCK cells expressing AQP2–GFP after 1 h treatment with dDAVP (100 nM), Probenecid (100 μM), or Ko143 (1 μM). (**B**) Normalized apical-to-basal FI demonstrating apical AQP2 accumulation across species. (**C**) Immunohistochemically stained human kidney sections showing ABCG2 localization at collecting duct (CD) luminal surface and GLUT9 expression in both CD and proximal convoluted tubule (PCT) basolateral membrane. Scale bar, 75 μm. (**D**) Urate quantitation in 10% and 100% FBS. (**E**) GLUT9b overexpression in mIMCD3 cells increased intracellular urate and (**F**, **G**) induced spontaneous apical localization of AQP2. (**H**) Western blot validation of GLUT9b overexpression and (**I**) corresponding mean densitometric quantitation, with accompanying increase in ABCG2 expression. All urate treatments were performed in FBS-free medium. n≥3 biological replicates. Statistical significance was determined by unpaired *t* test for (**E**), one-way ANOVA with Dunnett correction for (**B**), and 2-way ANOVA with Bonferroni correction for (**G** and **I**). $^{**}P < 0.01$, $^{****}P < 0.0001$. Scale bar: 25 μm unless otherwise indicated.

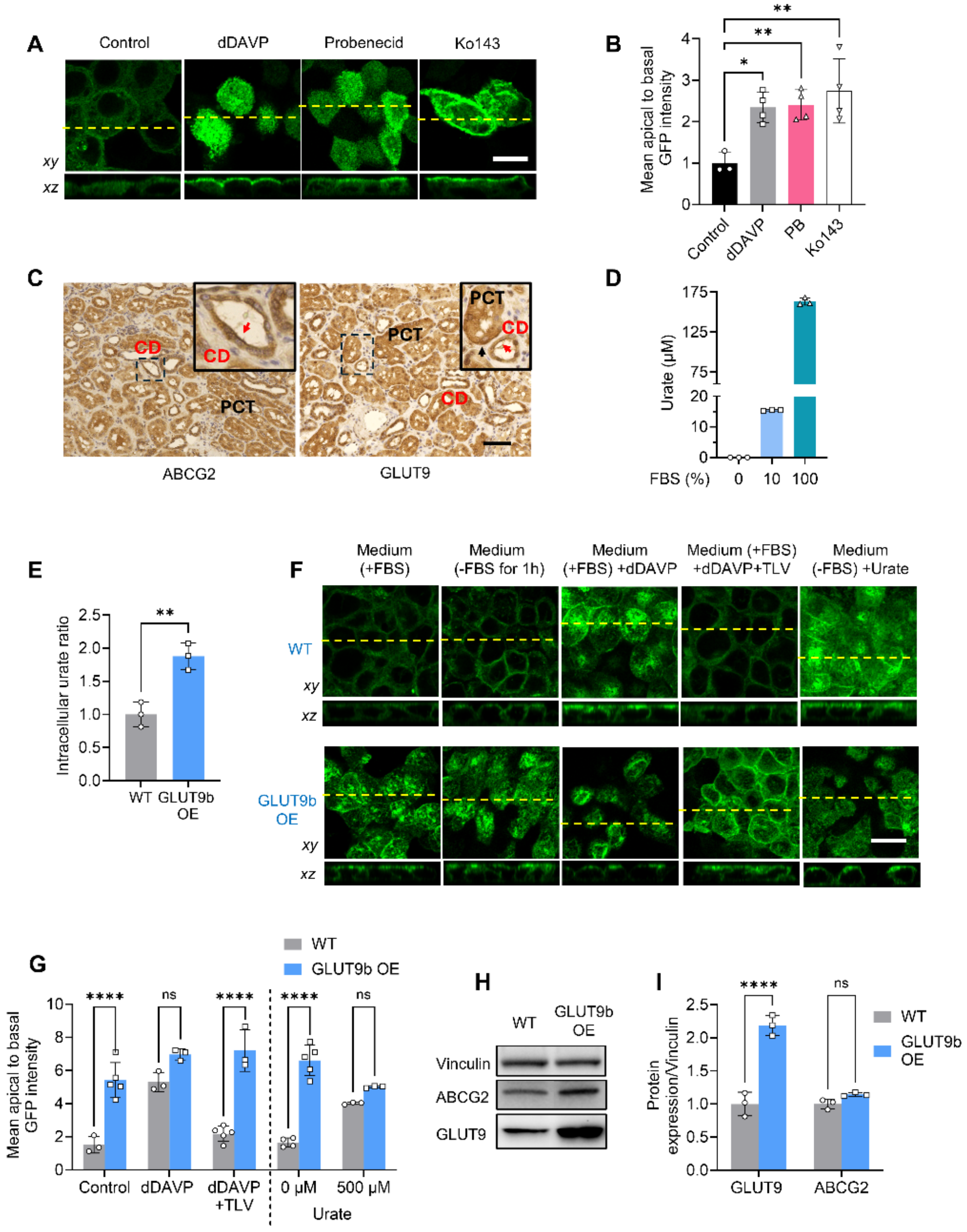

**Supplementary Figure 5. Urate-induced AQP2 trafficking is independent of PANX1, NLRP3** (**A**) Confocal images of representative cells with *Panx1* or *Nlrp3* knockdown treated with dDAVP (100 nM) and urate (500 μM) for 1 h and (**B**) normalized apical-to-basal FI ratios for (**A**). (**C**, **E**) Representative immunoblots and (**D**, **F**) mean densitometric quantitation confirming efficient siRNA knockdown of (**C**, **D**) PANX1 and (**E**, **F**) NLRP3. n≥3 biological replicates. Statistical significance was determined by unpaired *t* test for (**D** and **F**) and by 2-way ANOVA with Dunnett correction for (**B**). ***P < 0.01, ***P <* *0.001* and ****$P < 0.0001$ . Scale bar, 25 μm.

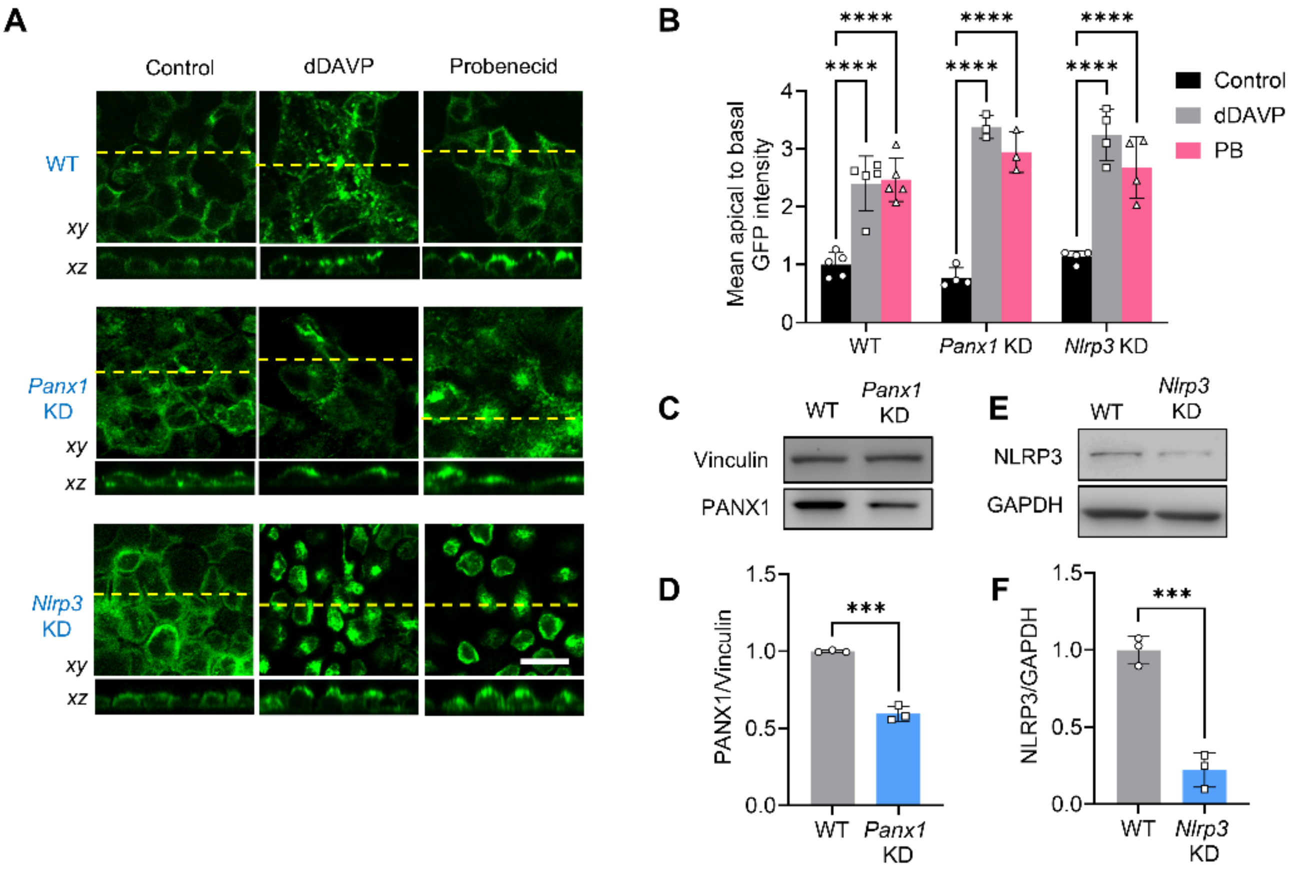

**Supplementary Figure 6. Urate-induced AQP2 trafficking is independent of UT-A.** (**A**) Confocal images of representative WT and *Slc14a2* KD cells treated for 1 h with dDAVP (100 nM) and urate (500 μM) in the absence or presence of 800 mM urea. (**B**) Corresponding normalized apical-to-basal FI ratios, showing apical AQP2 redistribution in both cell lines. (**C**) Representative immunoblot and (**D**) mean densitometric quantitation confirming *Slc14a2* knockdown. n≥3 biological replicates. Statistical significance was determined by 2-way ANOVA with Bonferroni correction for (**B**) and by unpaired *t* test for (**D**). $^{**}P < 0.01$, $^{***}P < 0.001$; ns, not significant. Scale bar, 25 μm.

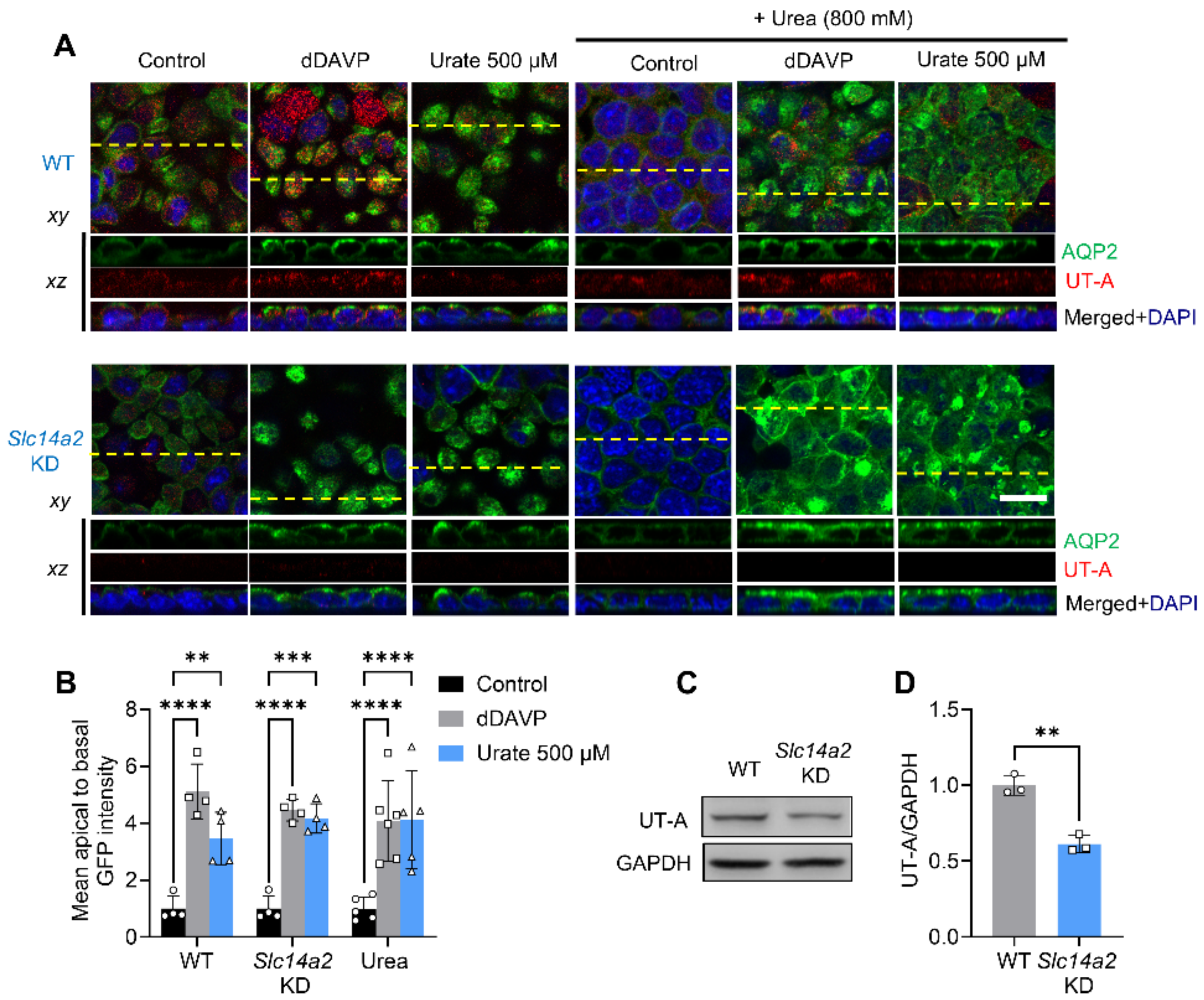

**Supplementary Figure 7. Intracellular urate accumulation activates AMPK and requires intact vesicle trafficking for apical accumulation of AQP2.** (**A**) Immunoblot and (**B**) densitometric quantitation of total AMPKα and p-AMPKα (Thr172) after 12 h urate treatment, showing elevation in pAMPK/AMPK ratio. (**C**) Confocal images and (**D**) corresponding normalized apical-to-basal FI of representative mIMCD3 AQP2-GFP cells, showing that 30 min pre-treatment with colchicine (Colch, 10 µM) or acute cold shock (C.S.) abolishes AQP2 apical translocation induced by 1 h treatment with dDAVP (10 nM) or urate (500 µM). (**E-N**) qPCR analyses validated targeted knockdowns using siRNA or overexpression of *Aqp2, V2r, Glut9, Abcg2, Panx1, Nlrp3, Slc14a2, Prkaa1, and Prkaa2* in the modified cell lines studied, with *Gapdh* as "housekeeping" normalization control gene. n≥3 biological replicates. Statistical significance was determined by unpaired *t* test for (**E**–**N**), one-way ANOVA with Dunnett correction for (**B**) and 2-way ANOVA with Dunnett correction for (**D**). $^{*}P < 0.05$, $^{**}P < 0.01$, $^{***}P < 0.001$, $^{****}P < 0.0001$. Scale bar, 25 µm.

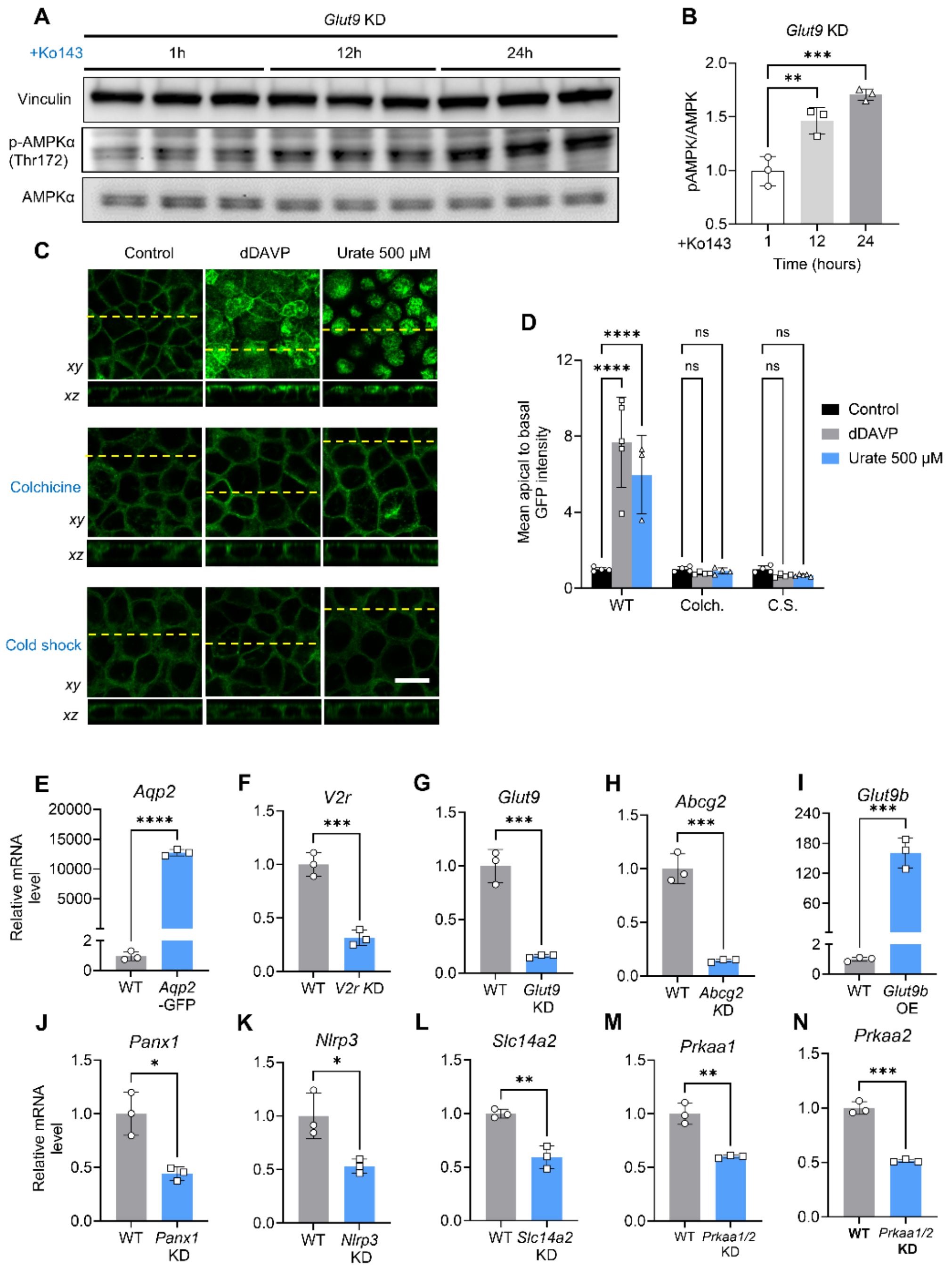

**Supplementary Figure 8. Probenecid attenuates tolvaptan-induced aquaresis with improvement in kidney volume and renal function.** (**A**) Change in total kidney volume (ΔTKV), showing improvement with tolvaptan/probenecid combination. (**B**) Blood urea nitrogen (BUN) levels across treatment groups, showing no significant differences. (**C**) Serum creatinine levels, showing reduction with tolvaptan/probenecid combination. Data are presented as mean ±SD with individual data points representing biological replicates. Statistical significance was determined by one-way ANOVA with Dunnett correction. **P < 0.05, **P < 0.01.*

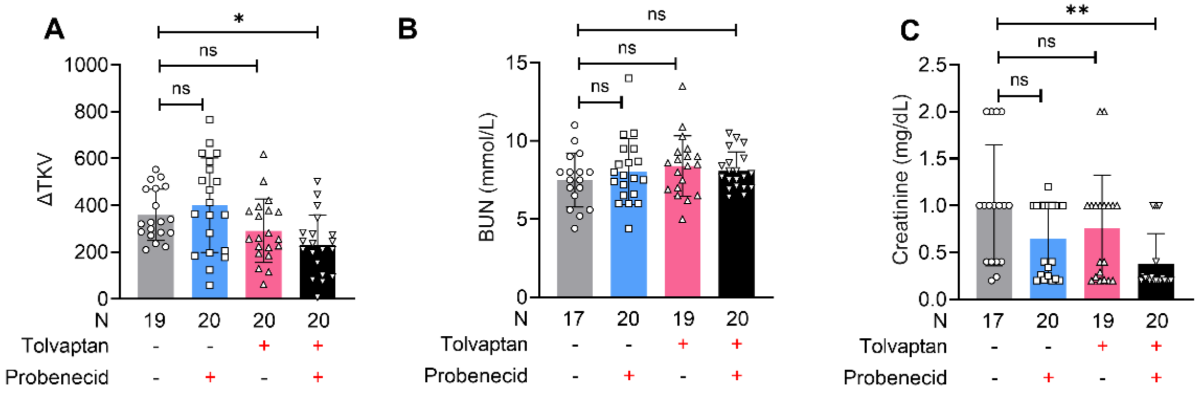

**Supplementary Figure 9. Design and biochemical outcomes of the SereNDIpity-pb1 clinical trial evaluating probenecid in ADPKD patients on tolvaptan.** (**A**) Mean estimated glomerular filtration rates (eGFR) during the 90-day follow-up. (**B**) Trends in liver function tests (AST, ALT) and total bilirubin over the study duration. (**C**) Serum uric acid levels throughout treatment. (**D**) Paired serum copeptin values during the study period. $^{*}P < 0.05$; $^{**}P < 0.001$. BL, baseline before initiation of treatment.

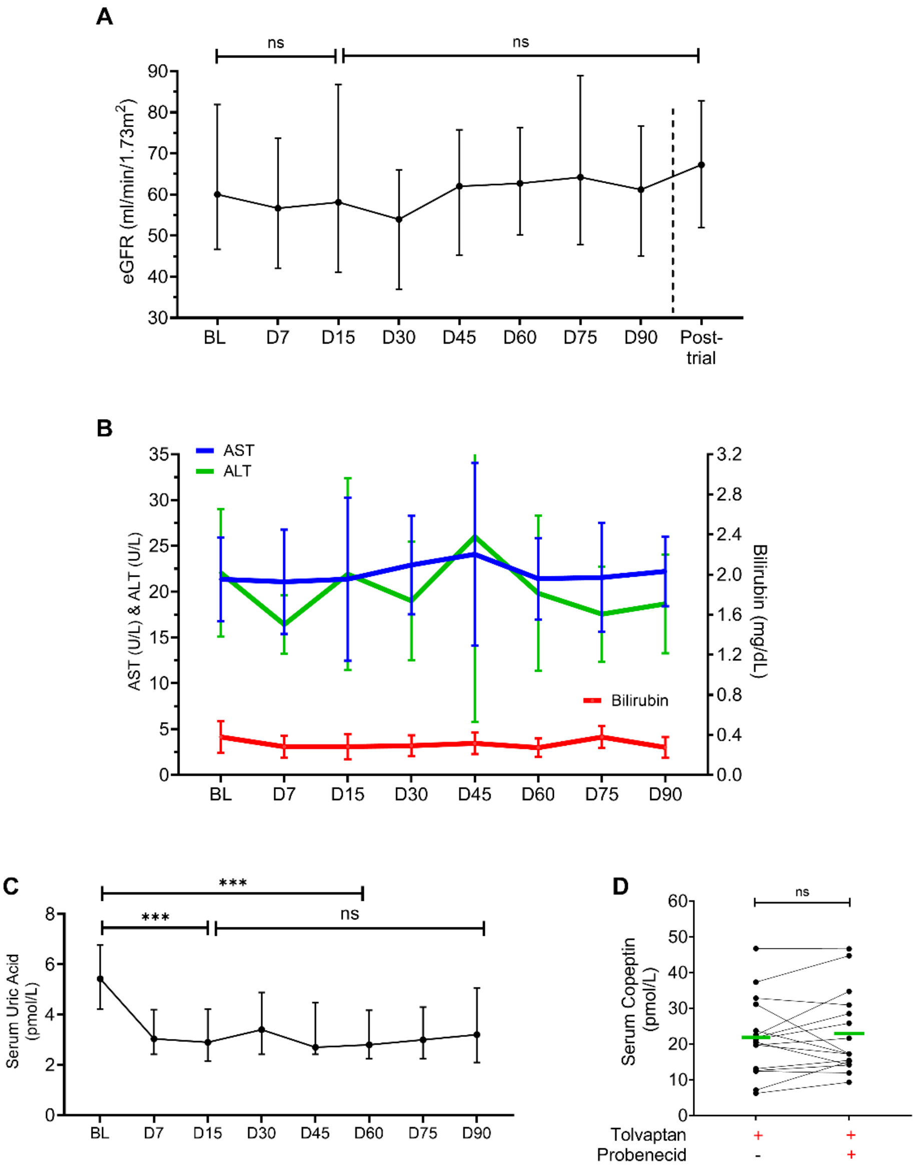

**Supplementary Table 1. Clinical characteristics, adverse events, and outcomes from the SereNDIpity-pb1 trial. a,** Baseline demographic and clinical characteristics of ADPKD patients receiving tolvaptan in the SereNDIpity-pb1 trial (N = 17) vs. matched historical control cohort not treated with tolvaptan or probenecid (N = 159), matched by age, sex, and eGFR. **b,** Key clinical outcomes following ≥7 days of probenecid therapy, including changes in 24-hour urine volume, nocturia frequency, morning urine osmolality, serum uric acid, serum copeptin, and ADPKD-Impact Scale (ADPKD-IS) scores. **c,** Safety data for patients with longitudinal follow-up (N = 14), including eGFR, AST, ALT, and bilirubin at baseline, during probenecid treatment, and post-discontinuation. Summary of non-serious adverse events reported during the study period. Two-tailed paired t-tests. P-values < 0.05 were considered statistically significant.

**a) Demographics**

| | **ADPKD on tolvaptan** | **ADPKD not on tolvaptan** | **p-value** |
|---|---|---|---|
| **N** | 17 | 159 | |
| **Females, n (%)** | 9 (52.9%) | 88 (55.3%) | 0.85 |
| **Caucasians, n (%)** | 16 (94.1%) | 143 (89.9%) | 0.58 |
| **Age, years, mean (SD)** | 46.6 (10.5) | 46.2 (12.3) | 0.88 |
| **BMI, kg/m$^2$, mean (SD)** | 26.7 (4.8) | 28.2 (6.6) | 0.36 |
| **eGFR at baseline, mL/min/1.73 m$^2$, mean (SD)** | 66.6 (22.5) | 63.4 (25.9) | 0.62 |
| **24-hour urine volumes, mL, mean (SD)** | 6235 (2046) | 2343 (1031) | <0.0001 |
| **Hypertension, n (%)** | 15 (88.2%) | 149 (97.3%) | 0.39 |
| **CKD stage at baseline** | | | 0.76 |
| *Stage 1-2, n (%)* | 9 (52.9%) | 78 (49.1%) | |
| *Stage 3, n (%)* | 8 (47.1%) | 81 (50.9%) | |

| **Tolvaptan dosage** | **ADPKD on tolvaptan** |
|---|---|
| *45/15, n (%)* | 7 (41.2%) |
| *60/30, n (%)* | 8 (47.1%) |
| *90/30, n (%)* | 2 (11.8%) |

**b) Outcomes**

| | **Tolvaptan (T)** | **Tolvaptan and probenecid (TP)** | **p-value** |
|---|---|---|---|
| **N** | 14 | 14 | |
| **24-hour urine volume, mL, mean (SD)** | 6798 (1693) | 4662 (1168) | <0.0001 |
| **Urine osmolarity, mOsm/Kg, mean (SD)** | 248 (126) | 324 (85) | 0.017 |
| **ADPKD-IS, mean (SD)** | 32.4 (11.6) | 27.4 (11.4) | <0.0001 |
| **Nocturia episode** — **N** | 10 | 10 | 0.0004 |
| **Nocturia episode** — **Mean (SD)** | 3.9 (1.4) | 0.9 (0.6) | |
| **Serum uric acid, mg/L, mean (SD)** | 5.4 (1.3) | 3.2 (1.1) | <0.0001 |
| **Serum copeptin, pmol/L, mean (SD)** | 21.8 (10.9) | 22.8 (11.4) | 0.55 |
| **Serum sodium, mmol/L, mean (SD)** | 140.7 (2.5) | 140.1 (1.6) | 0.56 |

| c) Safety Markers | | | | | |
|---|---|---|---|---|---|
| | | | | p-value | |
| | Tolvaptan (T) | Tolvaptan and probenecid (TP) | Tolvaptan Post-trial (TpostTP) | T vs. TP | TP vs. TpostTP |
| **N** | 14 | 14 | 14 | | |
| **eGFR, mL/min/1.73m$^2$, mean (SD)** | 71.9 (21.1) | 63.9 (19.8) | 70.4 (17.7) | 0.003 | 0.038 |
| **AST, U/L, mean (SD)** | 20.9 (4.1) | 21.6 (5.0) | 18.9 (2.8) | 0.51 | 0.29 |
| **ALT, U/L, mean (SD)** | 21.4 (4.7) | 20.4 (6.5) | 17.9 (3.9) | 0.51 | 0.32 |
| **Bilirubin, mg/dL, mean (SD)** | 0.42 (0.19) | 0.33 (0.10) | 0.52 (0.16) | 0.14 | 0.003 |

| Adverse events* | ADPKD on tolvaptan |
|---|---|
| **N** | 17 |
| **Participants with AEs, n (%)** | 9 (52.9%) |
| **Serious AEs, n (%)** | 0 (0%) |
| **AEs leading to withdrawal, n (%)** | 2 (11.8%) |
| **Gastrointestinal upset, n (%)** | 6 (35.2%) |
| *Nausea, n (%)* | 6 (35.2%) |
| *Vomiting, n (%)* | 1 (5.9%) |
| *Decreased appetite, n (%)* | 1 (5.9%) |
| *Heartburn, n (%)* | 1 (5.9%) |
| **Headaches, n (%)** | 3 (17.6%) |
| **Rash, n (%)** | 4 (23.5%) |
| **Lower limb edema, n (%)** | 1 (5.9%) |
| **Hemorrhoids, n (%)** | 1 (5.9%) |
| **High blood pressure readings, n (%)** | 1 (5.9%) |
| **Pleuritic chest pain, n (%)** | 1 (5.9%) |

*Adverse events are not mutually exclusive; a single patient may have experienced more than one event.
ADPKD: autosomal dominant polycystic kidney disease; ADPKD-IS: Autosomal Dominant Polycystic Kidney Disease–Impact Scale; ALT: alanine aminotransferase; AST: aspartate aminotransferase; BMI: body mass index; CKD: chronic kidney disease; eGFR: estimated glomerular filtration rate; SD: standard deviation

**Supplementary Table 2. CONSORT 2025 Checklist for SereNDIpity-pb1 clinical trial**:

| Section/topic | No | CONSORT 2025 checklist item description | Reported on page no. |
|---|---|---|---|
| **Title and abstract** | | | |
| Title and structured abstract | 1a | Identification as a randomised trial | N/A (non-randomized |
| | 1b | Structured summary of the trial design, methods, results, and conclusions | P.13, lines. 308-337 |
| **Open science** | | | |
| Trial registration | 2 | Name of trial registry, identifying number (with URL) and date of registration | P.24, lines 573-575 |
| Protocol and statistical analysis plan | 3 | Where the trial protocol and statistical analysis plan can be accessed | P.26, lines 617-618 |
| Data sharing | 4 | Where and how the individual de-identified participant data (including data dictionary), statistical code and any other materials can be accessed | P.24, lines 581-596 |
| Funding and conflicts of interest | 5a | Sources of funding and other support (eg, supply of drugs), and role of funders in the design, conduct, analysis and reporting of the trial | P.27, lines 659-663 |
| | 5b | Financial and other conflicts of interest of the manuscript authors | P.27, lines 659-663 |
| **Introduction** | | | |
| Background and rationale | 6 | Scientific background and rationale | P.13, lines. 308-337 |
| Objectives | 7 | Specific objectives related to benefits and harms | P.24, lines 581-601 |
| **Methods** | | | |
| Patient and public involvement | 8 | Details of patient or public involvement in the design, conduct and reporting of the trial | N/A |
| Trial design | 9 | Description of trial design including type of trial (eg, parallel group, crossover), allocation ratio, and framework (eg, superiority, equivalence, non-inferiority, exploratory) | P.24, lines 572-618 |
| Changes to trial protocol | 10 | Important changes to the trial after it commenced including any outcomes or analyses that were not prespecified, with reason | N/A |
| Trial setting | 11 | Settings (eg, community, hospital) and locations (eg, countries, sites) where the trial was conducted | P.24 lines 573-575 |
| Eligibility criteria | 12a | Eligibility criteria for participants | P.24 lines 576-580 |
| | 12b | If applicable, eligibility criteria for sites and for individuals delivering the interventions (eg, surgeons, physiotherapists) | N/A |
| Intervention and comparator | 13 | Intervention and comparator with sufficient details to allow replication. If relevant, where additional materials describing the intervention and comparator (eg, intervention manual) can be accessed | P.24, line 581-586 |
| Outcomes | 14 | Prespecified primary and secondary outcomes, including the specific measurement variable (eg, systolic blood pressure), analysis metric (eg, change from baseline, final value, time to event), method of aggregation (eg, median, proportion), and time point for each outcome | P.25 lines 597-615 |
| Harms | 15 | How harms were defined and assessed (eg, systematically, non-systematically) | P.24 lines 581-596 |
| Sample size | 16a | How sample size was determined, including all assumptions supporting the sample size calculation | Pilot and feasibility |
| | 16b | Explanation of any interim analyses and stopping guidelines | P.24, lines 597-601 |
| Randomisation: | | | |
| Sequence generation | 17a | Who generated the random allocation sequence and the method used | N/A |
| | 17b | Type of randomisation and details of any restriction (eg, stratification, blocking and block size) | N/A |

| | | | Reported on page no. |
|---|---|---|---|
| Allocation concealment mechanism | 18 | Mechanism used to implement the random allocation sequence (eg, central computer/telephone; sequentially numbered, opaque, sealed containers), describing any steps to conceal the sequence until interventions were assigned | N/A |
| Implementation | 19 | Whether the personnel who enrolled and those who assigned participants to the interventions had access to the random allocation sequence | N/A |
| Blinding | 20 a | Who was blinded after assignment to interventions (eg, participants, care providers, outcome assessors, data analysts) | N/A |
| | 20 b | If blinded, how blinding was achieved and description of the similarity of interventions | N/A |
| Statistical methods | 21 a | Statistical methods used to compare groups for primary and secondary outcomes, including harms | P.24, lines 602-607 |
| | 21 b | Definition of who is included in each analysis (eg, all randomised participants), and in which group | N/A |
| | 21c | How missing data were handled in the analysis | P.25, lines 613-615 |
| | 21 d | Methods for any additional analyses (eg, subgroup and sensitivity analyses), distinguishing prespecified from post hoc | N/A |
| **Results** | | | |
| Participant flow, including flow diagram | 22 a | For each group, the numbers of participants who were randomly assigned, received intended intervention, and were analysed for the primary outcome | P.13, lines 309-337 |
| | 22 b | For each group, losses and exclusions after randomisation, together with reasons | N/A |
| Recruitment | 23 a | Dates defining the periods of recruitment and follow-up for outcomes of benefits and harms | P.24, lines 581-596 |
| | 23 b | If relevant, why the trial ended or was stopped | N/A |
| Intervention and comparator delivery | 24 a | Intervention and comparator as they were actually administered (eg, where appropriate, who delivered the intervention/comparator, how participants adhered, whether they were delivered as intended (fidelity)) | P.24, line 581-586 |
| | 24 b | Concomitant care received during the trial for each group | P.24, lines 597-615 |
| Baseline data | 25 | A table showing baseline demographic and clinical characteristics for each group | Supp Table 2 |
| Numbers analysed, outcomes and estimation | 26 | For each primary and secondary outcome, by group:<br>● the number of participants included in the analysis<br>● the number of participants with available data at the outcome time point<br>● result for each group, and the estimated effect size and its precision (such as 95% confidence interval)<br>● for binary outcomes, presentation of both absolute and relative effect size | P.49 Fig 9 + Sup. Fig 6 Sup. Table 2 |
| Harms | 27 | All harms or unintended events in each group | Supp Table 2 |
| Ancillary analyses | 28 | Any other analyses performed, including subgroup and sensitivity analyses, distinguishing pre-specified from post hoc | N/A |
| **Discussion** | | | |
| Interpretation | 29 | Interpretation consistent with results, balancing benefits and harms, and considering other relevant evidence | P.13, lines 308-337 |
| Limitations | 30 | Trial limitations, addressing sources of potential bias, imprecision, generalisability, and, if relevant, multiplicity of analyses | P.13, lines 308-337 |

Citation: Hopewell S, Chan AW, Collins GS, Hróbjartsson A, Moher D, Schulz KF, et al. CONSORT 2025 Statement: updated guideline for reporting randomised trials. BMJ. 2025; 388:e081123. https://dx.doi.org/10.1136/bmj-2024-081123
© 2025 Hopewell et al. This is an Open Access article distributed under the terms of the Creative Commons Attribution License (https://creativecommons.org/licenses/by/4.0/), which permits unrestricted use, distribution, and reproduction in any medium, provided the original work is properly cited.

**Supplementary Table 3. Primer sequences used in gene expression and mutagenesis studies. a,** Oligonucleotide sequences used for quantitative real-time PCR (qPCR) of target genes. **b,** Primer pairs designed for site-directed mutagenesis to generate AQP2 cDNAs and related constructs.

| **Supplementary Table 3a.** Sequences for the primers used in real time PCR | | | |
|---|---|---|---|
| **Genes** | **Species** | **Primers forward (5' – 3')** | **Primers reverse (5' – 3')** |
| *Aqp2* | Mouse | TTGCCATGTCTCCTTCCTTC | GGTCAGGAAGAGCTCCACAG |
| *Avpr2 or V2r* | Mouse | TGACCGAGACCCGCTGTTA | CGACCCCGTCGTATTAGGG |
| *Glut9* | Mouse | CCACGCTACCTGCTCTTGGA | TTTCCCAAGAACGTTGGA |
| *Abcg2* | Mouse | TCGCAGAAGGAGATGTGTTGAG | CCAGAATAGCATTAAGGCCAG |
| *Panx1* | Mouse | AGCCAGAGAGTGGAGTTCAAAGA | CATTAGCAGGACGGATTCAGAA |
| *Nlrp3* | Mouse | TCACAACTCGCCCAAGGAGGAA | AAGAGACCACGGCAGAAGCTAG |
| *Slc14a2* | Mouse | CCGCATCTACTTCCTGACAGTG | CGATGTGGAACACACTCCTACG |
| *Prkaa1* | Mouse | GGTGTACGGAAGGCAAAATGGC | CAGGATTCTTCCTTCGTACACGC |
| *Prkaa2* | Mouse | CTGAAGCCAGAGAATGTGCTGC | GAGATGACCTCAGGTGCTGCAT |
| *Gapdh* | Mouse | TGAGCAAGAGAGGCCCTATC | AGGCCCCTCCTGTTATTATG |
| **Supplementary Table 3a 3b.** Sequences for the primers used in point mutagenesis | | | |
| S256A Aqp2 | Mouse | GGCGGCAGGCAGTGGAGCTGCA CTCTCC | CAGCTCCACTGCCTGCCGCCGC CGCACT |

**Supplementary Table 4. Commercial antibodies used for immunoblotting experiments.** List of primary and secondary antibodies used in Western blotting, including target specificity, host species, vendor, catalog numbers, and working dilutions.

| Target Protein | Antibody Source | Catalog Number | Dilution | Blocking Solution (5%) | Incubation time and temperature |
|---|---|---|---|---|---|
| AQP2 | Santa Cruz Biotechnology | sc-515770 | 1:200 | Skim Milk | 16h at 4°C |
| AQP2 | Abcam | ab199975 | 1:50 | BSA | 1h at 22°C |
| Vinculin | Santa Cruz Biotechnology | sc-73614 | 1:200 | Skim Milk | 16h at 4°C |
| GAPDH | Santa Cruz Biotechnology | sc-32233 | 1:200 | Skim Milk | 16h at 4°C |
| AQP2-pS256 | Biorbyt | orb155711 | 1:1000 | Skim Milk | 16h at 4°C |
| PANX1 | Abcam | ab124131 | 1:1000 | Skim Milk | 16h at 4°C |
| NLRP3 | Abcam | ab263899 | 1:1000 | Skim Milk | 16h at 4°C |
| GLUT9 | Thermo Fisher Scientific | PA5-22971 | 1:1000 | Skim Milk | 16h at 4°C |
| SLC14A2/UT-A | Bioss | BS-11801R | 1:1000 | Skim Milk | 16h at 4°C |
| Phospho-AMPKα (Thr172) | Cell Signaling | 2531S | 1:1000 | BSA | 16h at 4°C |
| AMPKα | Proteintech | 10929-2-AP | 1:2000 | Skim Milk | 1.5h at 22°C |
| ABCG2 | Proteintech | 27286-1-AP | 1:1000 | 1% BSA | 1.5h at 22°C |
| EEA1 | Cell signaling | 2411 | 1:100 | 1% BSA | 16h at 4°C |
| Rab7 | Cell signaling | 9367S | 1:100 | 1% BSA | 16h at 4°C |
| Rab11 | Cell signaling | 5589S | 1:50 | 1% BSA | 16h at 4°C |
| CD81 | Cell signaling | 10037 | 1:200 | 1% BSA | 16h at 4°C |
| Goat anti-Rabbit, Alexa Fluor-594 | Thermo Fisher | A-11012 | 2 µg/mL | 1% BSA | 1h at 22°C |
| ENVISION+/HRP Rabbit | Agilent | K400311-2 | 100 µl | 1% BSA | 1h at 22°C |
| Goat anti-Rabbit (HRP) | Thermo Fisher Scientific | 31460 | 1:5000 | Skim Milk | 1h at 22°C |
| Goat anti-Mouse (HRP) | Thermo Fisher Scientific | 31430 | 1:5000 | Skim Milk | 1h at 22°C |
