# Supplementary material for: GLUT9b- and ABCG2-mediated collecting duct urate transport uncovers a vasopressin-independent mechanism of renal water reabsorption
Source: J Clin Invest. 2026 Jun 16;136(14):e197021. doi: 10.1172/JCI197021 (PMC13374851; doi:10.1172/JCI197021)

Figure 1B

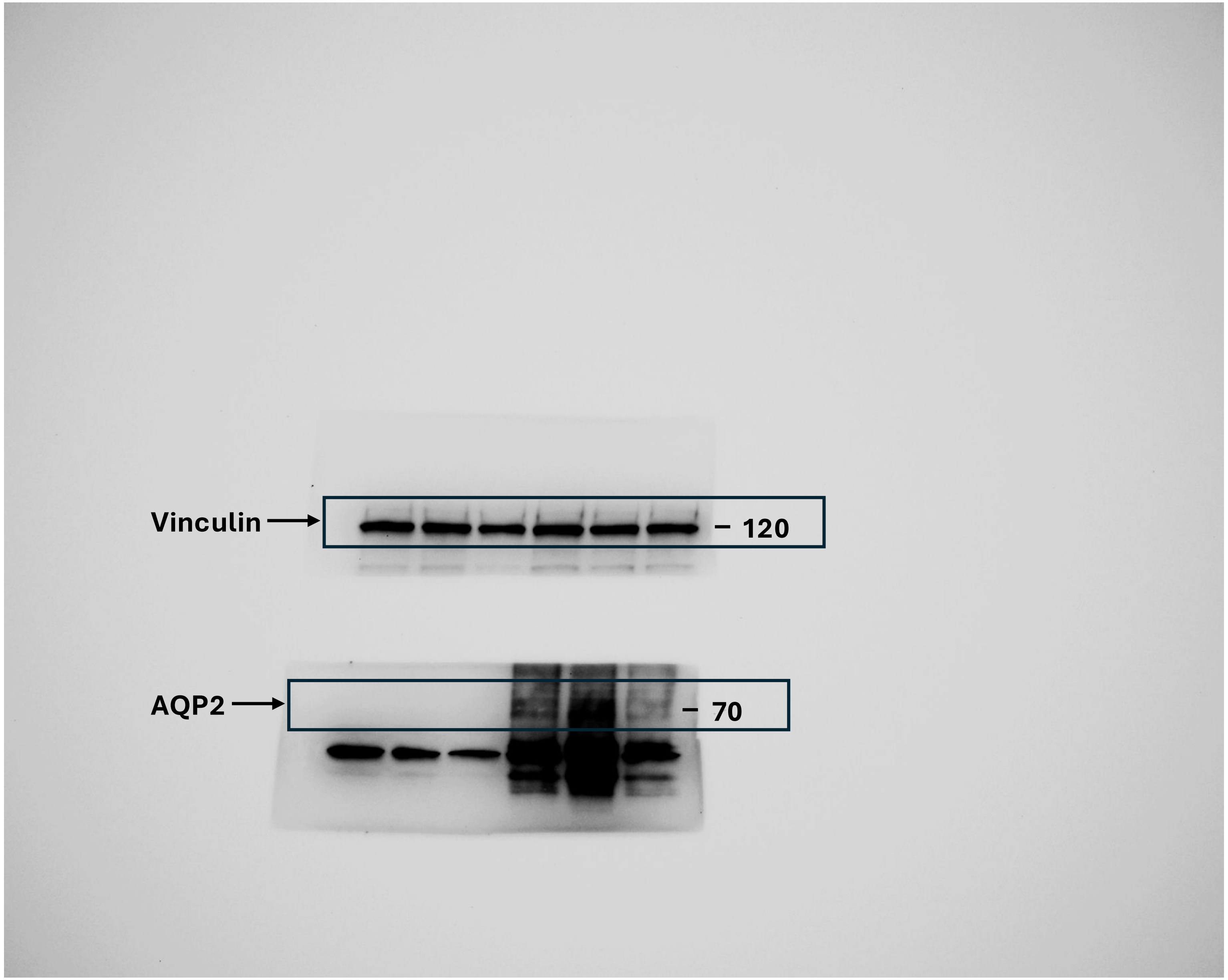

Figure 2C, 5C

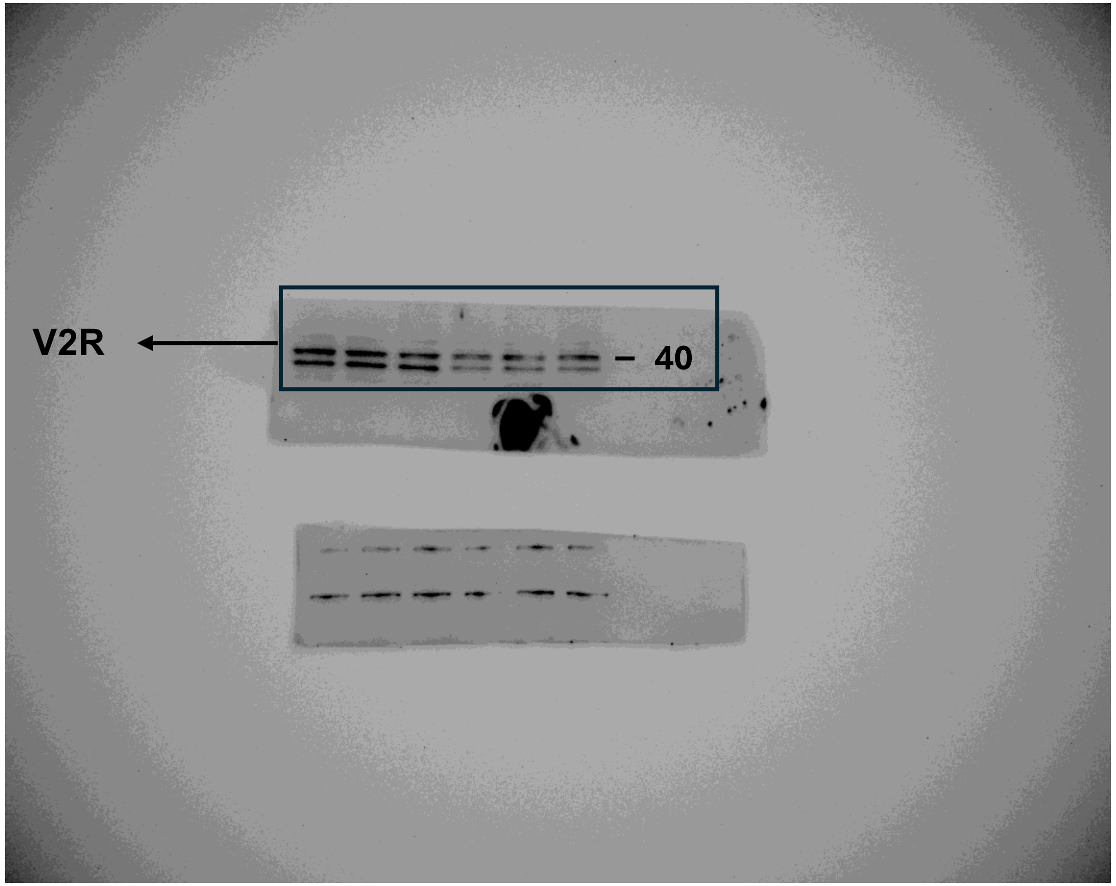

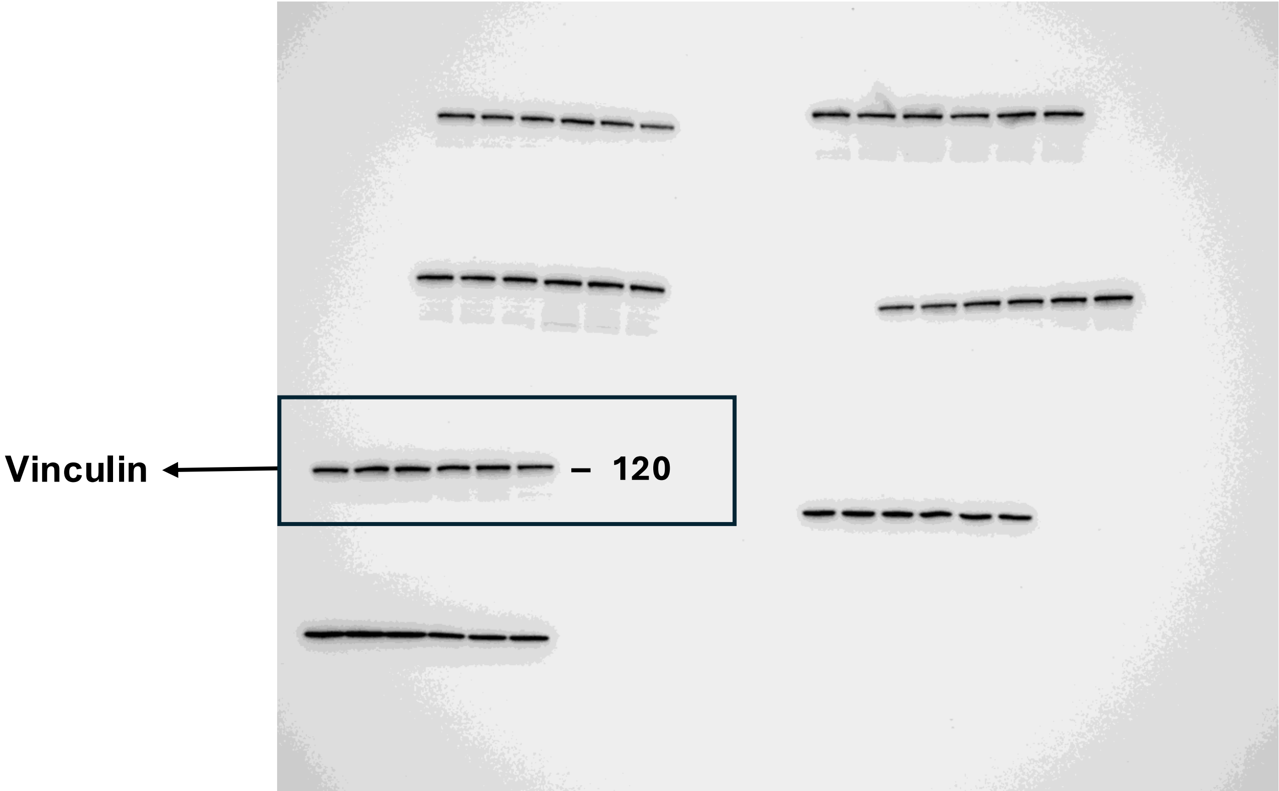

Figure 3C, 6G

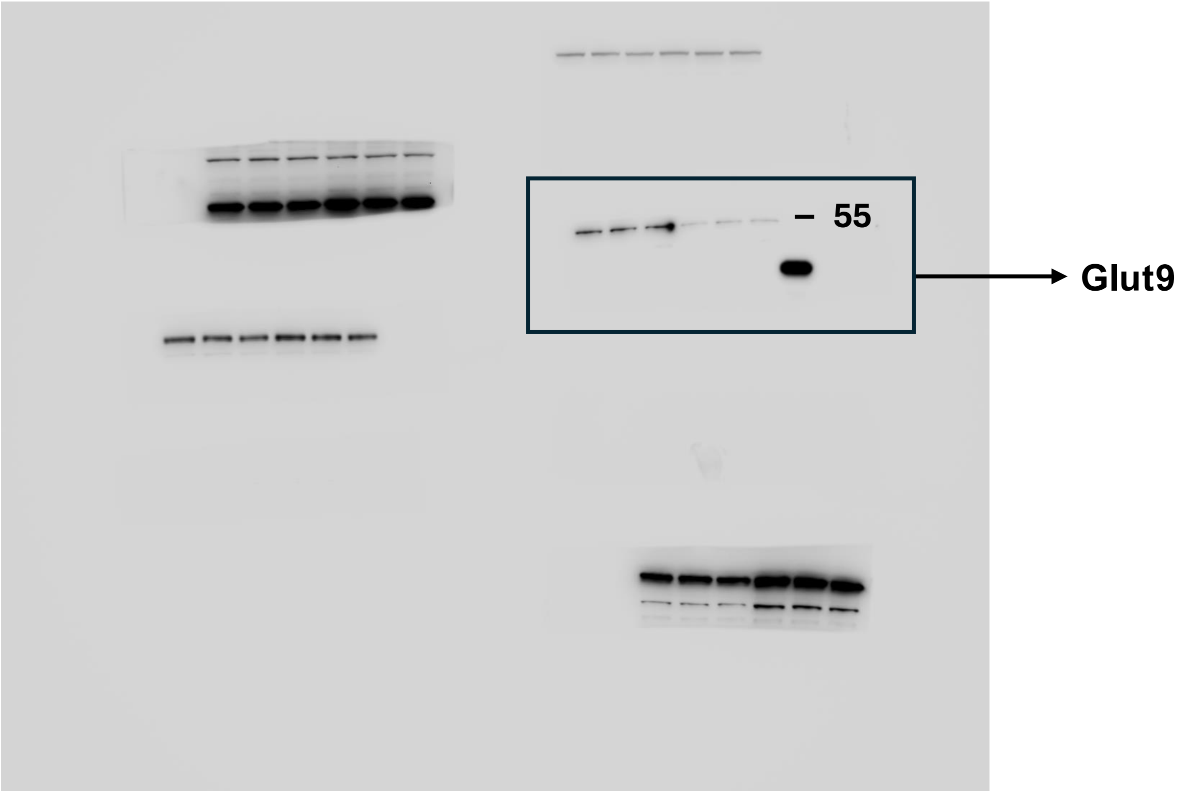

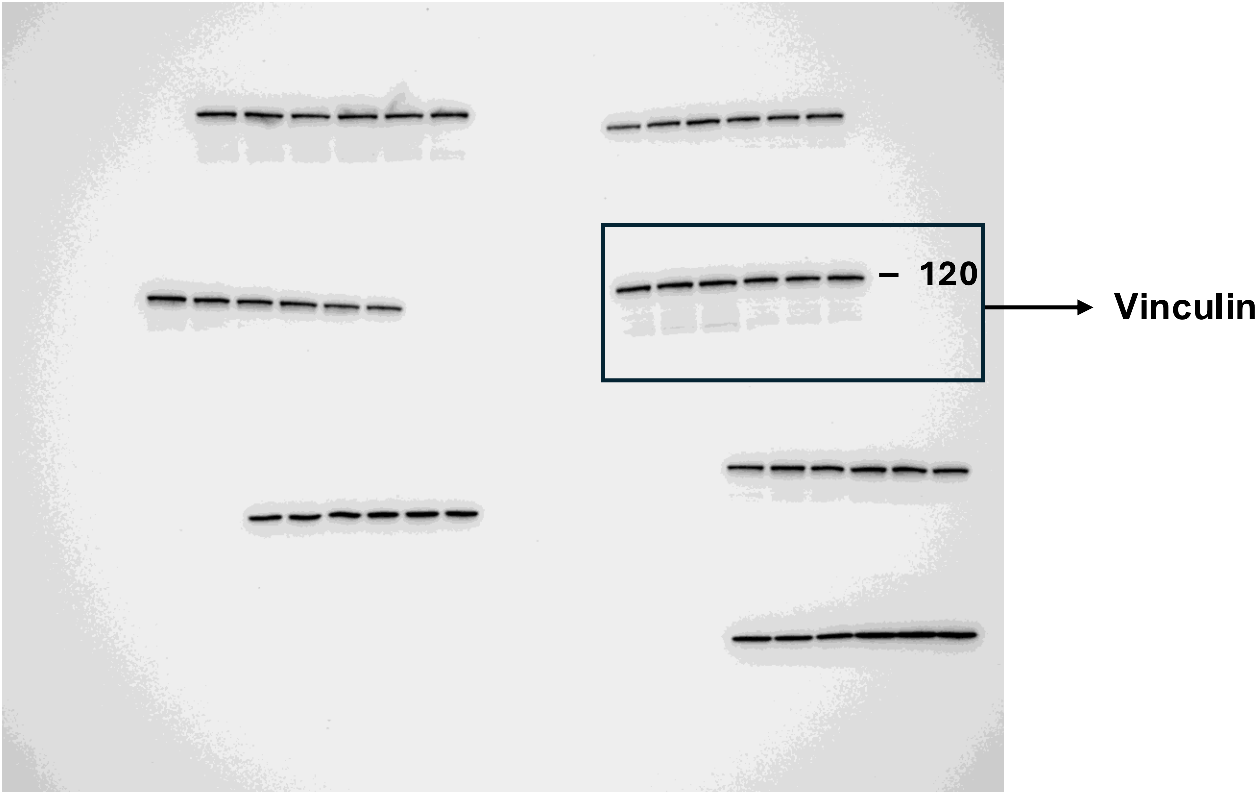

Figure 4C

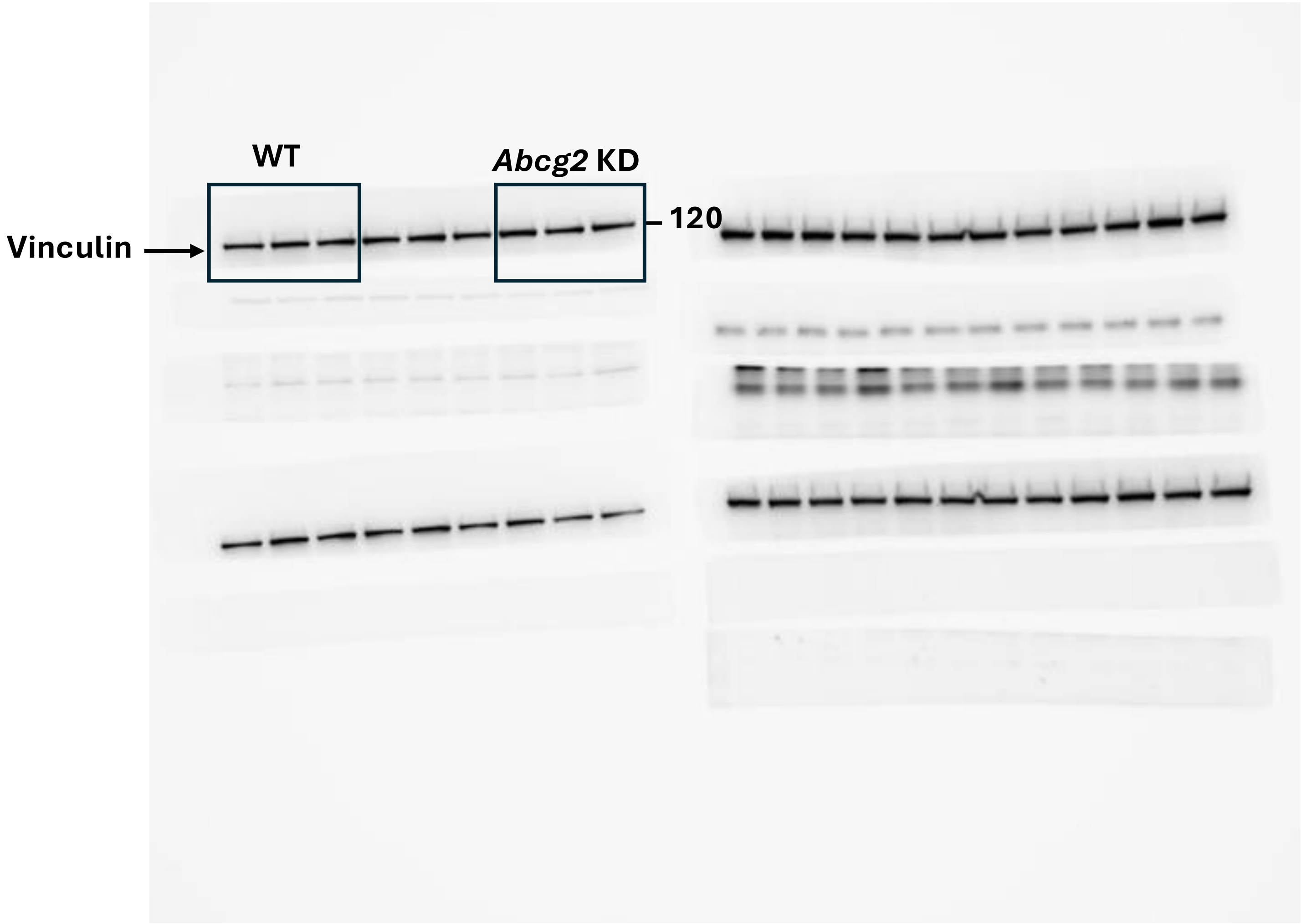

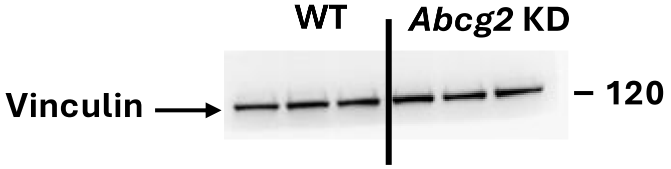

Lanes were run on the same gel but were noncontiguous

Figure 4C

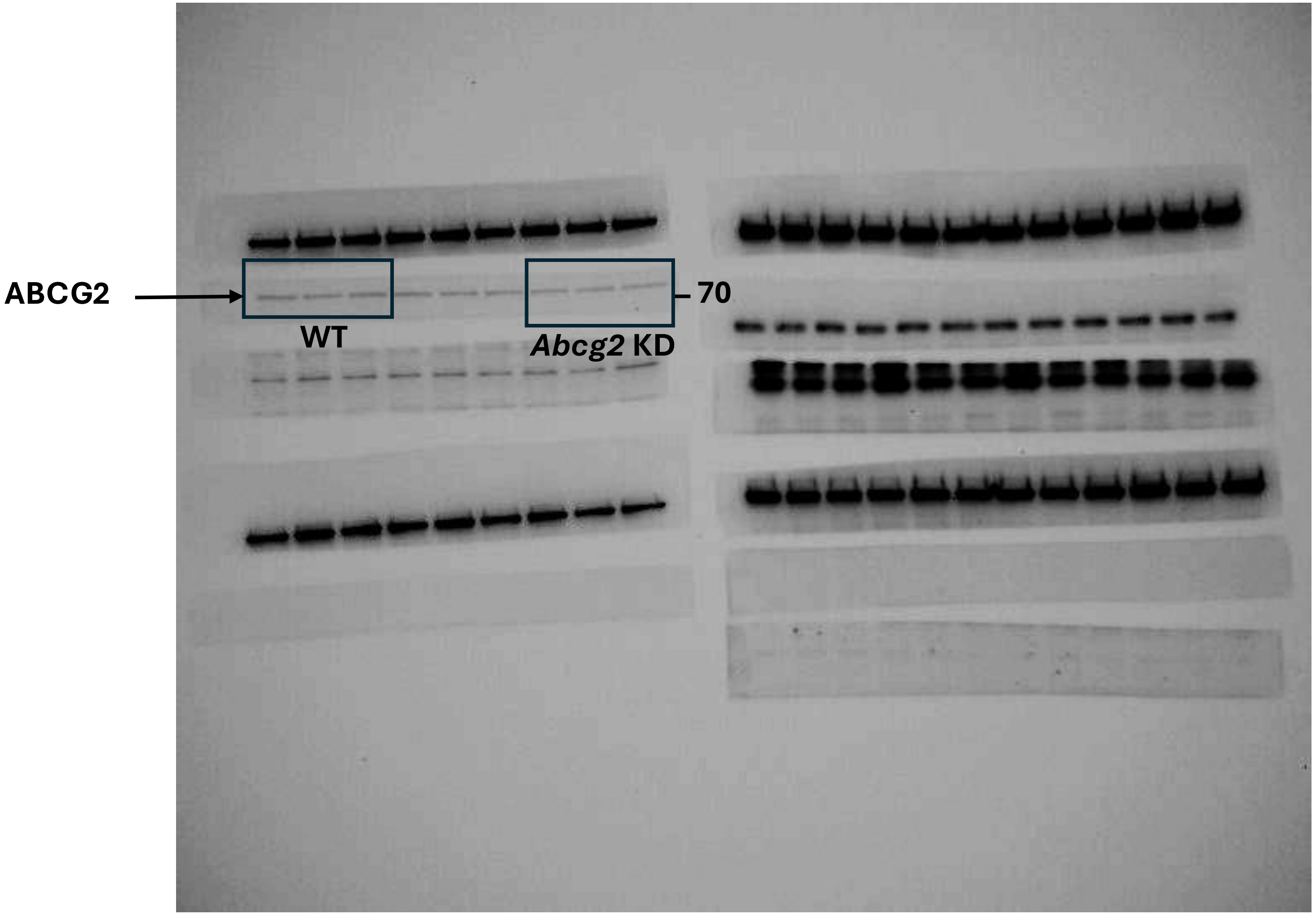

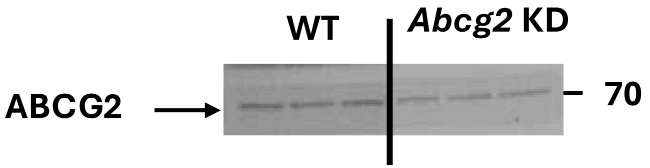

Lanes were run on the same gel but were noncontiguous

Figure 7A

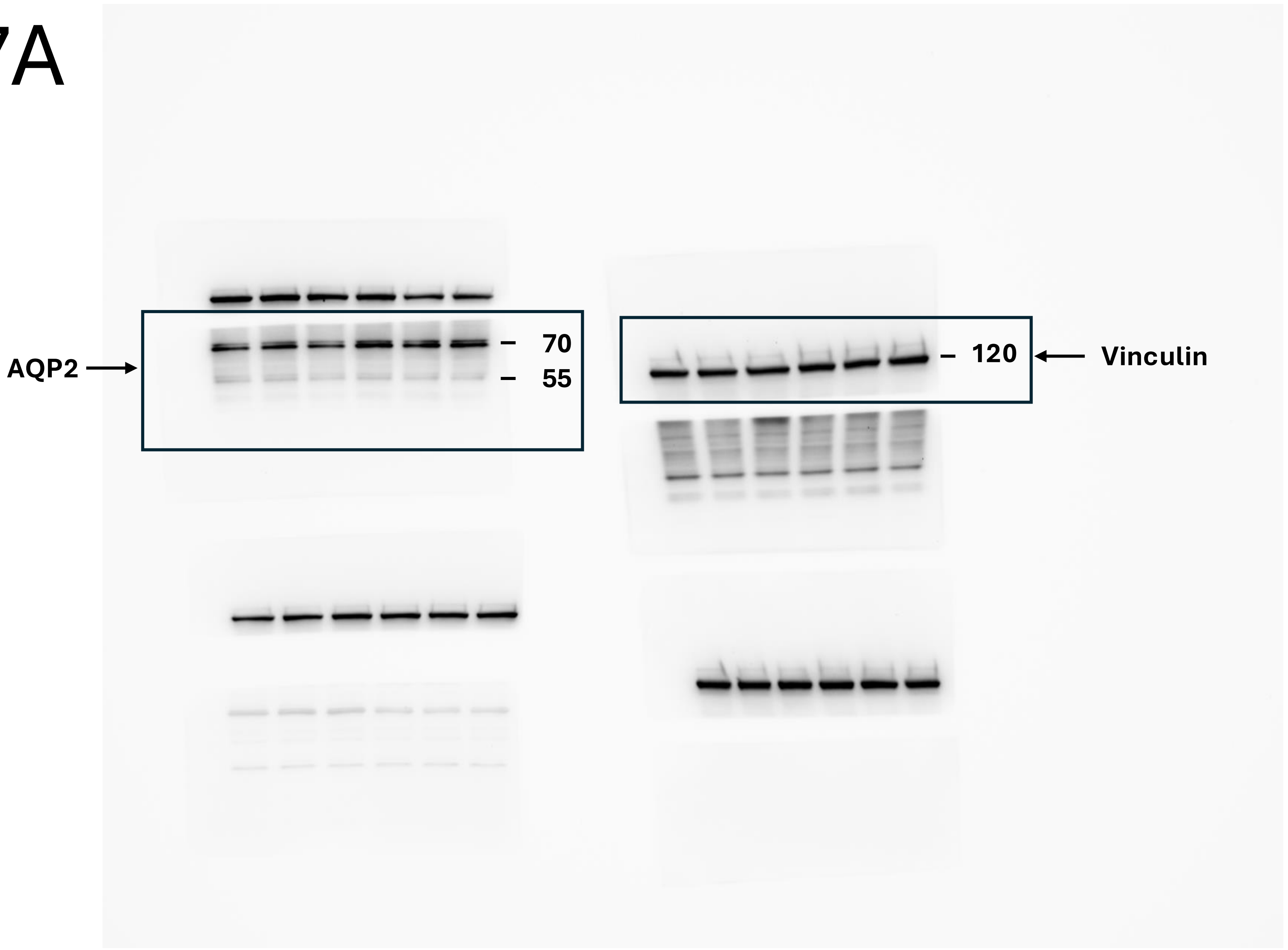

Figure 7A

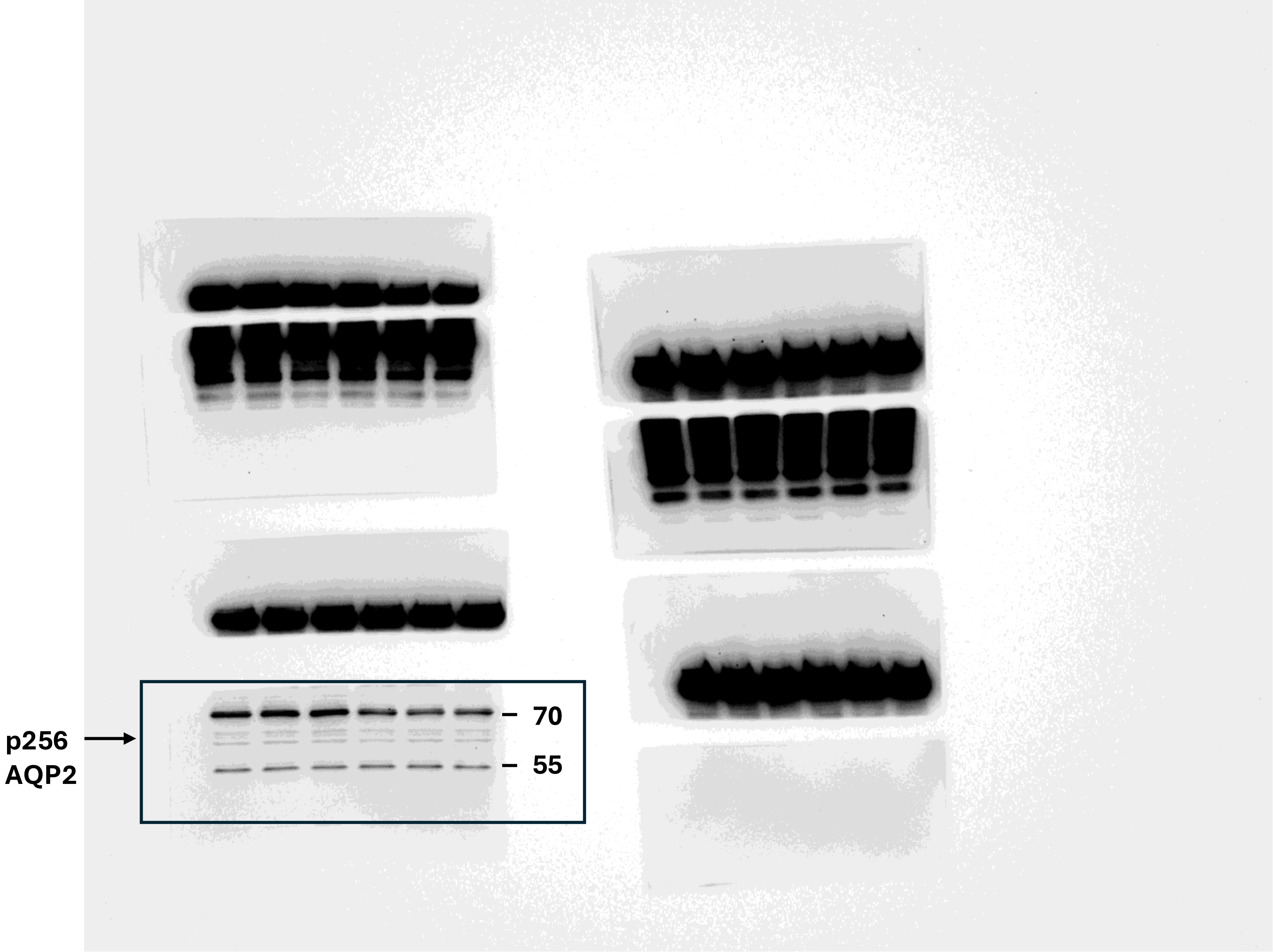

Figure 10D

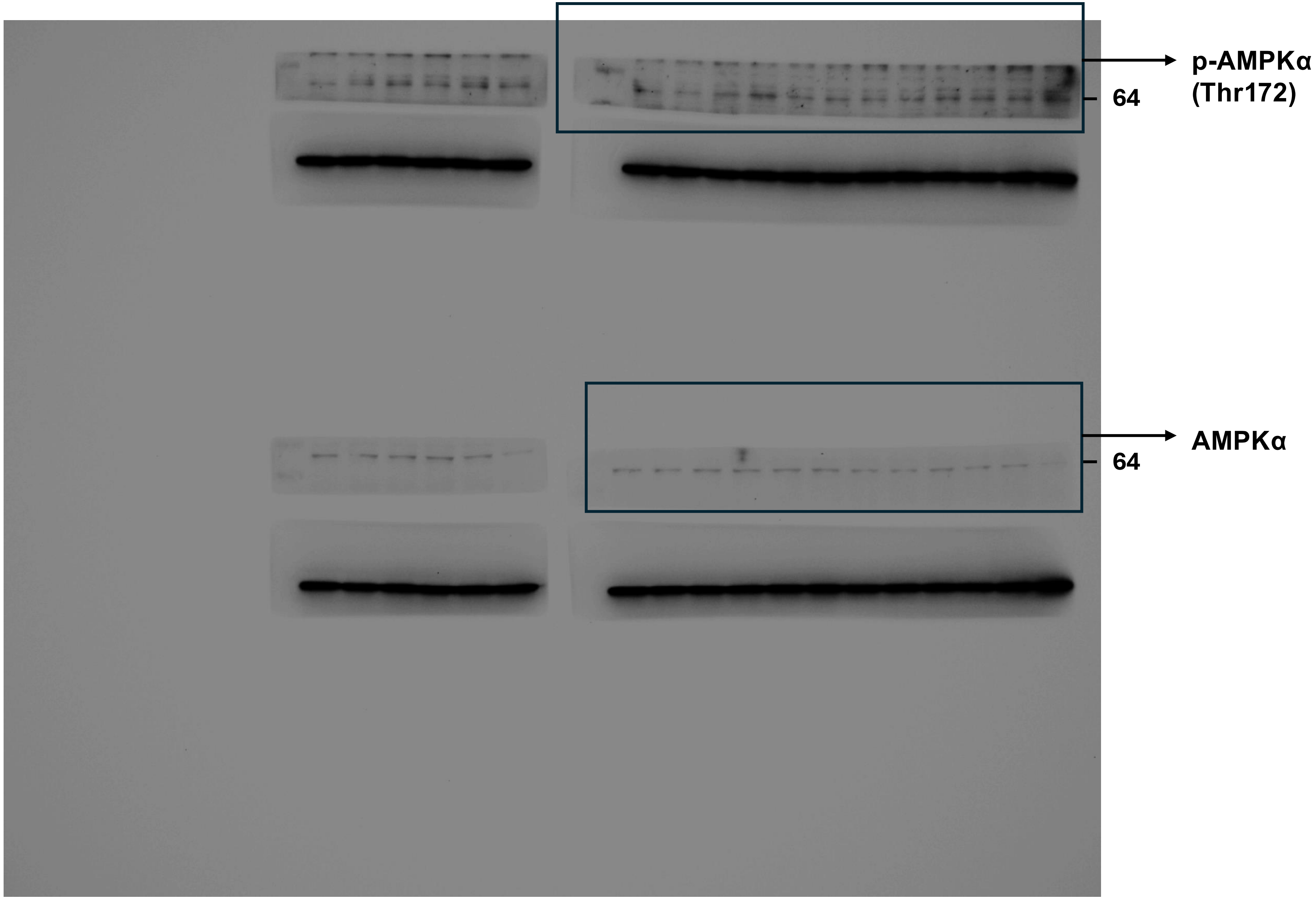

Figure 10D

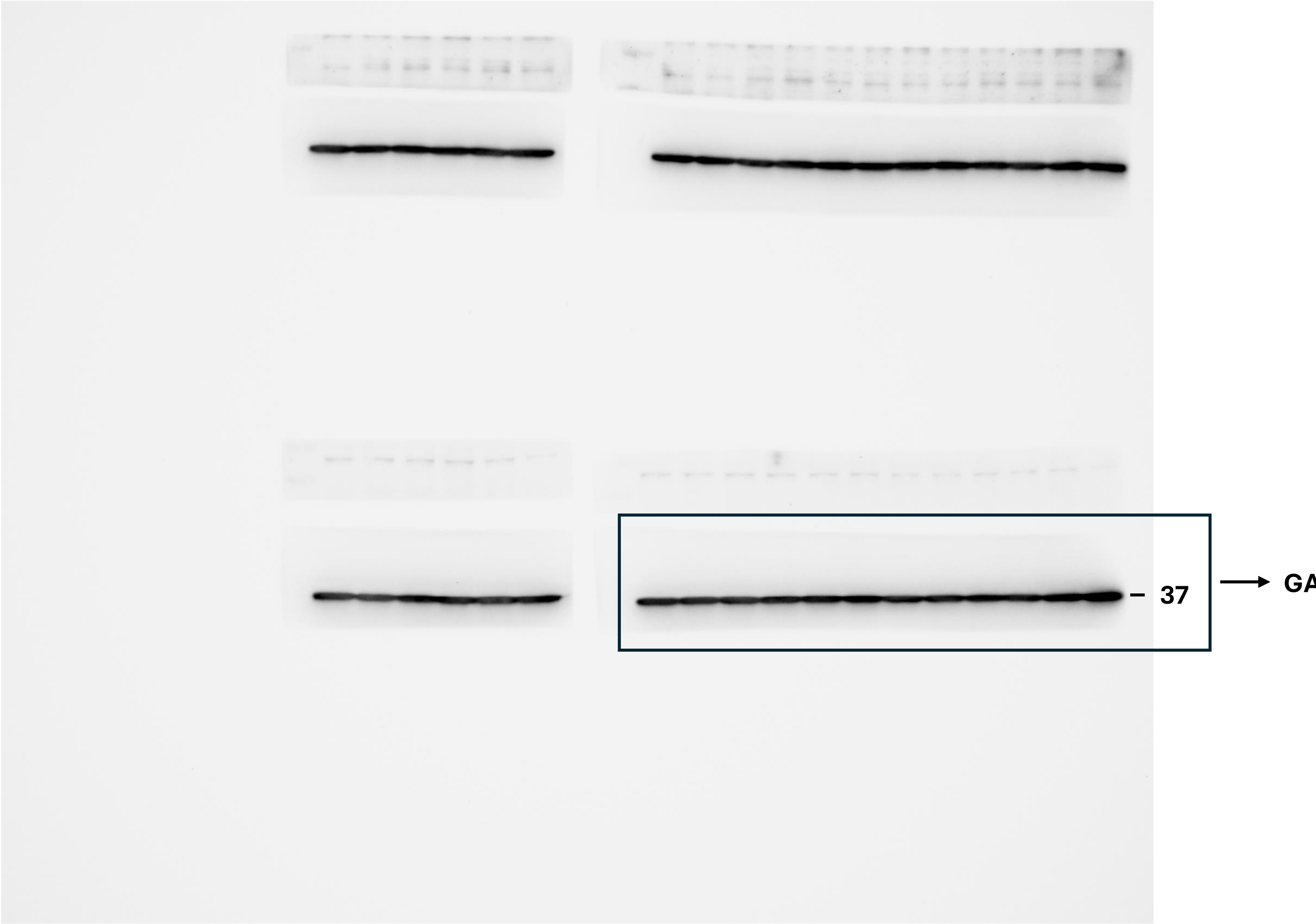

Figure 11C

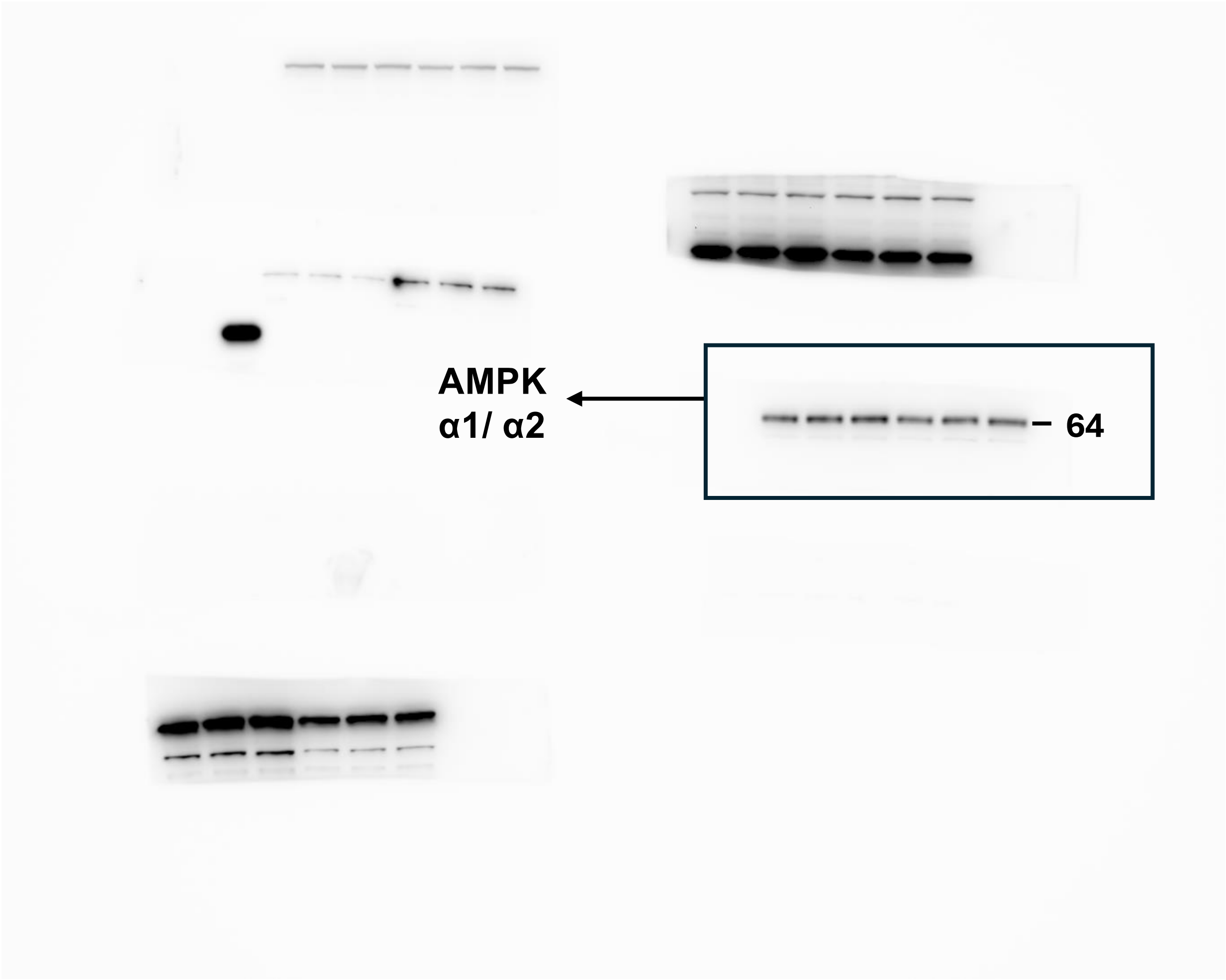

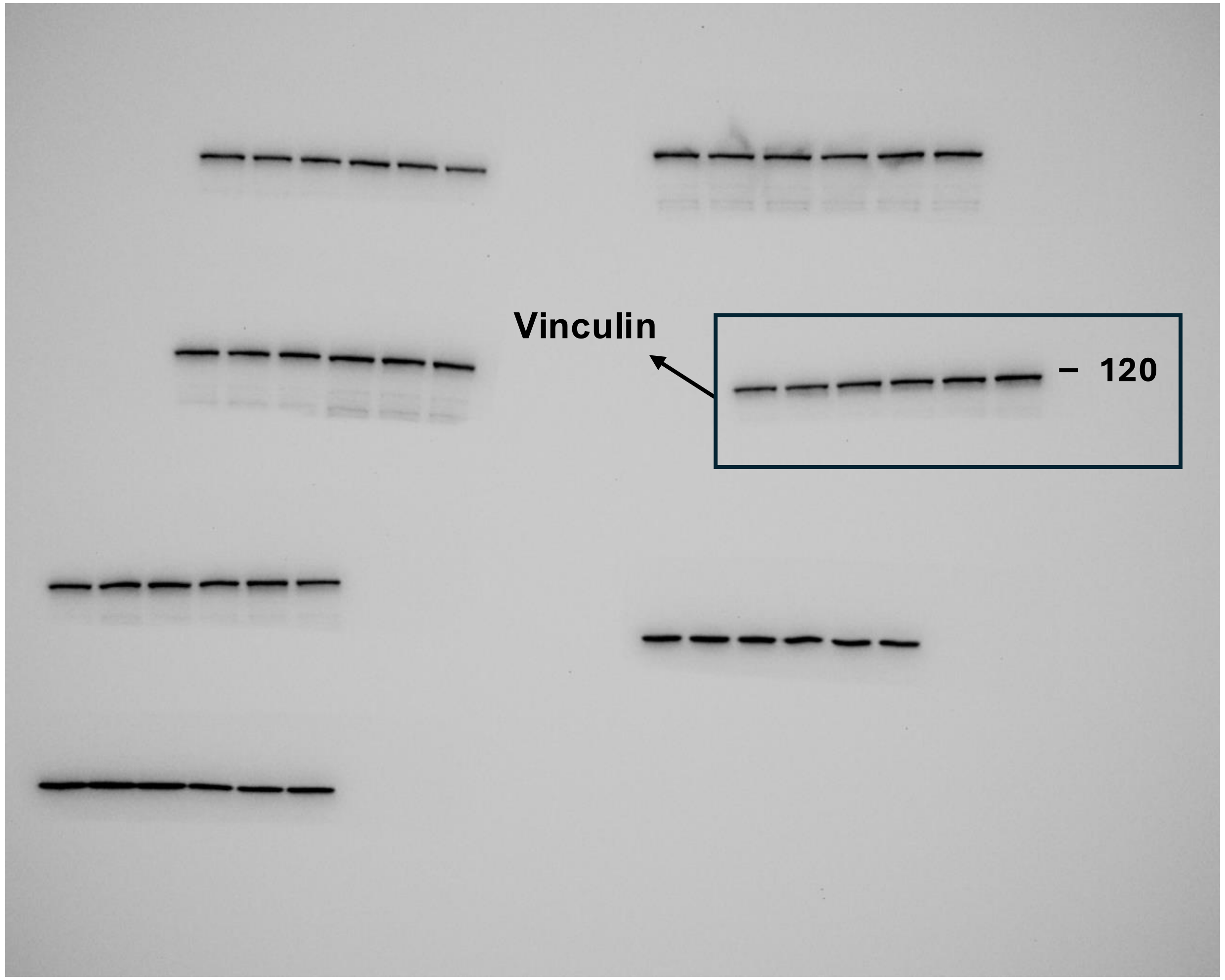

Supplementary Figure 4H

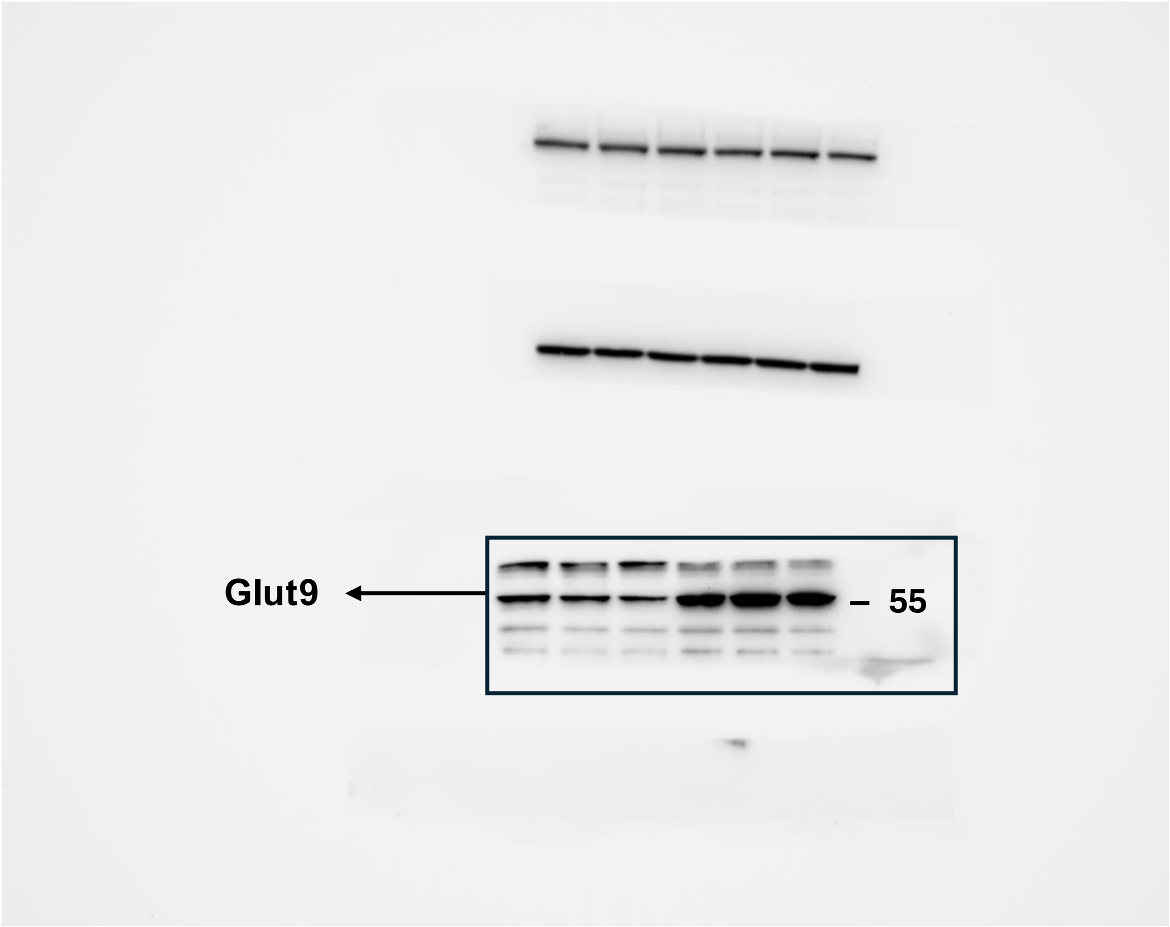

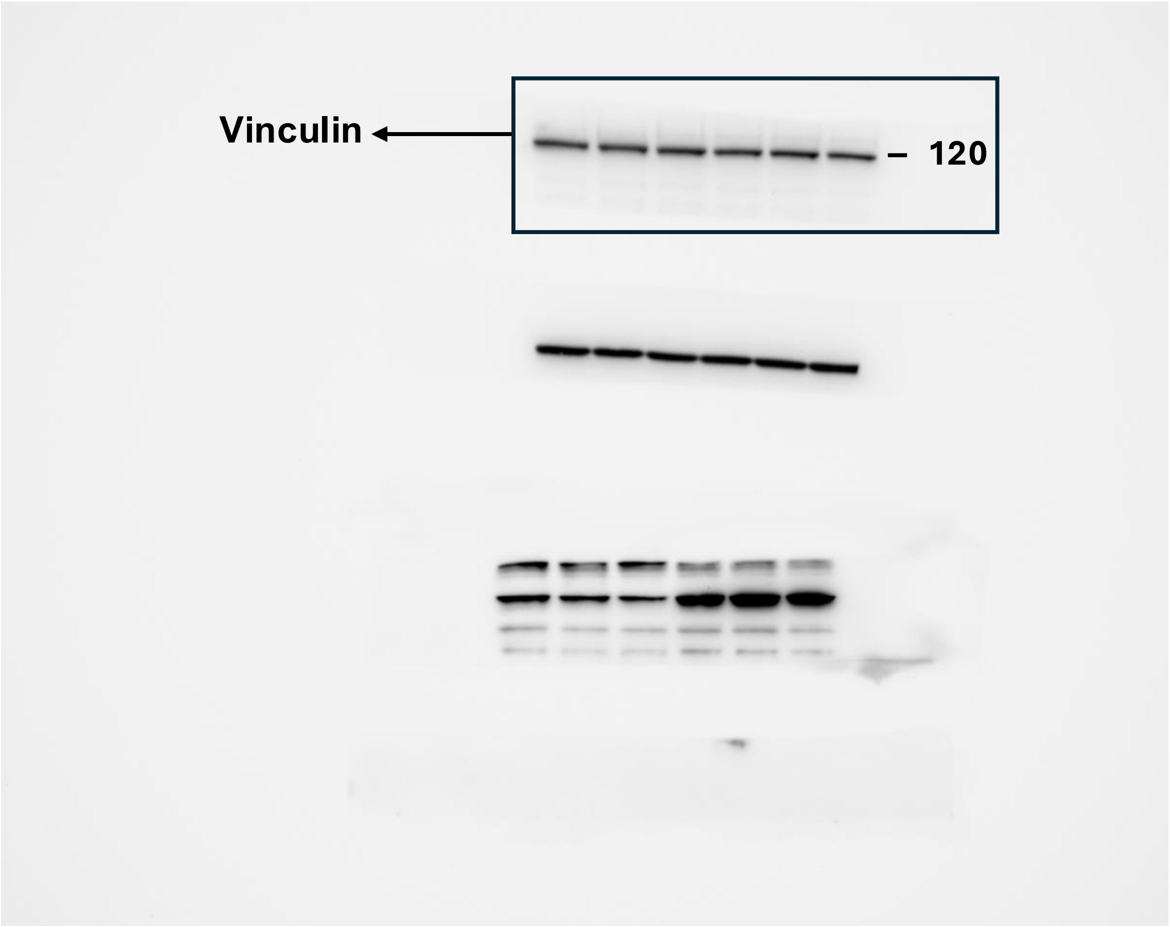

Supplementary Figure 4H

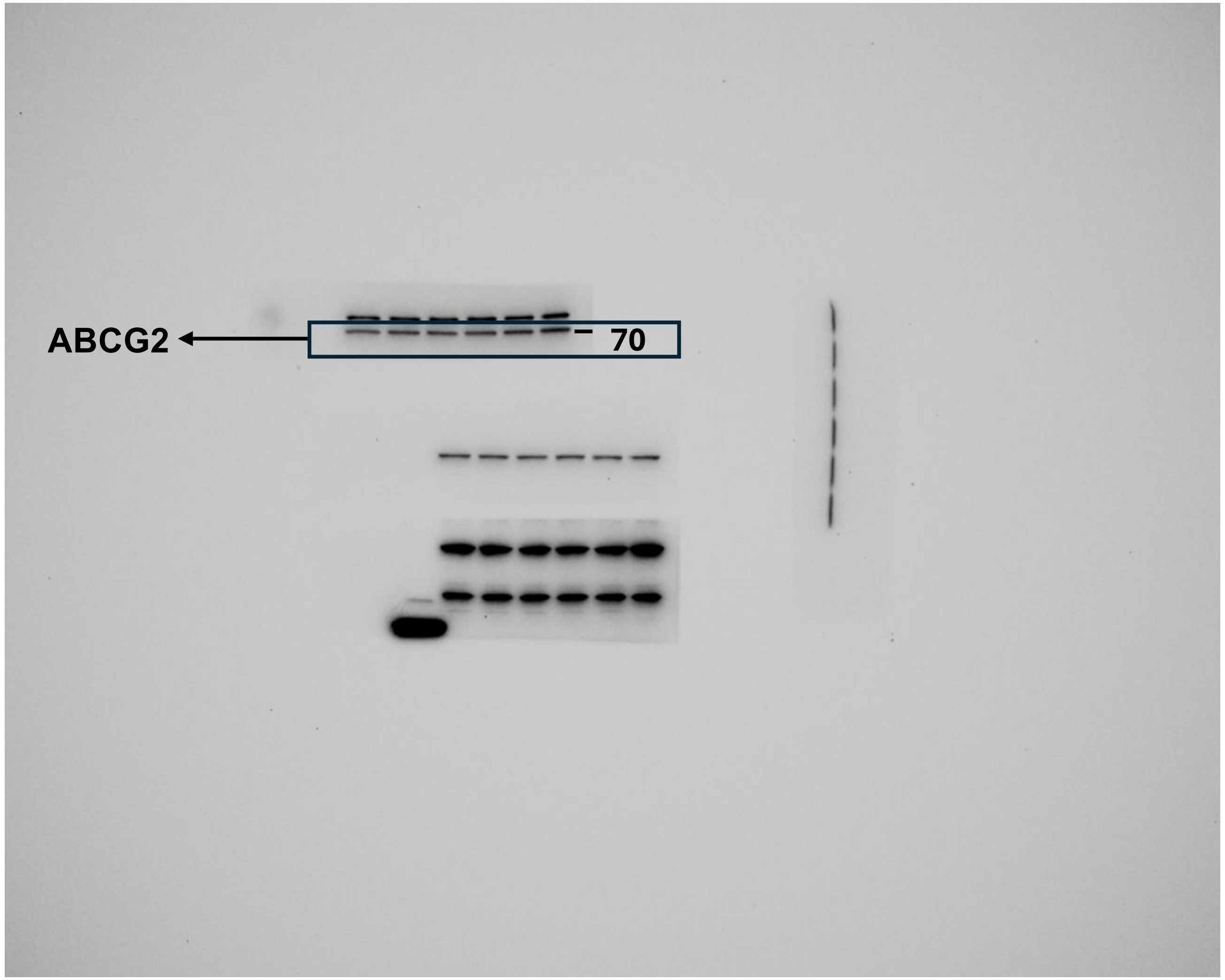

Supplementary Figure 5C

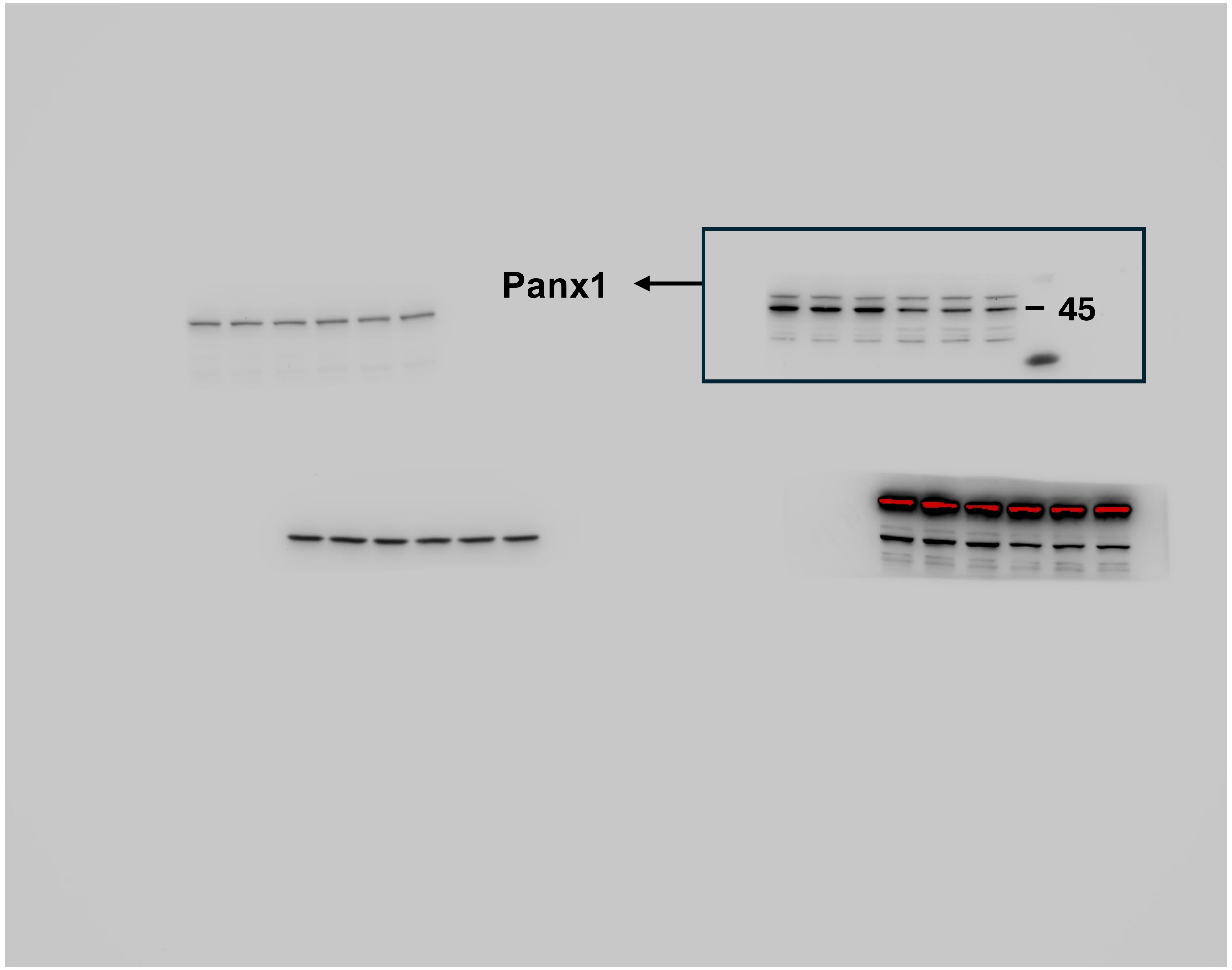

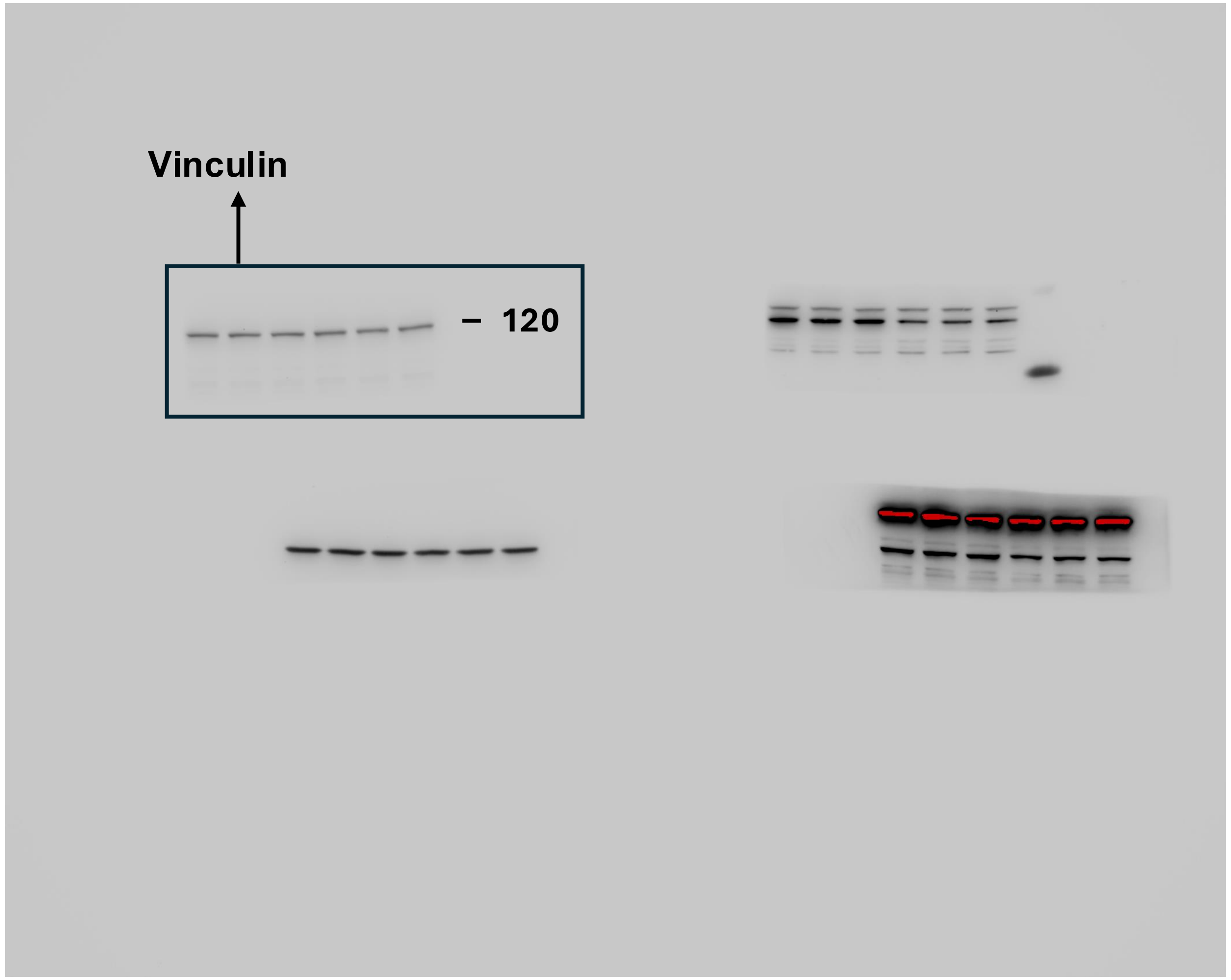

Supplementary Figure 5E

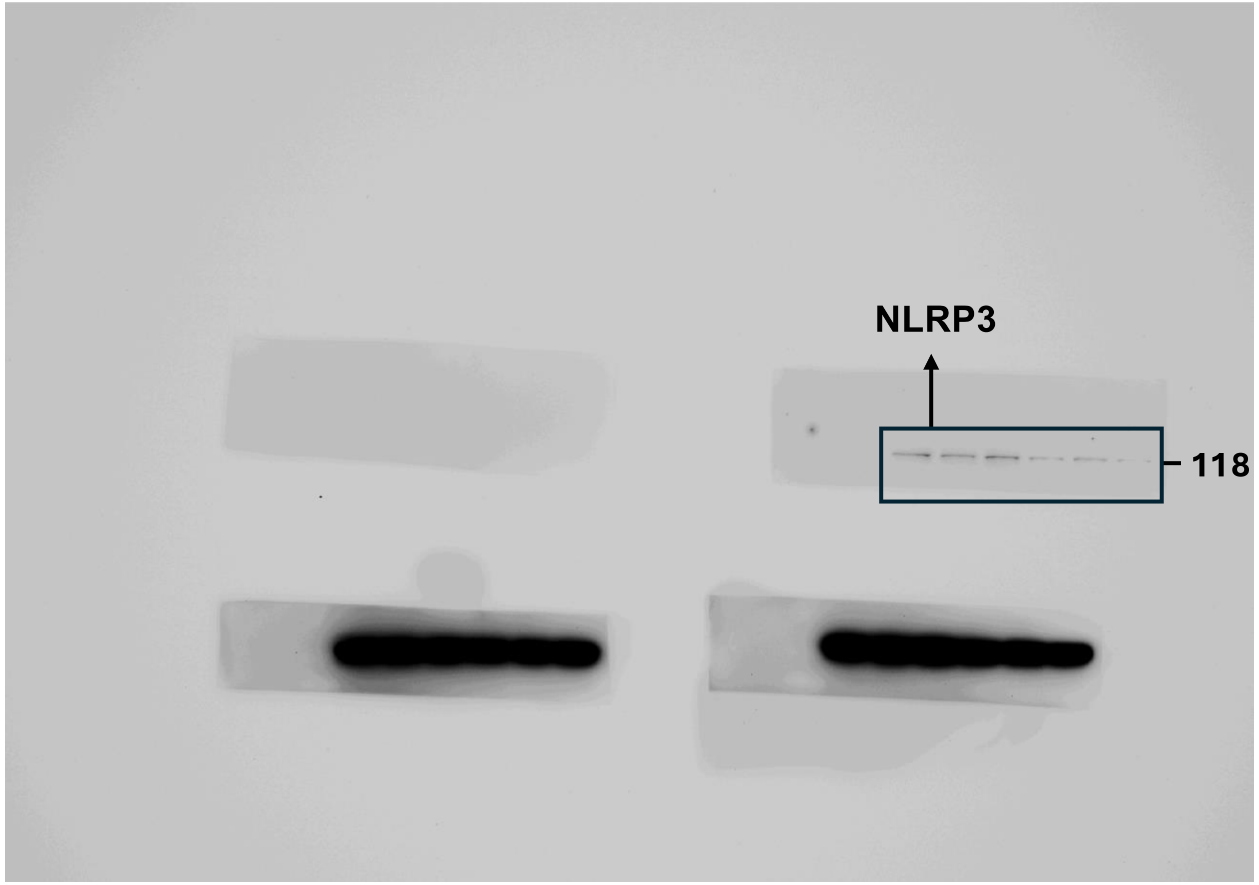

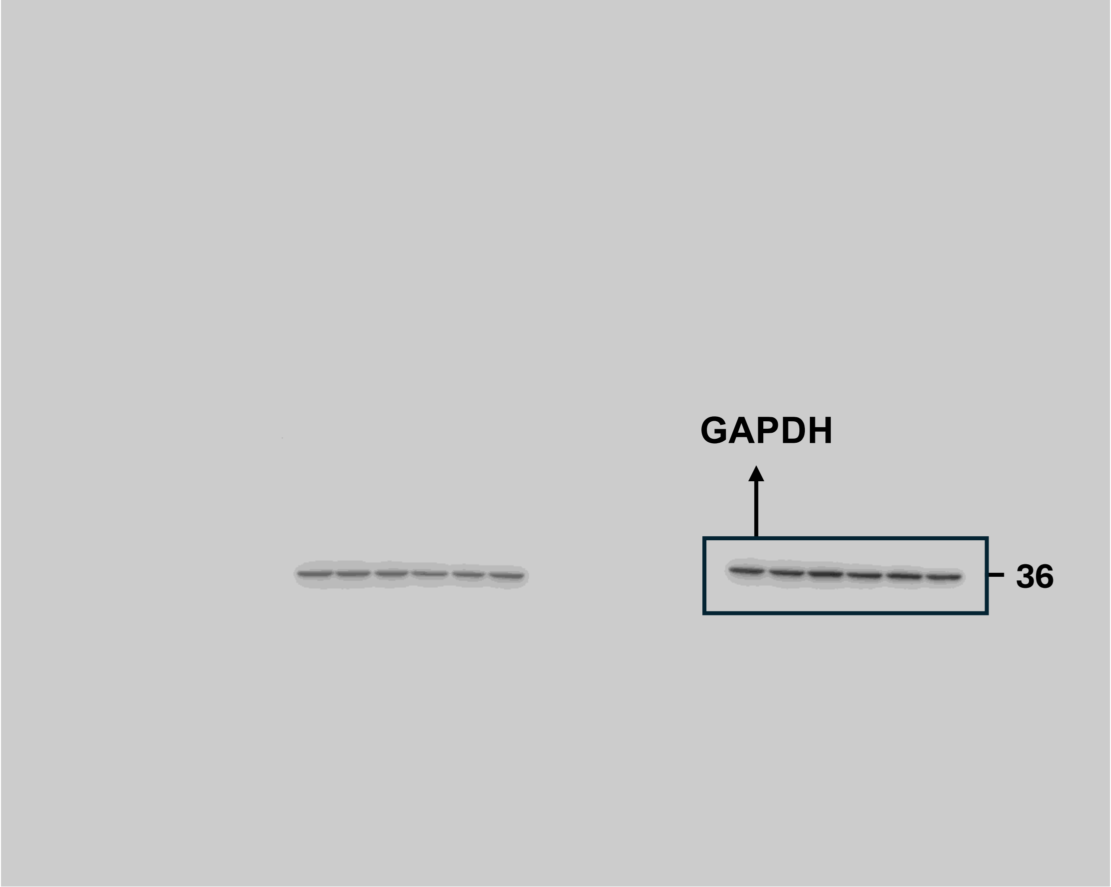

Supplementary Figure 6C

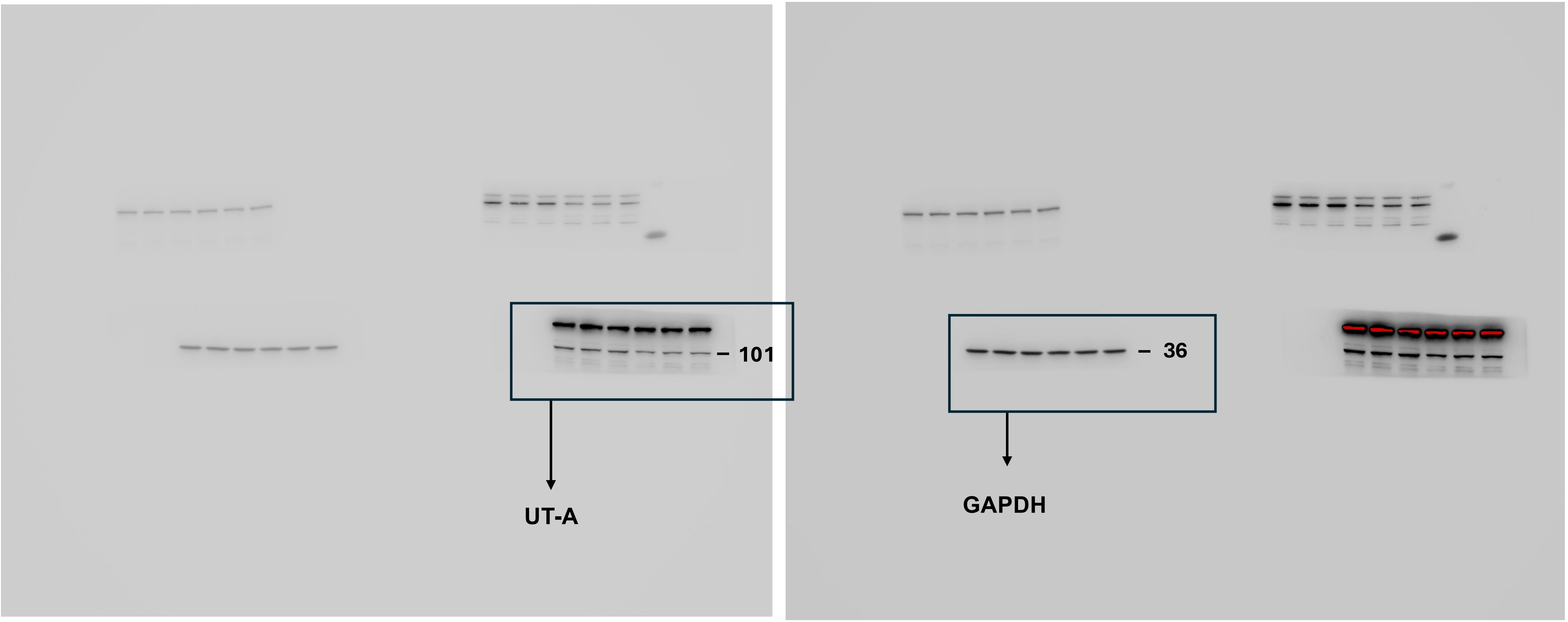

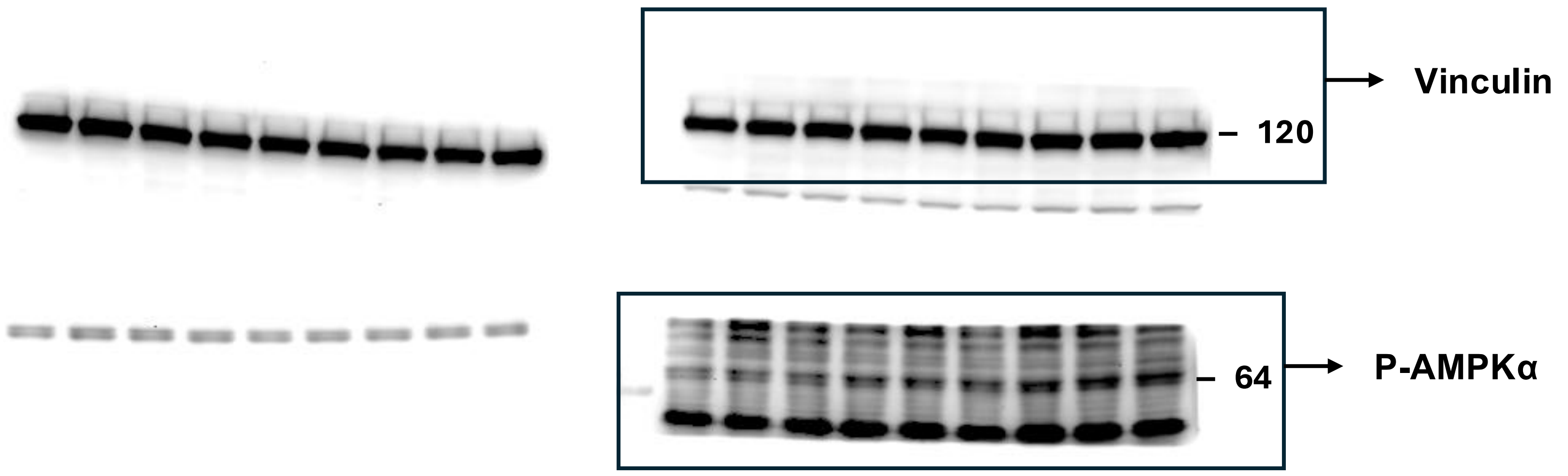

Supplementary Figure 7A

Supplementary Figure 7A

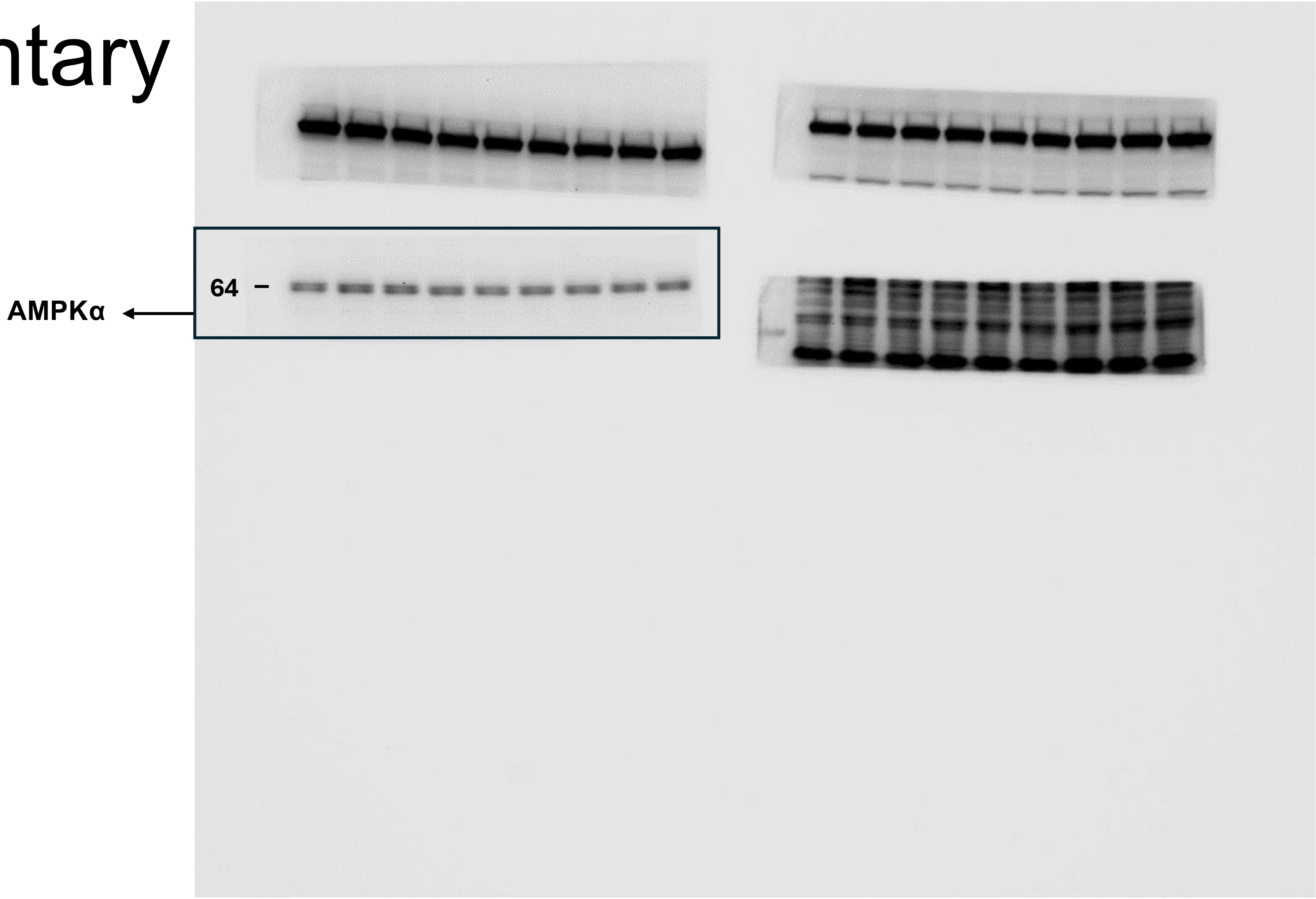

Supplement: Unedited blot and gel images [file jci-136-197021-s018.pdf]
